# Trends in mortality and causes of death among Chinese adolescents aged 10–19 years from 1990 to 2019

**DOI:** 10.3389/fpubh.2023.1075858

**Published:** 2023-02-07

**Authors:** Jiaxin Zhu, Yilu Li, Chengcheng Zhang, Jun He, Lu Niu

**Affiliations:** Department of Social Medicine and Health Management, Xiangya School of Public Health, Central South University, Changsha, China

**Keywords:** death causes, mortality, trends, China, adolescent, non-communicable diseases

## Abstract

**Objective:**

Promoting adolescent health is essential to achieving the goals of the Healthy China 2030 (HC 2030) initiative. As socioeconomic conditions improve and medical practices and disease patterns evolve, adolescent mortality rates and causes of death vary considerably. This study provides up-to-date data on adolescent mortality and causes of death in China, highlighting key areas of focus for investment in adolescent health.

**Methods:**

Data regarding mortality and causes of death in Chinese adolescents aged 10–19 years were extracted from the Global Burden of Disease study from 1990 to 2019. The data variables were examined according to year, sex, and age. The autoregressive integrated moving average model was used to predict non-communicable disease (NCD) mortality rates and rank changes in the leading causes of death until 2030.

**Results:**

The all-cause mortality rate (per 100,000 population) of Chinese adolescents aged 10–19 years steadily declined from 1990 (72.6/100,000) to 2019 (28.8). Male adolescents had a higher mortality (37.5/100,000 vs. 18.6 in 2019) and a slower decline rate (percent: −58.7 vs. −65.0) than female adolescents. Regarding age, compared with those aged 10–14 years, the mortality rate of adolescents aged 15–19 years had a higher mortality (35.9/100,000 vs. 21.2 in 2019) and a slower decrease rate (percent: −57.6 vs. −63.2). From 1990 to 2019, the rates of communicable, maternal, and nutritional diseases declined the most (percent: −80.0), while injury and NCDs mortality rates were relatively slow (percent: −50.0 and −60.0). In 2019, the five leading causes of death were road injuries (6.1/100,000), drowning (4.5), self-harm (1.9), leukemia (1.9), and congenital birth defects (1.3). Furthermore, NCDs' mortality rate decreased by −46.6% and −45.4% between 2015–2030 and 2016–2030, respectively.

**Conclusion:**

A notable decline was observed in all-cause mortality rates among Chinese adolescents aged 10–19 years. In addition, the mortality rates of NCDs are projected to meet the target from the Global Strategy for Women's, Children's, and Adolescents' Health (2016–2030) and HC2030 reduction indicators by 2030. However, it should be noted that injury is the leading cause of death, with sexual and age disparities remaining consistent.

## 1. Introduction

Adolescence, the time between childhood and adulthood (ages 10–19), is a distinct stage of human development and an important time for building a strong foundation for health (1, 2). To achieve a high standard of health, the World Health Organization (WHO) recently released the Global Strategy for Women's, Children's, and Adolescents' Health (2016–2030), which highlighted more strategic investments among adolescents aged 10–19 years (3, 4). The goal of this global strategy is to reduce premature mortality from non-communicable diseases in adolescents by one-third from 2016 to 2030, using mortality and cause of death as indicators (5–7). Similarly, China's Central Party Committee and State Council have approved the Health China 2030 (HC2030) initiative, highlighting adolescent health care (8). The HC2030 has a significant goal of decreasing premature mortality from NCDs, which include four major chronic diseases (e.g., neoplasms, cardiovascular diseases, chronic respiratory diseases, and diabetes and kidney diseases) (9). China currently accounts for approximately one-eighth of the world's adolescents and has one of the fastest mortality declines in Eastern Asia among adolescents aged 10–19 years (10, 11). To maintain and accelerate improvements in adolescent health, it is essential to provide a comprehensive assessment of the mortality burden for the 10–19-year-old age group in China.

Previous studies found that mortality and causes of death among adolescents vary significantly by sex and age. For mortality, a study reported that male adolescents aged 10–14 years in the European region had a higher mortality rate (24.1 vs. 15.8) and a slower decline rate (percent: −46.2 vs. −43.5) than that of female adolescents (12). In addition, two other studies (13, 14) discovered that the rate of deaths among older adolescents decreased at a slower pace compared to younger adolescents. Conversely, another study conducted in China found that the mortality rate among older adolescents decreased faster than that of younger adolescents (15). In terms of causes of death, the top five causes among adolescents aged 10–14 years in the European region were road injuries, drowning, leukemia, lower respiratory infections, and congenital birth defects in 2016 (12). Meanwhile, in China, the top five causes of death among adolescents aged 10–14 years in 2016 were road injuries, drowning, leukemia, congenital birth defects, and self-harm (15). In addition, compared with girls and younger adolescents, the top five causes of death in boys and older adolescents are more likely to be injury related (14, 15). Although these studies reported the mortality rates and the leading causes of death among adolescents aged < 19 years, some limitations persist. First, several studies (12, 14, 15) cannot provide the latest data due to using data from the Global Burden of Disease (GBD) 2016. Furthermore, these studies (12–15) did not assess the attainment of the target from the Global Strategy for Women's, Children's, and Adolescents' Health (2016–2030) and HC2030 regarding decreasing premature mortality from NCDs for the 10–19-year-old age group in China.

This study utilized data from the Global Burden of Disease (GBD) between 1990 and 2019 to examine the evolution of significant health issues among adolescents, identify current and future priorities for investing in adolescent health, and provide data-driven support. This study aimed to (1) analyze the trends of all-cause mortality and cause of death between 1990 and 2019; (2) assess the sex and age difference in all-cause mortality and cause of death from 1990 to 2019; and (3) predict NCD mortality rates and trends of the leading causes of death up to 2030.

## 2. Methods

### 2.1. Data sources

The data used in this study were extracted from the GBD 2019, a publicly accessible online database (16). Previous studies have provided detailed information on the design and methodology of the GBD (13, 17). This study analyzed metrics including death count, years of life lost, and death rate per 100,000 individuals with uncertainty intervals (UIs) by gender and age group, using data from 204 countries and territories. Additionally, the study included 281 causes of death at Level 4, as defined in the GBD 2019. Level 1 is divided into three categories: communicable, maternal, neonatal, and nutritional diseases (CMNN), non-communicable diseases (NCDs), and injuries. Level 2 further subdivides these categories into 21 causes of death, while levels 3 and 4 provide a more detailed breakdown.

Mortality rates from 281 causes were estimated for various locations, ages, and sexes between 1990 and 2019. The data used to make these calculations came from multiple sources, including vital registration systems, verbal autopsy reports, and surveillance data. The data regarding causes of death were corrected for any deficiencies or incompleteness. Detailed information regarding data processing has been previously reported (17). Point estimates for metrics originated from the mean of 1,000 draws from the posterior distribution of modeled cause-specific mortality. Their 95% UIs were estimated using the 2.5th and 97.5th percentiles of these 1,000 draws (17). The information about causes of death in China was predominantly derived from four data sources: disease surveillance points, the maternal and child surveillance system, the Chinese Center for Disease Control and Prevention's cause-of-death reporting system, and cancer registry data (18).

### 2.2. Data presentation

This study targeted adolescents aged 10–19 years in China and reported 142 causes of death from Levels 1, 2, and 3 (12). To compare the mortality rates of adolescents in countries with different incomes, we extracted all-cause mortality rates from high-income countries, upper-middle-income countries, lower-middle-income countries, and lower-income countries based on the World Bank's classification (19). We presented death numbers, rates, and percentages and described their percentage changes between 1990 and 2019. However, we only presented the death percentage at Levels 1 and 2 causes of death. To illustrate the difference, we compared the proportion of deaths for Levels 1 and 2 causes of death between 1990 and 2019 by calculating their percentage differences. The corresponding formulae are presented in Tables S4–S8 in Supplementary material 1. The death percentage was defined as the proportion of deaths caused by a specific cause compared to all causes. Percentage changes were deemed statistically significant if the 95% UI did not contain zero. The death rate indicates the number of deaths per 100,000 people.

### 2.3. Prediction model

One of the Global Strategy's targets is to reduce premature mortality from NCDs by one-third from 2016 to 2030, including the indicator of causes of death (4). Similarly, the HC2030 includes the goal of reducing mortality from NCDs (especially for four major chronic diseases) by one-third from 2015 to 2030 in all age groups (9). Due to word limitations, only the mortality predictions of NCDs between 2016 and 2030 are discussed in this article's main text, and the remaining parts are shown in Tables S9–S11 in Supplementary material 1.

To evaluate the probability of meeting these indicators based on the mean mortality rates between 1990 and 2019, we utilized the autoregressive integrated moving average model (ARIMA). This model is a time-series analysis method used to forecast future series according to past information. This study was used to forecast the mortality rates of NCDs and the number of deaths due to different causes among adolescents up to 2030, illustrating the changes in the leading cause of death in China from 2020 to 2030. The optimal parameters of ARIMA could be determined by Akaike's information criterion or Schwarz's Bayesian criterion (20). The Ljung-Box test examines autocorrelation and mean absolute percent error (MAPE) and is a common metric to test the model's predictive validity. ARIMA's modeling includes autoregression, a moving average, and a difference (20). The model is generally denoted as ARIMA (p, d, q), in which p, d, and q refer to the autoregression, difference, and moving average, respectively (20). Supplementary material 2 displays the assumptions and principles of the ARIMA model in detail.

In this study, the “auto.Arima” function from the R package “forecast version 8.12” was used to ensure the parameters of the models to forecast the death rate and death number for each cause of death (21). The autocorrelation of the model residuals was examined using the Ljung–Box test and resulted in a *P* > 0.05, indicating that the residuals were white noise. The model's validity was evaluated using data from 1990 to 2013 to forecast the mortality rates from 2011 to 2017 and calculate MAPE (22). The exp function was used for the death number to transform and calculate the fitted and predicted values (23). All analyses and figures were performed using R (version 4.1.5) and Origin 2022.

## 3. Results

### 3.1. The mortality trends of Chinese adolescents aged 10–19 years from 1990 to 2019

**Figure 1:**
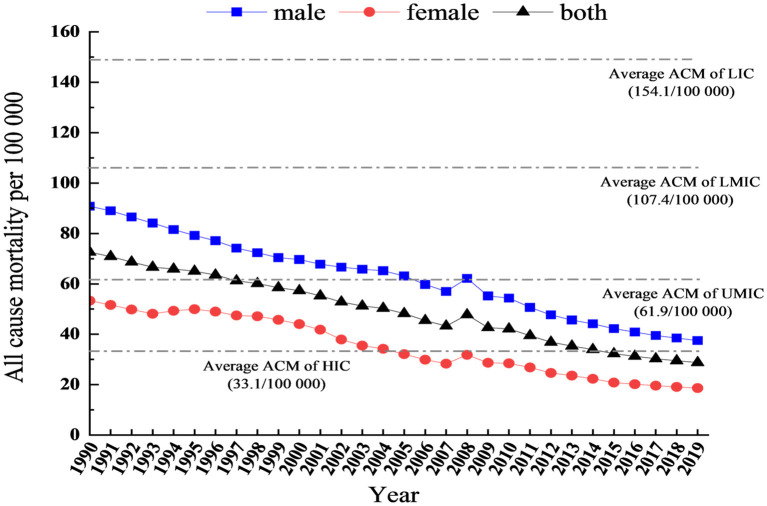
The trends of all causes of mortality rates in China, aged 10–19 years. ACM, All-cause mortality; HIC, High-income countries; UMIC, Upper-middle income countries; LMIC, Lower-middle income countries; LIC, Lower income countries.

We estimated 41,956 deaths (95% UI 36,861–47,242) among adolescents aged 10–19 years, and the all-cause mortality was 28.8 per 1000,000 (95% UI 27.9–29.7) in 2019. Between 1990 and 2019, all-cause deaths declined in all adolescents (percent change: −70.0%, 95% UI −70.0% to −80.0%). Similarly, the all-cause mortality rates reflected a 60.0% decline (95% UI −50.0% to −70.0%) during this period (Table 1). As shown in Figure 1, the all-cause mortality rates for Chinese adolescents decreased from 1990 to 2019, both in general and for the different sexes, except for a slight increase in 2008.

**Table 1:** The mortality rates, deaths, and changes for all causes of death among adolescents by sex and age from 1990 to 2019.

**Group**	**Number of deaths**			**Mortality rate (per 100,000 people)**		
	**1990**	**2019**	**Percentage change (%)**	**1990**	**2019**	**Percentage change (%)**
**Age**						
10–14	59,365 (54,192 to 65,172)	14,998 (13,514 to 16,497)	−74.7 (−78.1 to −71.2)	57.8 (52.7 to 63.4)	21.2 (19.1 to 23.3)	−63.2 (−68.2 to −58.1)
15–19	107,338 (120,442 to 94,679)	26,958 (23,222 to 30,885)	−74.9 (−79.1 to −69.8)	84.6 (74.6 to 94.9)	35.9 (30.9 to 41.1)	−57.6 (−64.7 to −49.0)
**Sex**						
Male	107,308 (93,011 to 121,674)	29,406 (24,735 to 34,540)	−72.6 (−77.9 to 65.9)	90.8 (78.7 to 102.9)	37.5 (31.5 to 44.0)	−58.7 (−66.8 to −48.5)
Female	59,395 (51,526 to 69,662)	12,550 (10,893 to 14,250)	−78.9 (−82.7 to 74.7)	53.3 (46.2 to 62.5)	18.6 (16.1 to 21.2)	−65.0 (−71.4 to −58.2)
Total	166,703 (148,841 to 185,450)	41,956 (36,861 to 47,242)	−70.0 (−70.0 to −80.0)	72.6 (71.8 to 73.4)	28.8 (27.9 to 29.7)	−60.0 (−50.0 to −70.0)

Furthermore, Figure 1 presents a comparison of adolescent mortality rates across different income countries. In 2019, compared with different income countries, the mortality rates of Chinese adolescents (28.8 per 100,000) were lower than the average mortality rates of high-income countries (33.1 per 100,000), upper-middle-income countries (61.9 per 100,000), lower-middle-income countries (107.4 per 100,000), and low-income countries (154.1 per 100,000).

Regarding sex differences, the deaths were higher in boys (29,406, 95% UI 24,735–34,540) than in girls (12,550, 95% UI 10,893–14,250) (Table 1). Similarly, in 2019, for boys, the mortality rate was 37.5 (95% UI 31.5–44.0), which was almost two times as high as that for girls (18.6, 95% UI 16.1–21.2). Furthermore, the number of deaths and death rates in boys declined more than in girls (percent change: −72.6% and −58.7% vs. −8.9% and −65.0%) (Figure 1; Table 1).

For age group differences, deaths were higher among adolescents aged 15–19 years (26,958, 95% UI 23,222–30,885) than in those aged 10–14 years (14,998, 95% UI 13,514–16,497) (Table 1). Similarly, the all-cause mortality rate among adolescents aged 15–19 years was 35.9 (95% UI 30.9–41.1), which is higher than in those aged 10–14 years (21.2, 95% UI 19.1–23.3). The death rates in those aged 15–19 years decreased further than in those aged 10–14 years (percent change: −57.6% vs. −63.2%) (Table 1).

### 3.2. Changes in the proportion of causes of death and leading causes of death from 1990 to 2019

**Figure 2:**
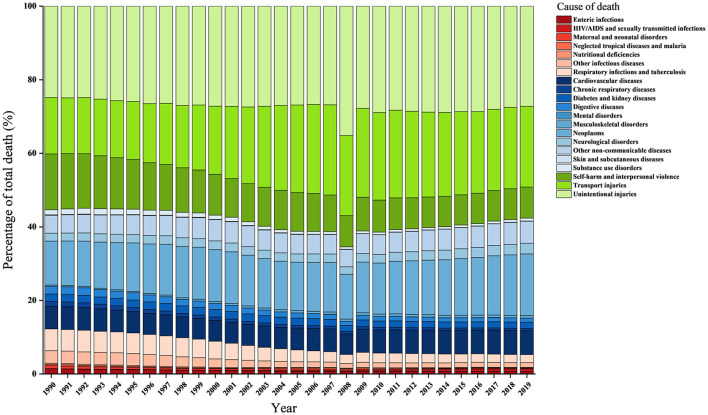
Percentage of total deaths by level-2 causes in China, aged 10–19 years, both sexes from 1990 to 2019. All green bars are classified as injuries. All blue bars are classified as non-communicable diseases. All red bars are classified as communicable, maternal, neonatal, and nutritional diseases.

Figure 2 shows the proportion of causes of death at Levels 1 and 2 between 1990 and 2019. At Level 1, injuries accounted for the largest proportion of death causes, particularly in 2008, followed by NCDs; CMNN accounted for the smallest proportion of death causes from 1990 (55.4% vs. 32.4% vs. 12.2%) to 2019 (57.6% vs. 37.1% vs. 5.3%). From 1990 to 2019, CMNN reported the biggest drop in the number of adolescent deaths, and NCDs had the biggest rise in the number of adolescent deaths (Table 2, difference: −6.9 vs. 4.7 vs. 2.2).

**Table 2:** The proportion for three categories of adolescents aged 10–19 years in China, 1990 and 2019.

**Group**	**Proportion of all causes of death (95%UI)**		**Difference (%)**
		**1990**	**2019**
**Total**			
Injuries	55.4% (53.7%−29.1%)	57.6% (55.4%−59.0%)	2.2
NCDs	32.4% (29.9%−33.6%)	37.1% (35.9%−39.0%)	4.7
CMNN	12.2% (10.5%−13.8%)	5.3% (4.8%−5.9%)	−6.9
**Male**			
Injuries	60.0% (58.0%−65.3%)	62.3% (59.1%−63.7%)	2.3
NCDs	29.8% (26.2%−31.3%)	33.2% (31.8%−35.8%)	3.4
CMNN	10.2% (8.2%−11.6%)	4.5% (4.1%−5.2%)	−5.7
**Female**			
Injuries	47.0% (45.2%−48.7%)	46.7% (45.1%−47.8%)	−0.3
NCDs	37.2% (35.6%−38.5%)	46.2% (44.8%−47.6%)	9.0
CMNN	15.8% (14.3%−18.1%)	7.2% (6.6%−8.0%)	−8.6
**Total (10–14)**			
Injuries	54.4% (52.4%−58.0%)	56.5% (53.8%−58.3%)	2.1
NCDs	30.5% (28.5%−31.9%)	36.9% (35.2%−39.3%)	6.4
CMNN	15.6% (12.8%−17.1%)	6.6% (6.0%−7.5%)	−9.0
**Total** **(15–19)**			
Injuries	55.9% (54.1%−59.9%)	58.2% (55.7%−59.6%)	2.3
NCDs	33.5% (30.5%−34.8%)	37.2% (36.0%−39.4%)	3.7
CMNN	10.7% (9.1%−12.4%)	4.5% (4.2%−5.1%)	−6.2

Regarding differences in sex among the causes of death at Level 1, injuries constituted the highest proportion in boys, comprising more than half of the deaths; particularly in 2019, almost two-thirds of the deaths in male adolescents were caused by injuries (60.0% in 1990 and 62.3% in 2019). For girls, injuries accounted for the largest proportion, which was slightly higher than NCDs (46.7% vs. 46.2%) in 2019. Between 1990 and 2019, injuries and NCDs in boys contributed more to total deaths (difference: 2.3 vs. 3.4 vs. −5.7). By contrast, only NCDs in girls reported an increase in the contribution of total deaths (difference: 9.0 vs. −0.3 vs. −8.6) (Table 2; Supplementary Figures S1, S2).

For the two age groups (i.e., 10–14 years and 15–19 years), injuries accounted for the largest proportion of death causes, followed by NCDs; CMNN accounted for the smallest proportion of death causes from 1990 to 2019. From 1990 to 2019, CMNN reported the largest reduction in the contribution of total deaths, and NCDs revealed the highest increase in the contribution of total deaths (Table 2; Supplementary Figures S3, S4).

**Figure 3:**
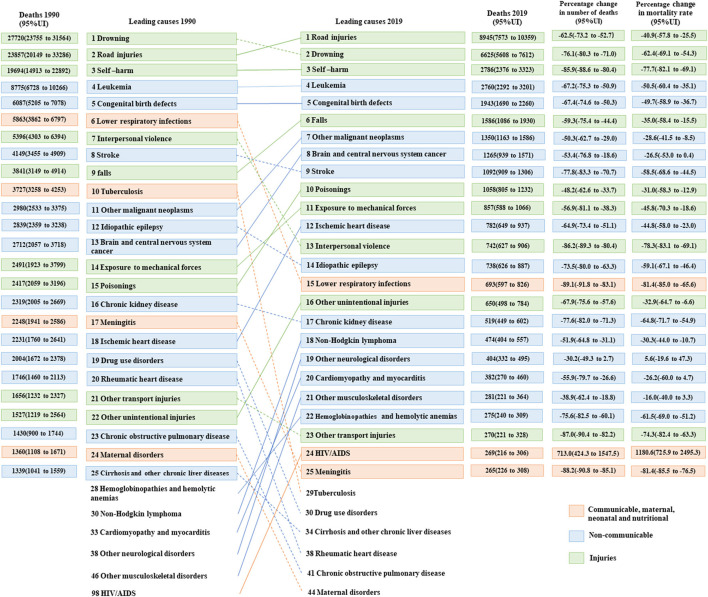
Top 25 causes of death in China, aged 10–19 years, both sexes, 1990 and 2019.

Figure 3 displays the changes in the top 25 causes of death among adolescents from 1990 to 2019. The top five causes of death in 1990 and 2019 were drowning, road injuries, self-harm, leukemia, and congenital birth defects, and road injuries, drowning, self-harm, leukemia, and congenital birth defects, respectively. In addition, this graph reflects a decline in ranking for 13 causes of death, including eight causes of death attributable to NCDs, three from CMNN, and two from injuries. However, there was an increase in ranking for 14 causes of death, including eight causes of death attributable to NCDs, five from injuries, and one from CMNN. Overall, the top five causes of death in both 1990 and 2019 were road injuries, drowning, self-harm, leukemia, and congenital birth defects.

Regarding changes in the leading causes of death among boys and girls in 1990, the top five causes of death in boys were drowning, road injuries, self-harm, leukemia, and interpersonal violence. In contrast, girls' leading five causes of death were self-harm, road injuries, drowning, leukemia, and congenital birth defects. In 2019, the top two causes of death for both boys and girls were road injuries and drowning. In addition, a comparison of death rates due to road injuries and drowning between boys and girls revealed a significant disparity. Boys had nearly three times higher death rates from road injuries (8.7 per 100,000) in comparison to girls (3.2 per 100,000). Similarly, the death rate of drowning among boys (7.0 per 100,000) was found to be over four times higher than that of girls (1.7 per 100,000). The other causes of death in boys were self-harm, leukemia, and falls. However, among girls, the other causes of death were leukemia, self-harm, and congenital birth (Supplementary Figures S5, S6).

**Figure 4:**
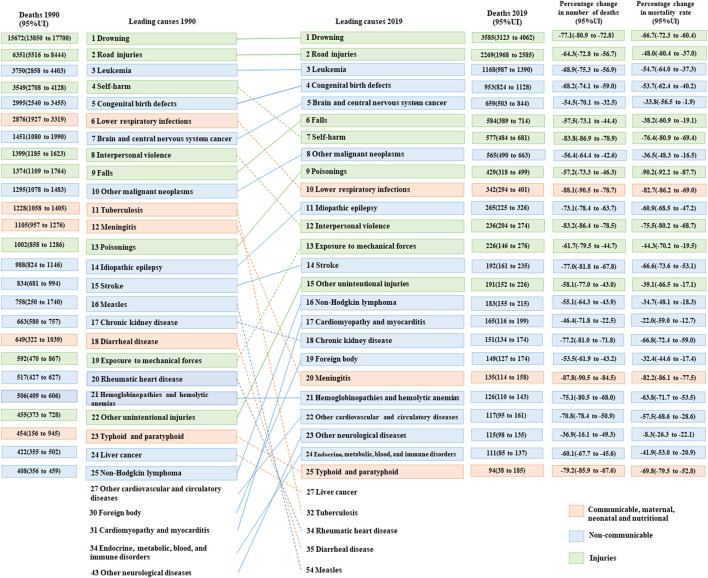
Top 25 causes of death in China, aged 10–14 years, both sexes, 1990 and 2019.

**Figure 5:**
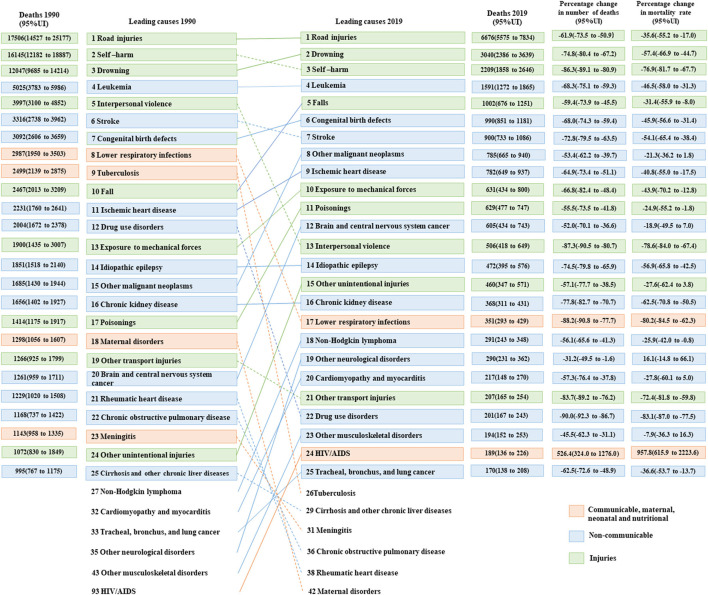
Top 25 causes of death in China, aged 15–19 years, both sexes, 1990 and 2019.

Figures 4, 5 present the changes in the top 25 causes of death among the two age groups of 10–14 years and 15–19 years from 1990 to 2019. In 1990, the top five causes of death among adolescents aged 10–14 years were drowning, road injuries, leukemia, self-harm, and congenital birth defects. By contrast, among adolescents aged 15–19 years, the causes of death were road injuries, self-harm, drowning, leukemia, and interpersonal violence. In 2019, road injuries and drowning were the top two causes of death for the two age groups. The remaining three causes of death among adolescents aged 10–14 years were leukemia, congenital birth defects, and brain and central nervous system cancer. However, for adolescents aged 15–19 years, the leading causes of death were self-harm, leukemia, and falls.

### 3.3. Trends in predicting NCD mortality rates up to 2030

**Figure 6:**
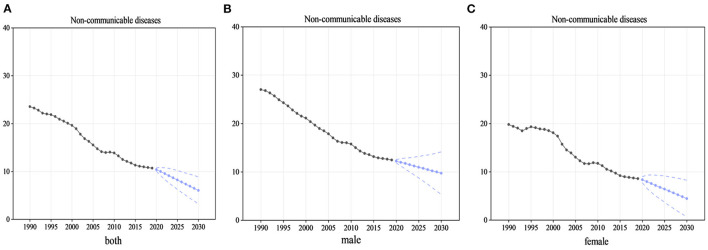
Mortality rates of NCDs for adolescents from 1990 to 2019 and predicted up to 2030. **(A)** The trend and prediction of mortality rates in all adolescents, **(B)** the male adolescents, and **(C)** the female adolescents. In this figure, the black point and solid line denote NCDs mortality rates for adolescents from 1990 to 2019 estimated by GBD 2019. Blue point and solid line denote NCDs mortality rates for adolescents from 2020 to 2030 projected by the Autoregressive Integrated Moving Average model based on the mean rates from 1990 to 2017. Blue dashed lines denote 95% confidence intervals of the projected NCDs mortality rates.

Figures 6A–C shows the trend in mortality rates of NCDs among Chinese adolescents from 1990 to 2019 and the predictions up to 2030. The mortality rates are expected to decline continuously for the next 11 years, reaching 6.0 (95%UI 3.2 to 8.9) in 2030. In 2016, a percentage change of −45.4% from 11.1 (95%UI 10.0 to 12.3), more than one-third, was found. The death rate for girls (percentage change: −50.1%, from 9.0 in 2016 to 4.5 in 2030) was predicted to decrease more than two times as fast as that for boys (percentage change: −24.5%, from 12.9 in 2016 to 9.7 in 2030). Regarding Level 2 categories of death causes, NCDs, except neoplasms, were reduced by more than a third among all adolescents. Meanwhile, for these causes of death among all adolescents, in the period from 2015 to 2030, the mortality of NCDs, except for neoplasms, was predicted to be more than one-third of the causes (Supplementary Tables S9–S11).

### 3.4. Prediction changes in the leading causes of death up to 2030

**Figure 7:**
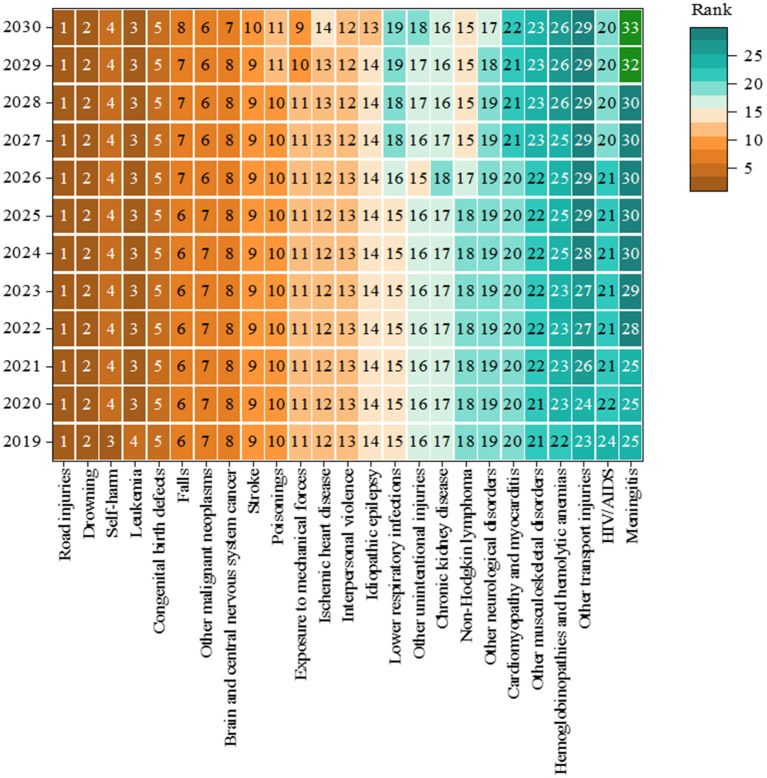
Rank of the top 25 causes of death in China, aged 10–19 years, both sexes from 2019 to 2030.

The trends in the top 25 causes of death between 2019 and 2030 are illustrated in Figure 7. The top five causes of death (road injuries, drowning, self-harm, leukemia, and congenital birth defects) were similar from 2019 to 2030. In addition, it is predicted that hemoglobinopathies and hemolytic anemia (rank 26), other transport injuries (rank 29), and meningitis (rank 33) may drop out of the top 25 causes of death by 2030. Contrastingly, drug use disorders (rank 21), foreign bodies (rank 24), and liver cancer (rank 25) are predicted to be in the top 25 causes of death by 2030. In summary, by 2030, NCDs may account for more than half (15/25) of the top 25 causes of death among adolescents.

In male adolescents, the top five causes of death in 2030 are predicted to be road injuries, drowning, leukemia, self-harm, and congenital birth defects. Furthermore, other transport injuries (rank 27), meningitis (rank 34), and hemoglobinopathies and hemolytic anemia (rank 33) may drop out of the top 25 causes of death in male adolescents by 2030. However, rabies (rank 21), drug use disorders (rank 22), and liver cancer (rank 24) are expected to increase. In female adolescents, the top five causes of death are predicted to be road injuries, leukemia, drowning, congenital birth defects, and other malignant neoplasms. Regarding causes of death, although meningitis (rank 32) and tuberculosis (rank 38) may drop out of the top 25 causes of death, tracheal, bronchial, and lung cancer (rank 23) and drug use disorders (rank 25) will increase in rank for female adolescents (Supplementary Figures S7, S8).

### 3.5. Sensitivity analyses

For the projection of NCDs among adolescents, the ARIMA model's predictive validity test presented a MAPE of 3.31%, and the *P*-value of the Ljung–Box test was >0.05. In four major chronic diseases, the MAPE was all < 10%, and all *P*-values were >0.05. In boys, the MAPE of NCDs was 5.79%, with a *P* > 0.05. However (except for cardiovascular disease), in four major chronic diseases, the MAPE was all < 10%, and the *P* > 0.05. Among girls, the MAPE of NCDs was 3.40%, with a *P* > 0.05. Moreover, the MAPE ranged from 4.12 to 11.37% in four major chronic diseases, and *P* > 0.05 (Supplementary material 3).

For the projection of the 118 causes of death in total adolescents, the MAPE was < 10% for 111 causes of death, with *P* > 0.05. Among girls, the MAPE was < 10% for 115 causes of death, with *P* > 0.05, except for two causes of death. In boys, the MAPE was < 10% for 116 causes of death, with *P* > 0.05 (Supplementary material 4).

## 4. Discussion

This study provides a comprehensive assessment of mortality and causes of death among adolescents in China from 1990 to 2019 and predicts trends in NCD mortality and changes in leading causes of death through 2030. Over the last 29 years, China has had significant success in lowering mortality among teenagers aged 10–19 years, particularly among girls and younger adolescents. Furthermore, compared with different income countries, all-cause mortality rates in China in 2019 were lower than the average level of high-income countries and more than two times as low as in upper-middle-income countries. The mortality rates for Level 1 causes of death declined from 1990 to 2019, with the largest reduction being for CMNN and the smallest for NCDs. Injuries remain a large contributor to adolescent deaths, with road injuries, drowning, and self-harm particularly prominent. Even though the reduction in NCD mortality is the smallest among the three Level 1 categories, it is still possible to achieve the target of reducing premature mortality by one-third between 2016 and 2030.

Before 2019, several studies reported a steady reduction in mortality rates among Chinese adolescents aged over 19 years old, with the exception of a spike in 2008 due to the Wenchuan earthquake (14, 15). Consistent with these studies, downward trends were found in the mortality rates of adolescents aged 10–19; however, when compared to a study using data from the GBD 2016 (14), it was found that the all-cause mortality rates of adolescents in this study were lower than the average level of high-income countries. This finding is consistent with a review that concluded that the Chinese adolescent mortality rate is becoming comparable to that of European countries (24), indicating progress in adolescent mortality reduction over the past 29 years.

In 2016, the HC2030 planning program was introduced, which included a series of special actions focusing on adolescents, such as preventing and controlling four major chronic diseases and promoting physical fitness among students (9). The HC2030 provides an unprecedented opportunity to improve the health and wellbeing of Chinese adolescents (24). In the future, it should be guided by HC2030 to enhance multisectoral cooperation and cross-sectoral actions while addressing emerging health issues among Chinese adolescents.

Despite a general decline in mortality over the past 29 years, this rate of decline in this study varied between the sexes. Addressing inequities in access to health services is significant in promoting adolescent health worldwide and has been the focus of previous programs (7). A previous study found a marked difference regarding sex in adolescent mortality (13). Compared with female adolescents, the mortality rate for male adolescents was higher and declined more slowly. Furthermore, the study also indicates that the mortality rates of injuries (especially road injuries and drowning) were almost six times higher in boys than in girls. A cohort study from Canada found that boys tended to have a higher probability of death, particularly from the age of 14 years, and injuries accounted for 77% of deaths in boys aged 19 years old (25). This difference in this age group may be associated with boys having greater risk-taking behaviors than girls (26). Therefore, gender-specific policies are expected to further lower gender inequalities in mortality among Chinese adolescents.

Promoting adolescent health also requires addressing disparities in access to health services among different age groups. Consistent with a previous study (14), this study found that the mortality rate among adolescents aged 15–19 years shows a downward trend but is slower and higher than that among those aged 10–14 years. Similarly, although some causes of death were similar across age groups, there were differences in the leading causes of death. The findings of this study are similar to those of Dong et al. (15), who found that the top five causes of death among adolescents aged 10–14 years were mainly attributed to NCDs; however, among adolescents aged 15–19 years, the top five causes of death were attributed to injuries. Therefore, it is essential to reorganize priorities and implement age-specific strategies to prevent premature death among Chinese adolescents.

The injuries included in the data are preventable. Efforts to prevent injury-related harm among adolescents will improve their health and wellbeing and promote socioeconomic growth and development (27). This study found that injuries were the main contributors to death among adolescents aged 10–19 years, including unintentional injuries (e.g., road injuries and drowning). The findings of this study are consistent with those of previous studies (14, 15). Globally, injuries, particularly road injuries and drowning, are the leading causes of adolescent death, causing more deaths than CMNN or NCDs (13). Unintentional injuries have received increased recognition and are included in various plans worldwide (28). For road injuries, the WHO has indicated that road safety laws that address key behavioral risk factors (e.g., speeding, drunk driving, and not using helmets, seatbelts, or child car safety equipment) could dramatically reduce deaths (29). Similar legislation, law enforcement, and actions have been implemented in China to reduce road traffic mortality (30, 31).

Consequently, over the years, adolescent deaths due to road injuries have decreased (32). However, the popularity of child safety seats in China has remained low. According to the reported data, the utilization rate of child restraint systems (such as child safety seats) is 2.4% (33). In addition, recent advancements in transportation in China, such as the widespread use of electric bicycles and the emergence of shared bicycle apps, present challenges for current road traffic safety laws and regulations (34). Therefore, it is imperative for the society to take organized actions through legislation, enforcement, and social marketing to improve road safety.

Drowning was the second-most significant contributor to injury mortality among Chinese adolescents aged 10–19 years. According to available data, drowning in natural bodies of water is the main cause of adolescent drowning in countries such as Poland, Cuba, and Japan, while drowning in swimming pools and bathtubs is more common in the United States and Japan (35). However, among Chinese adolescents, recreational swimming and unintentional falls into natural water bodies were the most prominent causes of drowning, particularly in rural areas. Factors such as the inability to swim, exposure to largely unprotected waterways, and lack of supervision contribute to high drowning rates (36). As advocated by the WHO, intersectoral action is necessary to implement effective interventions such as supervision, water skills training, the provision of flotation aids, and fencing (37). Unfortunately, in Chinese laws and regulations, interventions proven to prevent drowning are scarce, and the associated implementation responsibilities are often poorly classified (38). The Chinese government should incorporate these interventions into laws and regulations to address these issues and clearly assign implementation responsibility to a specific department within the national government.

Self-harm was found to be the third leading cause of death among adolescents aged 10–19 years in China, with the highest mortality rate among male adolescents. Compared with a study using data from GBD 2016 (14), the rank of self-harm is rising as a cause of death, illustrating how self-harm is increasingly impacting Chinese adolescents. A 2021 United Nations Children's Fund report indicated that suicide was the fifth most common cause of death for boys and girls aged 10–19 worldwide (39). Depression is the most common mental disorder among people who die by suicide (40). Previous studies suggest that major depressive disorder could increase the risk of suicidal ideation, suicide attempts, and suicide completion among adolescents (41). A study conducted in China using a large-scale sample showed that the prevalence of depressive disorders was 3.0%, with girls having a higher rate than boys, especially for major depressive disorder (42). Poisoning, especially with pesticides, was the most common suicide method in China (43); furthermore, suicide methods, such as charcoal burning and jumping, have increased partly due to the Internet's development, urbanization, and greater use of high-rise buildings (44). Global suicide prevention strategies include a range of components, such as reducing access to common lethal means, responsible reporting by the media, and early identification and management of mental disorders (45). Therefore, China should adopt these strategies and advocate for schools to complement and introduce psychosocial suicide prevention interventions, particularly school-based awareness and skill training interventions that solve both suicidality and underlying psychopathological problems among adolescents.

Other leading causes of death among adolescents aged 10–19 years are preventable or treatable with high-quality healthcare. For example, leukemia mortality remains high in China; however, a study conducted in the United States has shown that leukemia mortality can be reduced using standardized guidelines, consultative support, and expertise sharing (46). Certain congenital birth defects can be avoided or surgically treated (e.g., folic acid supplementation to prevent neural tube defects) (47). Prenatal screening policies and pregnancy termination could partly reduce adolescent deaths due to congenital anomalies (48). Although the leading causes of mortality for both sexes and ages were comparable, differences were found in unintentional injuries, neoplasms, and cardiovascular diseases, highlighting the need for sex- and age-specific measures to prevent premature death. With increasing emphasis on the dangers of NCDs, the 2016 Global Strategy for Women's, Children's, and Adolescents' Health has increased recognition of the crucial role of adolescents in the global targets for universal health coverage. The global strategy includes reducing the premature mortality rate of NCDs by one-third from 2016 to 2030 (3). To achieve this, China released documents and initiatives to prevent and control major chronic diseases and improve adolescent health (8, 9). In our study, the NCD mortality rates, with a reduction of 45.4% from 2016 to 2030, were expected to meet this goal. The four major chronic diseases (excluding neoplasms) are predicted to meet this goal among all adolescents. However, the attainment of this goal varies across the sexes. In 2019, NCDs accounted for approximately half (11/25) of the top 25 causes of death; however, in 2030, NCDs may account for more than half (14/25) of the top 25 causes of death. This indicates that the current Chinese actions and strategies may have achieved the initial results. Nevertheless, the country should introduce targeted strategies to address specific diseases (such as neoplasms) and gender inequality to prevent premature deaths from NCDs.

The common limitations of the GBD data in estimating all-cause and cause-specific mortality have been reported in previous studies (12, 13). This study had some limitations. All results in this report were derived from the estimated data from the GBD study, which is considered reliable and comprehensive; however, the results are limited by the quality of the original data (such as underreporting, the misclassification of causes of death, and garbage codes). Additionally, we could not incorporate the UIs of the GBD data into the ARIMA models when predicting, and only the time series of the estimated means were used in the models. Therefore, the forecast is based solely on past trends in NCD mortality and does not include other factors that may affect future changes.

## 5. Conclusion

There has been a dramatic decline in mortality and a large variation in trends in cause-specific mortality rates among Chinese adolescents aged 10–19 years between 1990 and 2019. The most significant reduction was observed in CMNN, while the least reduction was observed in NCDs. Injuries were major contributors to deaths, with road injuries, drowning, and self-harm being particularly prominent. Although the mortality rate of NCDs declined at a slower rate than that of CMNN and injuries, it is expected to meet the reduction indicators set by the Global Strategy for Women's, Children's, and Adolescents' Health (2016–2030) and HC2030 by 2030. However, the disparity in mortality rates between sexes and age groups persists. Therefore, refocusing health policies on male adolescents and older adolescents, especially regarding road injuries, drowning, and self-harm, is essential. Policies should also focus on NCDs, including congenital birth defects and certain cancers, to ensure that health institutions have a greater capacity to respond. Although progress has been made in reducing mortality over time, further efforts to coordinate actions between governments and stakeholders such as legislators, healthcare professionals, and community members can accelerate the decline.

## Data availability statement

The original contributions presented in the study are included in the article/Supplementary material, further inquiries can be directed to the corresponding author.

## Author contributions

JZ and LN conceptualized and designed the study, completed the statistical analyses, drafted the initial article, and reviewed and revised it. YL contributed to the conceptualization and design of the study, supervised the data collection, the statistical analyses, the initial drafting of the article, and reviewed and revised the article. CZ and JH assisted with the statistical analyses and critically reviewed and revised the article. JZ and LN had full access to all the data in this study and had final responsibility for the decision to submit it for publication. All authors approved the final article as submitted and agreed to be accountable for all study aspects.
