# Supplementary material for: Trends in mortality and causes of death among Chinese adolescents aged 10–19 years from 1990 to 2019
Source: Front Public Health. 2023 Feb 7;11:1075858. doi: 10.3389/fpubh.2023.1075858 (PMC9941149; doi:10.3389/fpubh.2023.1075858)
Supplement: Supplementary file 1 [file Data_Sheet_1.ZIP › supplement-xiu/Supplement 1.docx]

**Supplement 1**

**Table S1.** Death numbers and death rates in 1990 and 2019 in China, ages 10–19 years, both sexes

**Table S2.** Death numbers and death rates in 1990 and 2019 in China, ages 10–19 years, for males

**Table S3.** Death numbers and death rates in 1990 and 2019 in China, ages 10–19 years, for females

**Figure S1.** Percentage of total deaths by level-2 causes in China, aged 10 –19 years, for males from 1990 to 2019

**Figure S2.** Percentage of total deaths by level-2 causes in China, aged 10–19 years, for females from 1990 to 2019

**Figure S3.** Percentage of total deaths by level-2 causes in China, aged 10-14 years from 1990 to 2019

**Figure S4.** Percentage of total deaths by level-2 causes in China, aged 15-19 years from 1990 to 2019

**Figure S5.** Top 25 causes of death in China, aged 10-19 years, for males,1990 and 2019

**Figure S6.** Top 25 causes of death in China, aged 10-19 years, for females,1990 and 2019

**Table S4.** The proportion of level-1 and level-2 causes of death in China, age 10-19 years, both sexes, 1990 and 2019

**Table S5.** The proportion of level-1 and level-2 causes of death in China, age 10-19 years, for males, 1990 and 2019

**Table S6.** The proportion of level-1 and level-2 causes of death in China, age 10-19 years, for females, 1990 and 2019

**Table S7.** The proportion of level-1 and level-2 causes of death in China, age 10-14 years, 1990 and 2019

**Table S8.** The proportion of level-1 and level-2 causes of death in China, age 15-19 years, 1990 and 2019

**Table S9.** Predictions of death rate for non-communicable disease in China, aged 10 – 19 years, both sexes

**Table S10.** Predictions of death rate for non-communicable disease in China, aged 10 – 19 years, for males

**Table S11.** Predictions of death rate for non-communicable disease in China, aged 10 – 19 years, for females

**Figure S7.** Rank of the top 25 causes of death in China, aged 10 – 19 years, for males from 2019 to 2030

**Figure S8.** Rank of the top 25 causes of death in China, aged 10 – 19 years, for females from 2019 to 2030

**Table S1.** Death numbers and death rates in 1990 and 2019 in China, ages 10–19 years, both sexes

| Cause | Number of deaths | | | Mortality rate (per 100,000 people) | | |
| --- | --- | --- | --- | --- | --- | --- |
|  | 1990 | 2019 | Percentage change (%) | 1990 | 2019 | Percentage change (%) |
| All cause | 166703(148841 to 185450) | 41956(36861 to 47242) | -70.0(-70.0 to -80.0) | 72.6(71.8-73.4) | 28.8(27.9-29.7) | -60.0(-50.0 to -70.0) |
| Communicable, maternal, neonatal, and nutritional diseases | 20396 (17175 to 24232) | 2214 (1949 to 2567) | -90.0(-90.0 to -90.0) | 8.9 (8.6 to 9.2) | 1.5 (1.3 to 1.7) | -80.0(-90.0 to -80.0) |
| Enteric infections | 2351(1413 to 3637) | 287(165 to 462) | -87.8(-91.7 to -82.7) | 1.0(0.6 to 1.6) | 0.2(0.1 to 0.3) | -80.8(-86.9 to -72.7) |
| HIV/AIDS and sexually transmitted infections | 74(54 to 95) | 284(230 to 320) | 283.7(195.1 to 431.7) | 0.0(0.0 to 0.0) | 0.2(0.2 to 0.2) | 504.4(364.8 to 737.5) |
| HIV/AIDS | 33(15 to 51) | 269(216 to 306) | 713.0(424.3 to 1547.5) | 0.0(0.0 to 0.0) | 0.2(0.1 to 0.2) | 1180.6(725.9 to 2495.3) |
| Sexually transmitted infections excluding HIV | 41(30 to 50) | 14(13 to 16) | -64.7(-72.5 to -48.6) | 0.0(0.0 to 0.0) | 0.0(0.0 to 0.0) | -44.4(-56.7 to -19.0) |
| Maternal and neonatal disorders | 1360(1108 to 1671) | 77(62 to 93) | -94.3(-95.8 to -92.5) | 0.6(0.5 to 0.7) | 0.1(0.0 to 0.1) | -91.1(-93.4 to -88.3) |
| Maternal disorders | 1360(1108 to 1671) | 77(62 to 93) | -94.3(-95.8 to -92.5) | 0.6(0.5 to 0.7) | 0.1(0 to 0.1) | -91.0(-93.4 to -88.3) |
| Neglected tropical diseases and malaria | 494(220 to 2934) | 47(27 to 60) | -90.4(-98.4 to -78.0) | 0.2(0.1 to 1.3) | 0.0(0.0 to 0.0) | -84.9(-97.5 to -65.3) |
| Chagas disease | 0(0 to 0) | 0(0 to 0) | - | 0.0(0.0 to 0.0) | 0.0(0.0 to 0.0) | - |
| Cystic echinococcosis | 5(3 to 8) | 1(1 to 1) | -81.3(-90.2 to -59.9) | 0.0(0.0 to 0.0) | 0.0(0.0 to 0.0) | -70.6(-84.6 to -36.9) |
| Cysticercosis | 6(0 to 13) | 0(0 to 1) | -91.3(-99.6 to 137.0) | 0.0(0.0 to 0.0) | 0.0(0.0 to 0.0) | -86.3(-99.6 to 273.3) |
| Dengue | 3(1 to 4) | 0(0 to 1) | -89.0(-93.2 to -38.3) | 0.0(0.0 to 0.0) | 0.0(0.0 to 0.0) | -82.7(-89.4to -2.8) |
| Ebola | 0(0 to 0) | 0(0 to 0) | - | 0.0(0.0 to 0.0) | 0.0(0.0 to 0.0) | - |
| Intestinal nematode infections | 22(16 to 30) | 1(1 to 2) | -93.7(-96.2 to -89.5) | 0.0(0.0 to 0.0) | 0.0(0.0 to 0.0) | -90.0(-94.0 to -83.4) |
| Leishmaniasis | 0(0 to 0) | 0(0 to 0) | - | 0.0(0.0 to 0.0) | 0.0(0.0 to 0.0) | - |
| Malaria | 225(0 to 2706) | 0(0 to 0) | -100.0(-100.0 to -100.0 | 0.1(0.0 to 1.2) | 0.0(0.0 to 0.0) | -100.0(-100.0 to -100.0) |
| Other neglected tropical diseases | 37(15 to 49) | 7(4 to 10) | -80.5(-85.9 to -67.6) | 0.0(0.0 to 0.0) | 0.0(0.0 to 0.0) | -69.3(-77.9 to -48.9) |
| Rabies | 160(108 to 332) | 36(14 to 47) | -77.5(-88.8 to -67.1) | 0.1(0.0 to 0.1) | 0.0(0.0 to 0.0) | -64.6(-82.4 to -48.2) |
| Schistosomiasis | 36(23 to 52) | 1(0 to 2) | -97.0(-98.7 to -94.0) | 0.0(0.0 to 0.0) | 0.0(0.0 to 0.0) | -95.3(-98.0 to -90.6) |
| Yellow fever | 0(0 to 0) | 0(0 to 0) | - | 0.0(0.0 to 0.0) | 0.0(0.0 to 0.0) | - |
| Zika virus | 0(0 to 0) | 0(0 to 0) | - | 0.0(0.0 to 0.0) | 0.0(0.0 to 0.0) | - |
| Nutritional deficiencies | 573(495 to 669) | 60(51 to 70) | -89.6(-91.7 to -86.9) | 0.2(0.2 to 0.3) | 0.0(0.0 to 0.0) | -83.6(-87.0 to -79.3) |
| Other infectious diseases | 5664(4476 to 7441) | 539(468 to 639) | -90.5(-92.8 to -87.3) | 2.5(1.9 to 3.2) | 0.4(0.3 to 0.4) | -85.0(-88.7 to -79.9) |
| Acute hepatitis | 591(485 to 710) | 19(14 to 25) | -96.7(-97.9 to -95.0) | 0.3(0.2 to 0.3) | 0.0(0.0 to 0.0) | -94.9(-96.7 to -92.2) |
| Diphtheria | 14(10 to 20) | 1(1 to 1) | -93.6(-95.8 to -90.3) | 0.0(0.0 to 0.0) | 0.0(0.0 to 0.0) | -89.9(-93.3to -84.7) |
| Encephalitis | 549(406 to 652) | 150(125 to 208) | -72.6(-79.5to -54.3) | 0.2(0.2 to 0.3) | 0.1(0.1 to 0.1) | -56.9 (-67.8 to -27.9) |
| Measles | 1102(361 to 2559) | 23(7 to 54) | -97.9(-98.6 to -96.9) | 0.5(0.2 to 1.1) | 0.0(0.0 to 0.0) | -96.7(-97.8to -95.2) |
| Meningitis | 2248(1941 to 2586) | 265(226 to 308) | -88.2(-90.8 to -85.1) | 1.0(0.8 to 1.1) | 0.2(0.2 to 0.2) | -81.4(-85.5 to -76.5) |
| Other unspecified infectious diseases | 319(132 to 424) | 48(38 to 59) | -85.1(-89.3 to -64.0) | 0.1(0.1 to 0.2) | 0.0(0.0 to 0.0) | -76.5(-83.1 to -43.4) |
| Tetanus | 313(161 to 467) | 16(13 to 19) | -95.0(-96.6 to -89.1) | 0.1(0.1 to 0.2) | 0.0(0.0 to 0.0) | -92.1(-94.6 to -82.9) |
| Varicella and herpes zoster | 123(98 to 151) | 8(6 to 10) | -93.5(-95.2 to -91.1) | 0.1(0.0 to 0.1) | 0.0(0.0 to 0.0) | -89.8(-92.4 to -85.9) |
| Whooping cough | 404(29 to 1255) | 9(1 to 28) | -97.7(-99.8 to -71.0) | 0.2(0.0 to 0.5) | 0.0(0.0 to 0.0) | -96.4(-99.7 to -54.3) |
| Respiratory infections and tuberculosis | 9879(7555 to 11276) | 920(795 to 1086) | -90.7(-92.4to -85.8) | 4.3(3.3 to 4.9) | 0.6(0.5 to 0.7) | -85.3(-88.1 to -77.6) |
| Lower respiratory infections | 5863(3862 to 6797) | 693(597 to 826) | -88.2(-90.5 to -78.2) | 2.6(1.7 to 3) | 0.5(0.4 to 0.6) | -81.4(-85.0 to -65.6) |
| Otitis media | 17(8 to 25) | 0(0 to 0) | -98.4(-99.1 to -97.2) | 0.0(0.0 to 0.0) | 0.0(0.0 to 0.0) | -97.5(-98.5 to -95.5) |
| Tuberculosis | 3727(3258 to 4253) | 214(183 to 253) | -94.3(-95.4 to -92.8) | 1.6(1.4 to 1.9) | 0.1(0.1 to 0.2) | -91.0(-92.7 to -88.6) |
| Upper respiratory infections | 272(36 to 443) | 13(8 to 30) | -95.2(-98.1 to -44.1) | 0.1(0.0 to 0.2) | 0.0(0.0 to 0.0) | -92.5(-97.1 to -11.9) |
| Injuries | 92270 (81691 to 103375) | 24181 (21106 to 27415) | -70.0(-70.0 to -80.0) | 40.2 (39.6 to 40.8) | 16.6 (15.9 to 17.3) | -60.0(-50.0 to -70.0) |
| Self-harm and interpersonal violence | 25315(19685 to 29291) | 3548(3046 to 4217) | -86.0(-88.7 to -80.7) | 11.0(8.6 to 12.8) | 2.4(2.1 to 2.9) | -77.9(-82.2 to -69.6) |
| Conflict and terrorism | 4(3 to 5) | 0(0 to 0) | -99.9(-99.9 to- 99.9) | 0.0(0.0 to 0.0) | 0.0(0.0 to 0.0) | 99.9(-99.9 to -99.8) |
| Executions and police conflict | 221(50 to 321) | 20(16 to 27) | -91.0(-94.2 to -42.1) | 0.1(0.0 to 0.1) | 0.0(0.0 to 0.0) | -85.8(-90.9 to -8.7) |
| Interpersonal violence | 5396(4303 to 6394) | 742(627 to 906) | -86.2(-89.3 to -80.4) | 2.3(1.9 to 2.8) | 0.5(0.4 to 0.6) | -78.3(-83.1 to -69.1) |
| Self-harm | 19694(14913 to 22892) | 2786(2376 to 3323) | -85.9(-88.6 to -80.4) | 8.6(6.5 to 10) | 1.9(1.6 to 2.3) | -77.7(-82.1 to -69.1) |
| Transport injuries | 25512(21552 to 35364) | 9214(7792 to 10674) | -63.9(-74.1 to -54.6) | 11.1(9.4 to 15.4) | 6.3(5.3 to 7.3) | -43.1(-59.2 to -28.5) |
| Other unintentional injuries | 1527(1219 to 2564) | 650(498 to 784) | -57.4(-77.6 to -40.7) | 0.7(0.5 to 1.1) | 0.4(0.3 to 0.5) | -76.5(-83.1 to -43.4) |
| Road injuries | 23857(20149 to 33286) | 8945(7573 to 10359) | -62.5(-73.2 to -52.7) | 10.4(8.8 to 14.5) | 6.1(5.2 to 7.1) | -40.9(-57.8 to -25.5) |
| Unintentional injuries | 41443(36436 to 47072) | 11418(9734 to 13124) | -72.4(-77.5 to -66.6) | 18.0(15.9 to 20.5) | 7.8(6.7 to 9) | -56.6(-64.5 to -47.3) |
| Adverse effects of medical treatment | 671(409 to 858) | 84(70 to 112) | -87.4(-91.4 to -77.8) | 0.3(0.2 to 0.4) | 0.1(0.0 to 0.1) | -80.2(-86.5 to -65.0) |
| Animal contact | 542(216 to 662) | 44(37 to 56) | -91.8(-93.9 to -77.0) | 0.2(0.1 to 0.3) | 0.0(0.0 to 0.0) | -87.1(-90.4 to -63.8) |
| Drowning | 27720(23755 to 31564) | 6625(5608 to 7612) | -76.1(-80.4 to -71.0) | 12.1(10.3 to 13.7) | 4.5(3.8 to 5.2) | -62.4(-69.1 to -54.3) |
| Environmental heat and cold exposure | 415(187 to 545) | 41(24 to 53) | -90.0(-92.5 to -84.2) | 0.2(0.1 to 0.2) | 0.0(0.0 to 0.0) | -84.3(-88.3 to -75.0) |
| Exposure to forces of nature | 226(205 to 248) | 25(23 to 28) | -88.9(-88.9 to -88.9) | 0.1(0.1 to 0.1) | 0.0(0.0 to 0.0) | -82.6(-82.6 to -82.6) |
| Exposure to mechanical forces | 2491(1923 to 3799) | 857(588 to 1066) | -65.6(-81.2 to -48.3) | 1.1(0.8 to 1.7) | 0.6(0.4 to 0.7) | -45.8(-70.3 to -18.6) |
| Falls | 3841(3149 to 4914) | 1586(1086 to 1930) | -58.7(-73.6 to -46.4) | 1.7(1.4 to 2.1) | 1.1(0.7 to 1.3) | -35.0(-58.4 to -15.5) |
| Fire, heat, and hot substances | 1028(731 to 1274) | 193(143 to 254) | -81.3(-86.3to -69.3) | 0.4(0.3 to 0.6) | 0.1(0.1 to 0.2) | -70.5(-78.4 to -51.7) |
| Foreign body | 564(495 to 638) | 255(216 to 299) | -54.9(-63.5 to -44.2) | 0.2(0.2 to 0.3) | 0.2(0.1 to 0.2) | -28.9(-42.5 to -12.1) |
| Other transport injuries | 1656(1232 to 2327) | 270(221 to 328) | -83.7(-88.9 to -76.7) | 0.7(0.5 to 1.0) | 0.2(0.2 to 0.2) | -74.3(-82.4 to -63.3) |
| Poisonings | 2417(2059 to 3196) | 1058(805 to 1232) | -56.2(-73.5 to -44.7) | 1.1(0.9 to 1.4) | 0.7(0.6 to 0.8) | -31.0(-58.3 to -12.9) |
| Non-communicable diseases | 54037 (47555 to 60897) | 15561 (13669 to 17582) | -70.0(-70.0 to -80.0) | 23.5 (23.1 to 24.0) | 10.7 (10.2 to 11.2) | -50.0(-50.0 to -60.0) |
| Cardiovascular diseases | 10234(8790 to 11786) | 2717(2300 to 3179) | -73.4(-78.9 to -66.2) | 4.5(3.8 to 5.1) | 1.9(1.6 to 2.2) | -58.2(-66.8 to -46.7) |
| Aortic aneurysm | 42(31 to 56) | 16(13 to 20) | -60.8(-74.3 to -39.4) | 0.0(0.0 to 0.0) | 0.0(0.0 to 0.0) | -38.3(-59.6 to -4.5) |
| Cardiomyopathy and myocarditis | 816(630 to 1273) | 382(270 to 460) | -53.2(-74.6 to -33.5) | 0.4(0.3 to 0.6) | 0.3(0.2 to 0.3) | -26.2(-60.0 to4.7) |
| Endocarditis | 188(84 to 267) | 32(25 to 49) | -82.8(-89.8 to -57.2) | 0.1(0.0 to 0.1) | 0.0(0.0 to 0.0) | -72.9(-83.9 to -32.6) |
| Hypertensive heart disease | 331(187 to 409) | 61(40 to 77) | -81.7(-87.0 to -71.1) | 0.1(0.1 to 0.2) | 0.0(0.0 to 0.1) | -71.1(-79.5 to -54.5) |
| Ischemic heart disease | 2231(1760 to 2641) | 782(649 to 937) | -64.9(-73.4 to -51.1) | 1.0(0.8 to 1.1) | 0.5(0.4 to 0.6) | -44.8(-58.0 to -23.0) |
| Non-rheumatic valvular heart disease | 64(46 to 87) | 16(13 to 20) | -75.2(-83.5 to -62.5) | 0.0(0.0 to 0.0) | 0.0(0.0 to 0.0) | -60.9(-74.0 to -40.9) |
| Other cardiovascular and circulatory diseases | 667(477 to 822) | 198(160 to 279) | -70.3(-79.4 to -45.9) | 0.3(0.2 to 0.4) | 0.1(0.1 to 0.2) | -53.2(-67.5 to -14.7) |
| Rheumatic heart disease | 1746(1460 to 2113) | 137(111 to 168) | -92.1(-94.4 to -89.4) | 0.8(0.6 to 0.9) | 0.1(0.1 to 0.1) | -87.6(-91.2 to -83.4) |
| Stroke | 4149(3455 to 4909) | 1092(909 to 1306) | -73.7(-80.0 to -64.7) | 1.8(1.5 to 2.1) | 0.7(0.6 to 0.9) | -58.5(-68.6to -44.5) |
| Chronic respiratory diseases | 2214(1591 to 2647) | 247(210 to 301) | -88.9(-91.4 to -83.1) | 1.0(0.7 to 1.2) | 0.2(0.1 to 0.2) | -82.4(-86.4 to -73.4) |
| Asthma | 534(361 to 701) | 43(34 to 57) | -92.0(-94.6 to -85.8) | 0.2(0.2 to 0.3) | 0.0(0.0 to 0.0) | -87.4(-91.5 to -77.7) |
| Chronic obstructive pulmonary disease | 1430(900 to 1744) | 117(96 to 150) | -91.8(-94.0 to -85.2) | 0.6(0.4 to 0.8) | 0.1(0.1 to 0.1) | -87.2(-90.5 to -76.7) |
| Interstitial lung disease and pulmonary sarcoidosis | 42(31 to 59) | 15(11 to 22) | -64.6(-74.7 to -49.9) | 0.0(0.0 to 0.0) | 0.0(0.0 to 0.0) | -44.3(-60.1 to -21.1) |
| Other chronic respiratory diseases | 153(103 to 279) | 66(46 to 84) | -56.6(-78.8 to -24.2) | 0.1(0.0 to 0.1) | 0.0(0.0 to 0.1) | -31.6(-66.6 to 19.5) |
| Pneumoconiosis | 55(36 to 77) | 6(5 to 9) | -88.2(-92.5 to -78.7) | 0.0(0.0 to 0.0) | 0.0(0.0 to 0.0) | -81.4(-88.2 to -66.5) |
| Diabetes and kidney diseases | 3409(2970 to 3890) | 720(626 to 825) | -78.9(-82.8 to -73.9) | 1.5(1.3 to 1.7) | 0.5(0.4 to 0.6) | -66.7(-72.8 to -58.9) |
| Acute glomerulonephritis | 628(514 to 780) | 72(55 to 88) | -88.5(-92.3 to -84.9) | 0.3(0.2 to 0.3) | 0.0(0.0 to 0.1) | -81.9(-87.9 to -76.2) |
| Chronic kidney disease | 2319(2005 to 2669) | 519(449 to 602) | -77.6(-82.0 to -71.3) | 1.0(0.9 to 1.2) | 0.4(0.3 to 0.4) | -64.8(-71.7 to -54.9) |
| Diabetes mellitus | 462(400 to 529) | 129(112 to 150) | -72.1(-77.2 to -65.4) | 0.2(0.2 to 0.2) | 0.1(0.1 to 0.1) | -56.1(-64.1 to -45.5) |
| Digestive diseases | 3486(3014 to 3998) | 474(405 to 545) | -86.4(-88.9 to -83.0) | 1.5(1.3 to 1.7) | 0.3(0.3 to 0.4) | -78.6(-82.6 to -73.3) |
| Appendicitis | 299(235 to 380) | 23(18 to 31) | -92.2(-94.6 to -87.5) | 0.1(0.1 to 0.2) | 0.0(0.0 to 0.0) | -87.6(-91.4 to -80.2) |
| Cirrhosis and other chronic liver diseases | 1339(1041 to 1559) | 191(157 to 231) | -85.7(-89.0 to -81.0) | 0.6(0.5 to 0.7) | 0.1(0.1 to 0.2) | -77.5(-82.7 to -70.0) |
| Gallbladder and biliary diseases | 130(68 to 163) | 15(11 to 21) | -88.6(-92.3 to -73.8) | 0.1(0.0 to 0.1) | 0.0(0.0 to 0.0) | -82.1(-87.8 to -58.8) |
| Inflammatory bowel disease | 99(60 to 137) | 18(14 to 23) | -82.0(-87.9 to -67.1) | 0.0(0.0 to 0.1) | 0.0(0.0 to 0.0) | -71.6(-81.0 to -48.2) |
| Inguinal, femoral, and abdominal hernia | 33(19 to 50) | 4(3 to 6) | -87.5(-92.0 to -72.9) | 0.0(0.0 to 0.0) | 0.0(0.0 to 0.0) | -80.3(-87.4 to -57.4) |
| Other digestive diseases | 443(182 to 554) | 46(34 to 60) | -89.7(-93.1 to -77.0) | 0.2(0.1 to 0.2) | 0.0(0.0 to 0.0) | -83.7(-89.1 to -63.8) |
| Pancreatitis | 180(132 to 237) | 41(31 to 50) | -77.5(-5.4 to -4.6) | 0.1(0.1 to 0.1) | 0.0(0.0 to 0.0) | -64.6(-73.6 to -47.1) |
| Paralytic ileus and intestinal obstruction | 377(286 to 454) | 69(55 to 91) | -81.7(-86.2 to -74.9) | 0.2(0.1 to 0.2) | 0.0(0.0 to 0.1) | -71.2(-78.2 to -60.5) |
| Upper digestive system diseases | 575(478 to 713) | 65(53 to 81) | -88.6(-91.2 to -84.8) | 0.3(0.2 to 0.3) | 0.0(0.0 to 0.1) | -82.1(-86.2 to -76.1) |
| Vascular intestinal disorders | 10(8 to 15) | 2(1 to 2) | -81.8(-89.2 to -71.5) | 0.0(0.0 to 0.0) | 0.0(0.0 to 0.0) | -71.3(-83.0 to -55.1) |
| Diarrheal diseases | 1205(607 to 1905) | 67(39 to 115) | -94.5(-96.0 to -91.8) | 0.5(0.3 to 0.8) | 0.0(0.0 to 0.1) | -91.3(-93.7 to -87.1) |
| Invasive Non-typhoidal Salmonella (iNTS) | 144(52 to 308) | 37(12 to 88) | -74.3(-80.6 to -67.7) | 0.1(0.0 to 0.1) | 0.0(0.0 to 0.1) | -59.4(-69.5 to -49.1) |
| Other intestinal infectious diseases | 26(3 to 55) | 4(0 to 8) | -86.5(-99.2 to 6.6) | 0.0(0.0 to 0.0) | 0.0(0.0 to 0.0) | -78.8(-98.8 to 68.0) |
| Typhoid and paratyphoid | 976(336 to 2063) | 179(74 to 350) | -81.6(-87.8 to -71.2) | 0.4(0.1 to 0.9) | 0.1(0.1 to 0.2) | -71.0(-80.7 to -54.6) |
| Maternal and neonatal disorders | 1360(1108 to 1671) | 77(62 to 93) | -94.3(-95.8 to 92.5) | 0.6(0.5 to 0.7) | 0.1(0.0 to 0.1) | -91.1(-93.4 to -88.3) |
| Maternal disorders | 1360(1108 to 1671) | 77(62 to 93) | -94.3(-95.8 to -92.5) | 0.6(0.5 to 0.7) | 0.1(0 to 0.1) | -91.1(-93.4 to -88.3) |
| Mental disorders | 3(2 to 5) | 4(3 to 5) | 35.1(-22.1 to 142.7) | 0.0(0.0 to 0.0) | 0.0(0.0 to 0.0) | 112.8(22.7 to 282.4) |
| Eating disorders | 3(2 to 5) | 4(3 to 5) | 35.1(-22.1 to 142.7) | 0.0(0.0 to 0.0) | 0.0(0.0 to 0.0) | 112.8(22.7 to 282.4) |
| Musculoskeletal disorders | 578(484 to 853) | 297(233 to 380) | -48.7(-62.8 to -37.2) | 0.3(0.2 to 0.4) | 0.2(0.2 to 0.3) | -19.2(-41.4 to -1.0) |
| Other musculoskeletal disorders | 527(438 to 780) | 281(221 to 364) | -46.7(-61.9 to -34.5) | 0.2(0.2 to 0.3) | 0.2(0.2 to 0.2) | -16.0(-40.0 to 3.3) |
| Rheumatoid arthritis | 51(36 to 76) | 16(11 to 20) | -69.4(-78.5 to -57.4) | 0.0(0.0 to 0.0) | 0.0(0.0 to 0.0) | -51.8(-66.1 to -32.8) |
| Neoplasms | 19880(17007 to 22574) | 7028(6108 to 8008) | -64.6(-70.5 to -57.1) | 8.7(7.4 to 9.8) | 4.8(4.2 to 5.5) | -44.3(-53.5 to -32.4) |
| Bladder cancer | 53(44 to 64) | 14(12 to 17) | -73.4(-79.7 to -65.3) | 0.0(0.0 to 0.0) | 0.0(0.0 to 0.0) | -58.2(-68.1 to -45.3) |
| Brain and central nervous system cancer | 2712(2057 to 3718) | 1265(939 to 1571) | -53.3(-70.1 to -36.3) | 1.2(0.9 to 1.6) | 0.9(0.6 to 1.1) | -26.5(-53.0 to 0.4) |
| Cervical cancer | 71(50 to 90) | 23(11 to 30) | -68.2(-82.2 to -51.6) | 0.0(0.0 to 0.0) | 0.0(0.0 to 0.0) | -49.9(-71.9 to -23.7) |
| Colon and rectum cancer | 475(399 to 561) | 178(152 to 209) | -62.5(-70.9 to -52.0) | 0.2(0.2 to 0.2) | 0.1(0.1 to 0.1) | -41.0(-54.2 to -24.5) |
| Hodgkin lymphoma | 340(166 to 461) | 43(35 to 57) | -87.3(-91.5 to -74.3) | 0.1(0.1 to 0.2) | 0.0(0.0 to 0.0) | -80.0(-86.6 to -59.5) |
| Kidney cancer | 98(83 to 113) | 73(63 to 85) | -25.8(-41.9 to -2.7) | 0.0(0.0 to 0.0) | 0.0(0.0 to 0.1) | 16.9(-8.4 to 53.3) |
| Leukemia | 8775(6728 to 10266) | 2760(2292 to 3201) | -68.6(-74.9 to -58.8) | 3.8(2.9 to 4.5) | 1.9(1.6 to 2.2) | -50.5(-60.4 to -35.1) |
| Lip and oral cavity cancer | 70(61 to 80) | 26(23 to 31) | -62.1(-69.5 to -52.5) | 0.0(0.0 to 0.0) | 0.0(0.0 to 0.0) | -40.3(-52.0 to -25.2) |
| Liver cancer | 1239(1057 to 1470) | 215(181 to 260) | -82.6(-86.2 to -77.9) | 0.5(0.5 to 0.6) | 0.1(0.1 to 0.2) | -72.7(-78.3 o -65.2) |
| Malignant skin melanoma | 56(38 to 74) | 24(17 to 29) | -57.7(-70.8 to -42.3) | 0.0(0.0 to 0.0) | 0.0(0.0 to 0.0) | -33.3(-53.9 to -9.1) |
| Nasopharynx cancer | 572(478 to 669) | 75(62 to 89) | -86.9(-89.9 to -82.9) | 0.2(0.2 to 0.3) | 0.1(0.0 to 0.1) | -79.4(-84.1 to -73.0) |
| Non-Hodgkin lymphoma | 1071(928 to 1207) | 474(404 to 557) | -55.7(-64.5 to -43.3) | 0.5(0.4 to 0.5) | 0.3(0.3 to 0.4) | -30.3(-44.0 to -10.7) |
| Other malignant neoplasms | 2980(2533 to 3375) | 1350(1163 to 1586) | -54.7(-62.9 to -41.9) | 1.3(1.1 to 1.5) | 0.9(0.8 to 1.1) | -28.6(-41.5 to -8.5) |
| Other neoplasms | 64(48 to 92) | 38(31 to 52) | -40.4(-59.3 to -15.7) | 0.0(0.0 to 0.0) | 0.0(0.0 to 0.0) | -6.2(-35.9 to 32.9) |
| Ovarian cancer | 136(81 to 182) | 62(45 to 78) | -54.2(-74.0 to -18.9) | 0.1(0.0 to 0.1) | 0.0(0.0 to 0.1) | -27.9(-59.1 to -27.7) |
| Pancreatic cancer | 55(45 to 66) | 30(24 to 37) | -45.0(-58.8 to -24.3) | 0.0(0.0 to 0.0) | 0.0(0.0 to 0.0) | -13.3(-35.1 to 19.3) |
| Stomach cancer | 387(333 to 444) | 97(82 to 113) | -75.0(-79.9 to -68.5) | 0.2(0.1 to 0.2) | 0.1(0.1 to 0.1) | -60.6(-68.3 to -50.4) |
| Testicular cancer | 79(63 to 96) | 36(28 to 44) | -54.9(-67.2 to -38.3) | 0.0(0.0 to 0.0) | 0.0(0.0 to 0.0) | -28.9(-48.4 to -2.8) |
| Thyroid cancer | 61(46 to 70) | 23(19 to 27) | -61.7 (-70.4 to -45.0) | 0.0(0.0 to 0.0) | 0.0(0.0 to 0.0) | -39.6(-53.3 to -13.3) |
| Tracheal, bronchus, and lung cancer | 518(428 to 624) | 197(162 to 238) | -62.0(-71.9 to -49.0) | 0.2(0.2 to 0.3) | 0.1(0.1 to 0.2) | -40.1(-55.8 to -19.6) |
| Neurological disorders | 3599(3016 to 4090) | 1204(1028 to 1439) | -66.6(-73.1 to -56.6) | 1.6(1.3 to 1.8) | 0.8(0.7 to 1.0) | -47.3(-57.6 to -31.6) |
| Idiopathic epilepsy | 2839(2359 to 3238) | 738(626 to 887) | -74.0(-79.1 to -66.0) | 1.2(1 to 1.4) | 0.5(0.4 to 0.6) | -59.1(-67.1 to -46.4) |
| Motor neuron disease | 145(118 to 173) | 58(48 to 70) | -60.1(-69.8 to -45.5) | 0.1(0.1 to 0.1) | 0.0(0.0 to 0.0) | -37.2(-52.5 to -14.1) |
| Multiple sclerosis | 12(5 to 16) | 4(2 to 5) | -68.2(-80.0 to -41.6) | 0.0(0.0 to 0.0) | 0.0(0.0 to 0.0) | -50.0(-68.5 to -8.0) |
| Other neurological disorders | 603(487 to 706) | 404(332 to 495) | -33.0(-49.0 to -6.5) | 0.3(0.2 to 0.3) | 0.3(0.2 to 0.3) | 5.6(-19.6 to 47.3) |
| Other nutritional deficiencies | 56(47 to 75) | 22(18 to 28) | -60.5(-70.5 to -48.8) | 0.0(0.0 to 0.0) | 0.0(0.0 to 0.0) | -37.7(-53.6 to -19.3) |
| Protein-energy malnutrition | 517(435 to 604) | 37(31 to 44) | -92.8(-94.2 to -90.9) | 0.2(0.2 to 0.3) | 0.0(0.0 to 0.0) | -64.6(-82.4 to -48.2) |
| Breast cancer | 69(58 to 83) | 26(22 to 31) | -63.0(-71.7 to -51.7) | 0.0(0.0 to 0.0) | 0.0(0.0 to 0.0) | -41.7(-55.4 to -23.9) |
| Other non-communicable diseases | 8154 (7108 to 9282) | 2519(2209 to 2905) | -69.1(-74.3 to -62.1) | 3.6(3.1 to 4) | 1.7(1.5 to 2) | -51.3(-59.6 to -40.3) |
| Congenital birth defects | 6087(5205 to 7078) | 1943(1690 to 2260) | -68.1(-73.9 to -59.8) | 2.7(2.3 to 3.1) | 1.3(1.2 to 1.5) | -49.7(-58.9 to -36.7) |
| Endocrine, metabolic, blood, and immune disorders | 647(436 to 775) | 242(190 to 297) | -62.6(-70.0 to -48.2) | 0.3(0.2 to 0.3) | 0.2(0.1 to 0.2) | -41.1(-52.8 to -18.4) |
| Gynecological diseases | 3(2 to 11) | 3(1 to 4) | -16.5(-85.4 to105.2) | 0.0(0.0 to 0.0) | 0.0(0.0 to 0.0) | 31.5(-76.9 to 223.3) |
| Hemoglobinopathies and hemolytic anemias | 1126(925 to 1307) | 275(240 to 309) | -75.6(-80.3 to -69.0) | 0.5(0.4 to 0.6) | 0.2(0.2 to 0.2) | -61.5(-69.0 to -51.2) |
| Urinary diseases and male infertility | 291(216 to 344) | 57(49 to 74) | -80.4(-85.1 to -68.4) | 0.1(0.1 to 0.1) | 0.0(0.0 to 0.1) | -69.1(-76.5 to -50.2) |
| Respiratory infections and tuberculosis | 9879(7555 to 11276) | 920(795 to 1086) | -90.7(-92.4 to -85.8) | 4.3(3.3 to 4.9) | 0.6(0.5 to 0.7) | -85.3(-88.1 to -77.6) |
| Lower respiratory infections | 5863(3862 to 6797) | 693(597 to 826) | -88.2(-90.5 to -78.2) | 2.6(1.7 to 3) | 0.5(0.4 to 0.6) | -81.4(-85.0to -65.6) |
| Otitis media | 17(8 to 25) | 0(0 to 0) | -98.4(-99.1 to -97.2) | 0.0(0.0 to 0.0) | 0.0(0.0 to 0.0) | -97.5(-98.5 to -95.5) |
| Tuberculosis | 3727(3258 to 4253) | 214(183 to 253) | -94.3(-95.4 to -92.8) | 1.6(1.4 to 1.9) | 0.1(0.1 to 0.2) | -91.0(-92.7 to -88.6) |
| Upper respiratory infections | 272(36 to 443) | 13(8 to 30) | -95.2(-98.1 to -44.1) | 0.1(0.0 to 0.2) | 0.0(0.0 to 0.0) | -92.5(-97.1 to -11.9) |
| Skin and subcutaneous diseases | 167(90 to 199) | 23(19 to 32) | -86.4(-89.6 to -70.7) | 0.1(0.0 to 0.1) | 0.0(0.0 to 0.0) | -78.6(-83.6 to -53.9) |
| Bacterial skin diseases | 160(82 to 192) | 15(13 to 21) | -90.5(-92.9 to -77.1) | 0.1(0.0 to 0.1) | 0.0(0.0 to 0.0) | -85.1(-88.8 to -63.9) |
| Decubitus ulcer | 2(1 to 6) | 4(2 to 5) | 122.5(-41.9 to 404.4) | 0.0(0.0 to 0.0) | 0.0(0.0 to 0.0) | 250.5(-8.5 to 694.6) |
| Other skin and subcutaneous diseases | 6(4 to 7) | 4(3 to 5) | -32.9(-54.5 to -23.4) | 0.0(0.0 to 0.0) | 0.0(0.0 to 0.0) | -74.3 (-82.4 to -63.3) |
| Substance use disorders | 2313(1955 to 2717) | 329(273 to 392) | -85.8(-88.9 to -81.9) | 1.0(0.9 to 1.2) | 0.2(0.2 to 0.3) | -77.6(-82.6 to -71.5) |
| Alcohol use disorders | 309(258 to 377) | 128(82 to 159) | -58.6(-74.5 to -44.3) | 0.1(0.1 to 0.2) | 0.1(0.1 to 0.1) | -34.8(-59.9 to -12.2) |
| Drug use disorders | 2004(1672 to 2378) | 201(167 to 243) | -90.0(-92.3 to -86.7) | 0.9(0.7 to 1.0) | 0.1(0.1 to 0.2) | -84.2(-87.9 to -79.1) |

**Note: The tables highlighted orange claimed all-cause; The tables highlighted green claimed the level-1 causes of death; The tables highlighted blue claimed the level-2 causes of death.*

**Table S2.** Death numbers and death rates in 1990 and 2019 in China, ages 10–19 years, for males

| Cause | Number of deaths | | | Mortality rate (per 100 000 people) | | |
| --- | --- | --- | --- | --- | --- | --- |
|  | 1990 | 2019 | Percentage change (%) | 1990 | 2019 | Percentage change (%) |
| all cause | 107308(93011 to 121674) | 29406(24735 to 34540) | -72.6(-77.9 to 65.9) | 90.8(78.7 to 102.9) | 37.5(31.5 to 44.0) | -58.7(-66.8 to -48.5) |
| Communicable, maternal, neonatal, and nutritional diseases | 10991 (8532 to 13209) | 1312 (1110 to 1561) | -88.1(-90.7 to -82.8) | 9.3 (7.2 to 11.2) | 1.7 (1.4 to 2.0) | -82.0(-86.0 to -74.1) |
| Enteric infections | 1408(733 to 2201) | 165(92 to 279) | -88.3(-92.5 to -80.6) | 1.2(0.6 to 1.9) | 0.2(0.1 to 0.4) | -82.4(-88.7 to -70.7) |
| HIV/AIDS and sexually transmitted infections | 38(28 to 51) | 169(136 to 190) | 338.4(224.8 to 535.1) | 0.0(0.0 to 0.0) | 0.2(0.2 to 0.2) | 560.8(389.6 to 857.4) |
| HIV/AIDS | 17(8 to 27) | 161(128 to 182) | 825.5(502.6 to 1836.9) | 0.0(0.0 to 0.0) | 0.2(0.2 to 0.2) | 1295.2(502.6 to 1836.9) |
| Sexually transmitted infections excluding HIV | 21(14 to 28) | 8(6 to 9) | -63.5(-74.2 to -40.8) | 0.0(0.0 to 0.0) | 0.0(0.0 to 0.0) | -45.0(-61.1 to -10.8) |
| Maternal and neonatal disorders | - | - | - | - | - | - |
| Maternal disorders | - | - | - | - | - | - |
| Neglected tropical diseases and malaria | 280(113 to 1403) | 34(16 to 46) | -87.9(-97.7 to -74.5) | 0.2(0.1 to 1.2) | 0.0(0.0 to 0.1) | -81.8(-96.5 to -61.6) |
| Chagas disease | - | - | - | 0.0(0.0 to 0.0) | 0.0(0.0 to 0.0) | - |
| Cystic echinococcosis | 3(1 to 5) | 1(0 to 1) | -77.4(-90.3 to -39.4) | 0.0(0.0 to 0.0) | 0.0(0.0 to 0.0) | -65.9(-85.4 to -8.7) |
| Cysticercosis | 3(0 to 9) | 0(0 to 1) | -89.5(-99.8 to 870.4) | 0.0(0.0 to 0.0) | 0.0(0.0 to 0.0) | -84.2(-99.8 to 1362.9) |
| Dengue | 2(0 to 3) | 0(0 to 1) | -89.9(-94.5 to -25.5) | 0.0(0.0 to 0.0) | 0.0(0.0 to 0.0) | -84.8(-91.8 to 12.3) |
| Ebola | - | - | - | - | - | - |
| Intestinal nematode infections | 14(9 to 19) | 1(1 to 2) | -92.4(-96.1 to -86.0) | 0.0(0.0 to 0.0) | 0.0(0.0 to 0.0) | -88.6 (-94.1to -78.8) |
| Leishmaniasis | 0(0 to 0) | 0(0 to 0) | - | 0.0(0.0 to 0.0) | 0.0(0.0 to 0.0) | - |
| Malaria | 100(0 to 1212) | 0(0 to 0) | -100.0(-100.0 to -100.0 | 0.1(0.0 to 1.0) | 0.0(0.0 to 0.0) | -100.0(-100.0 to -100.0) |
| Other neglected tropical diseases | 25(8 to 35) | 5(2 to 8) | -79.7(-86.6 to -59.3) | 0.0(0.0 to 0.0) | 0.0(0.0 to 0.0) | -69.5(-79.7 to -38.7) |
| Rabies | 113(46 to 189) | 26(7 to 36) | -77.1(-85.7 to -65.3) | 0.1(0.0 to 0.2) | 0.0(0.0 to 0.0) | -65.5(-78.5 to -47.7) |
| Schistosomiasis | 20(11 to 32) | 1(0 to 2) | -96.3(-98.8 to -90.8) | 0.0(0.0 to 0.0) | 0.0(0.0 to 0.0) | -94.4(-98.1 to -86.1) |
| Yellow fever | 0(0 to 0) | 0(0 to 0) | - | - | - | - |
| Zika virus | 0(0 to 0) | 0(0 to 0) | - | - | - | - |
| Nutritional deficiencies | 342(275 to 414) | 41(34 to 50) | -87.9(-90.9 to -83.4) | 0.3(0.2 to 0.4) | 0.1(0.0 to 0.1) | -81.7(-86.2 to -74.9) |
| Other infectious diseases | 3213(2450 to 4175) | 319(261 to 393) | -90.1(-92.8 to -85.9) | 2.7(2.1 to 3.5) | 0.4(0.3 to 0.5) | -85.1(-89.1 to -78.7) |
| Acute hepatitis | 332(255 to 424) | 12(8 to 17) | -96.3(-98.0 to -93.8) | 0.3(0.2 to 0.4) | 0.0(0.0 to 0.0) | -94.5(-97.0 to -90.7) |
| Diphtheria | 9(6 to 13) | 1(0 to 1) | -92.9(-95.8 to -88.0) | 0.0(0.0 to 0.0) | 0.0(0.0 to 0.0) | -89.3(-93.6 to -81.9) |
| Encephalitis | 314(201 to 381) | 85(67to 129) | -72.9(-80.8 to -46.7) | 0.3(0.2 to 0.3) | 0.1(0.1 to 0.2) | -59.1 (-71.0 to -19.7) |
| Measles | 527(173 to 1228) | 12(4 to 28) | -97.8(-98.6 to -96.6) | 0.3(0.2 to 0.3) | 0.1(0.1 to 0.2) | -96.6(-97.8 to -94.8) |
| Meningitis | 1337(1085 to 1578) | 163(129 to 201) | -87.8(-91.2 to -83.4) | 1.1(0.9 to 1.3) | 0.2(0.2 to 0.3) | -81.7(-86.7 to -74.9) |
| Other unspecified infectious diseases | 222(70 to 308) | 28(21 to 37) | -87.3(-91.5 to -61.4) | 0.2(0.1 to 0.3) | 0.0(0.0 to 0.0) | -80.9(-87.2 to -41.8) |
| Tetanus | 215(73 to 348) | 9(7 to 11) | -95.8(-97.4 to -86.2) | 0.2(0.1 to 0.3) | 0.0(0.0 to 0.0) | -93.7(-96.0 to -79.3) |
| Varicella and herpes zoster | 64(46 to 86) | 4(3 to 6) | -93.1(-95.6 to -89.2) | 0.1(0.0 to 0.1) | 0.0(0.0 to 0.0) | -89.6(-93.4 to -83.7) |
| Whooping cough | 193(14 to 596) | 5(0 to 14) | -97.6(-99.8 to -69.4) | 0.2(0.0 to 0.5) | 0.0(0.0 to 0.0) | -96.3(-99.7 to -53.8) |
| Respiratory infections and tuberculosis | 5709(4113 to 6706) | 584(478 to 723) | -89.8(-92.2 to -82.4) | 4.8(3.5 to 5.7) | 0.7(0.6 to 0.9) | -84.6(-88.2 to -73.5) |
| Lower respiratory infections | 3624(2230 to 4299) | 449(363 to 556) | -87.6(-90.8 to -73.8) | 3.1(1.9 to 3.6) | 0.6(0.5 to 0.7) | -81.3(-86.1 to -60.5) |
| Otitis media | 10(6 to 17) | 0(0 to 0) | -99.3(-99.7 to -98.1) | 0.0(0.0 to 0.0) | 0.0(0.0 to 0.0) | -99.0(-99.5 to -97.2) |
| Tuberculosis | 1888(1533 to 2206) | 125(101 to 155) | -93.4(-95.0 to -91.2) | 1.6(1.3 to 1.9) | 0.2(0.1 to 0.2) | -90.0(-92.4 to -86.7) |
| Upper respiratory infections | 186(310 to 28) | 10(6 to 23) | -94.6(-97.9 to -44.7) | 0.2(0.0 to 0.3) | 0.0(0.0 to 0.0) | -91.8(-96.8 to -16.6) |
| Injuries | 64353 (55483 to 74367) | 18326 (15292 to 21605) | -71.5(-77.2 to -64.3) | 54.4 (46.9 to 62.9) | 23.3 (19.5 to 27.6) | -57.1(-65.7 to -46.2) |
| Self-harm and interpersonal violence | 13697(8549 to 16720) | 2294(1835 to 2933) | -83.2(-87.6 to -72.0) | 11.6(7.2 to 14.1) | 2.9(2.3 to 3.7) | -74.7(-81.3 to -57.7) |
| Conflict and terrorism | 3(2 to 4) | 0(0 to 0) | -99.9(-99.9 to- 99.9) | 0.0(0.0 to 0.0) | 0.0(0.0 to 0.0) | 99.9(-99.9 to -99.8) |
| Executions and police conflict | 208(38 to 308) | 14(10 to 20) | -93.5(-96.1 to -42.2) | 0.2(0.0 to 0.3) | 0.0(0.0 to 0.0) | -90.2(-94.2 to -12.9) |
| Interpersonal violence | 4149(3131 to 5079) | 528(420 to 682) | -87.3(-90.7 to -79.9) | 3.5(2.6 to 4.3) | 0.7(0.5 to 0.9) | -87.3(-90.7 to -79.9) |
| Self-harm | 9337 (4911 to 11625) | 1753(1360 to 2302) | -81.2(-86.3 to -67.3) | 7.9(4.2 to 9.8) | 2.2(1.7 to 2.9) | -71.7(-79.3 to -50.7) |
| Transport injuries | 18917(15268 to 28899) | 7022(5705 to 8437) | -62.9(-75.8 to -50.3) | 16.0(12.9 to 24.4) | 9.0(7.3 to 10.8) | -44.0(-63.6 to -25.1) |
| Other unintentional injuries | 1268(978 to 2268) | 567(409 to 699) | -55.3(-78.9 to -34.7) | 1.1(0.8 to 1.9) | 0.7(0.5 to 0.9) | -32.6(-68.2 to -1.5) |
| Road injuries | 17659(14244 to 26910) | 6804(5530 to 8160) | -61.5(-75.1 to -48.2) | 14.9(12.0 to 22.8) | 8.7(7.1 to 10.4) | -41.9(-62.4 to -21.9) |
| Unintentional injuries | 31739(27161 to 36596) | 9010(7387 to 10673) | -71.6(-77.4 to -64.1) | 26.8(23.0 to 30.1) | 11.5(9.4 to 13.6) | -57.2(-65.9 to -45.8) |
| Adverse effects of medical treatment | 331(188 to 446) | 48(37 to 68) | -85.5(-90.8 to -73.1) | 0.3(0.2 to 0.4) | 0.1(0.0 to 0.1) | -78.1(-86.1 to -59.5) |
| Animal contact | 392(137 to 505) | 29(22 to 39) | -92.6(-95.3 to -73.8) | 0.3(0.1 to 0.4) | 0.0(0.0 to 0.0) | -88.8(-92.8 to -60.5) |
| Drowning | 21582(17813 to 25129) | 5501(4442 to 6489) | -74.5(-79.7 to -67.9) | 18.3(15.1 to 21.3) | 7.0(5.7 to 8.3) | -61.6(-69.3 to -51.6) |
| Environmental heat and cold exposure | 345(131 to 466) | 30(17 to 44) | -91.3(-94.0 to -84.0) | 0.3(0.1 to 0.4) | 0.0(0.0 to 0.1) | -86.9(-90.9 to -75.8) |
| Exposure to forces of nature | 148(134 to 162) | 17(15 to 18) | -88.7(-88.7 to -88.7) | 0.1(0.1 to 0.1) | 0.0(0.0 to 0.0) | -82.9(-82.9 to -82.9) |
| Exposure to mechanical forces | 2120(1564 to 3422) | 697(461 to 892) | -67.1(-83.9 to -48.2) | 1.8(1.3 to 2.9) | 0.9(0.6 to 1.1) | -50.4 (-75.7 to -21.9) |
| Falls | 3015(2338 to 3966) | 1266(801 to 1556) | -59.3(-75.4 to -44.4) | 2.6(2.0 to 3.4) | 1.61.0 to 2.0) | -38.7(-63.0 to -16.1) |
| Fire, heat, and hot substances | 656(409 to 816) | 121(89 to 164) | -81.6(-86.4 to -72.7) | 0.3(0.6 to 0.7) | 0.2(0.1 to 0.2) | -72.3(-79.5 to -58.8) |
| Foreign body | 375(314 to 436) | 187(149 to 228) | -50.2(-62.5 to -33.2) | 0.3(0.3 to 0.4) | 0.2(0.2 to 0.3) | -24.9(-43.5 to 0.8) |
| Other transport injuries | 1259(832 to 1936) | 218(173 to 275) | -82.7(-89.3 to -72.0) | 1.1(0.7 to 1.6) | 0.3(0.2 to 0.4) | -73.9(-83.8 to -57.8) |
| Poisonings | 1507(1218 to 2071) | 587(361 to 733) | -61.1(-80.2 to -46.2) | 1.3(1.0 to 1.8) | 0.7(0.5 to 0.9) | -41.3(-70.2 to -18.9) |
| Non-communicable diseases | 31964 (26422 to 36898) | 9768 (8110 to 11650) | -69.4 (-75.9 to -60.9) | 27.0 (22.4 to 31.2) | 12.5 (10.3 to 14.9) | -53.9(-63.6 to -41.0) |
| Cardiovascular diseases | 6457(5103 to 7708) | 1889(1493 to 2310) | -70.7(-78.5 to -59.3) | 5.5(4.3 to 6.5) | 2.4(1.9 to 2.9) | -55.9(-67.5 to -38.6) |
| Aortic aneurysm | 32(21 to 45) | 12(9 to 16) | -61.6(-77.7 to -31.9) | 0.0(0.0 to 0.0) | 0.0(0.0 to 0.0) | -42.1(-66.4 to 2.7) |
| Cardiomyopathy and myocarditis | 506(383 to 803) | 246(165 to 320) | -51.5(-71.8 to -30.2) | 0.4(0.3 to 0.7) | 0.3(0.2 to 0.4) | -26.8(-57.4 to 5.3) |
| Endocarditis | 116(46 to 174) | 20(12 to 34) | -83.1(-91.0 to -51.8) | 0.1(0.0 to 0.1) | 0.0(0.0 to 0.0) | -74.5(-86.4 to -27.3) |
| Hypertensive heart disease | 209(95 to 278) | 42(26 to 56) | -80.0(-87.1 to -62.5) | 0.2(0.1 to 0.2) | 0.1(0.0 to 0.1) | -69.8(-80.5 to -43.4) |
| Ischemic heart disease | 1517(1119 to 1862) | 575(452 to 718) | -62.1(-73.0 to -43.0) | 1.3(0.9 to 1.6) | 0.7(0.6 to 0.9) | -42.9(-59.2 to -14.1) |
| Non-rheumatic valvular heart disease | 35(23 to 50) | 9(7 to 13) | -74.2(-84.1 to -48.8) | 0.0(0.0 to 0.0) | 0.0(0.0 to 0.0) | -86.6(-90.8 to -77.6) |
| Other cardiovascular and circulatory diseases | 384(251 to 504) | 115(87 to 167) | -70.2(-81.0 to -41.2) | 0.3(0.2 to 0.4) | 0.1(0.1 to 0.2) | -55.1(-71.3 to -11.4) |
| Rheumatic heart disease | 876(669 to 1097) | 83(62 to 111) | -90.5(-94.1 to -85.6) | 0.7(0.6 to 0.9) | 0.1(0.1 to 0.1) | -85.7(-91.1 to -78.3) |
| Stroke | 2781(2161 to 3401) | 788(613 to 989) | -71.7(-79.9 to -59.0) | 2.4(1.8 to 2.9) | 1.0 (0.8 to 1.3) | -57.3(-69.7 to -38.2) |
| Chronic respiratory diseases | 1260(871 to 1548) | 153(122 to 194) | -87.8(-91.1 to -81.5) | 1.1(0.7 to 1.3) | 0.2(0.2 to 0.2) | -81.7(-86.6 to -72.0) |
| Asthma | 279(174 to 382) | 25(19 to 35) | -91.1(-94.5 to -82.8) | 0.2(0.1 to 0.3) | 0.0(0.0 to 0.0) | -86.6(-91.7 to -74.1) |
| Chronic obstructive pulmonary disease | 825(543 to 1030) | 73(56 to 95) | -91.1(-93.9 to -85.2) | 0.5(0.7 to 0.9) | 0.1(0.1 to 0.1) | -86.6(-90.8 to -77.6) |
| Interstitial lung disease and pulmonary sarcoidosis | 24(15 to 36) | 8(5 to 12) | -66.4(-79.6 to -45.2) | 0.0(0.0 to 0.0) | 0.0(0.0 to 0.0) | -49.3(-69.3 to -17.4) |
| Other chronic respiratory diseases | 94(62 to 171) | 43(27 to 58) | -54.6(-76.4 to -15.0) | 0.1(0.1 to 0.1) | 0.1(0.0 to 0.1) | -31.5(-64.4 to 28.2) |
| Pneumoconiosis | 39(23 to 56) | 4(3 to 6) | -88.5(-93.5 to -75.9) | 0.0(0.0 to 0.0) | 0.0(0.0 to 0.0) | -82.6(-90.2 to -63.6) |
| Diabetes and kidney diseases | 1899(1546 to 2265) | 415(336 to 508) | -78.1(-83.6 to -70.4) | 1.6(1.3 to 1.9) | 0.5(0.4 to 0.6) | -67.0(-75.3 to -55.4) |
| Acute glomerulonephritis | 376(284 to 512) | 43(28 to 56) | -88.6(-93.5 to -83.2) | 0.3(0.2 to 0.4) | 0.0(0.0 to 0.1) | -82.7(-90.2 to -74.7) |
| Chronic kidney disease | 1323(1044 to 1595) | 306(247 to 377) | -76.9(-83.0 to -66.8) | 1.1(0.9 to 1.3) | 0.4(0.3 to 0.5) | -65.1(-74.3 to -50.0) |
| Diabetes mellitus | 200(157 to 239) | 67(54 to 82) | -66.6(-75.1 to -52.5) | 0.2(0.1 to 0.2) | 0.1(0.1 to 0.1) | -49.7(-62.4 to -28.4) |
| Digestive diseases | 2006(1617 to 2361) | 297(240 to 364) | -85.2(-88.7 to -79.9) | 1.7(1.4 to 2.0) | 0.4(0.3 to 0.5) | -77.7(-82.9 to -69.7) |
| Appendicitis | 162(113 to 209) | 13(9 to 19) | -92.2(-94.9 to -84.9) | 0.1(0.1 to 0.2) | 0.0(0.0 to 0.0) | -88.3(-92.2 to -77.3) |
| Cirrhosis and other chronic liver diseases | 766(533 to 929) | 120 (92 to 154) | -84.3(-88.8 to -76.6) | 0.6(0.5 to 0.8) | 0.2(0.1 to 0.2) | -76.3(-83.2 to -64.7) |
| Gallbladder and biliary diseases | 72(34 to 94) | 9(7 to 14) | -86.8(-91.6 to -68.7) | 0.1(0.0 to 0.1) | 0.0(0.0 to 0.0) | -80.2(-87.3 to -52.7) |
| Inflammatory bowel disease | 49(22 to 77) | 11(8 to 15) | -77.6(-86.9 to -51.3) | 0.0(0.0 to 0.1) | 0.0(0.0 to 0.0) | -66.2 (-80.2 to -26.6) |
| Inguinal, femoral, and abdominal hernia | 27(14 to 44) | 3(2 to 4) | -88.4(-93.3 to -74.0) | 0.0(0.0 to 0.0) | 0.0(0.0 to 0.0) | -82.6(-89.8 to -60.7) |
| Other digestive diseases | 262(119 to 332) | 30(21 to 42) | -88.6(-92.6 to -78.3) | 0.2(0.1 to 0.3) | 0.0(0.0 to 0.1) | -82.8(-88.9 to -67.3) |
| Pancreatitis | 85(61 to 124) | 22(16 to 30) | -73.9(-82.3 to -61.1) | 0.1(0.1 to 0.1) | 0.0(0.0 to 0.0) | -60.7(-73.3 to -41.4) |
| Paralytic ileus and intestinal obstruction | 234(163 to 293) | 46(33 to 65) | -80.4(-85.8 to -71.1) | 0.2(0.1 to 0.2) | 0.0(0.0 to 0.1) | -70.5(-78.5 to -56.4) |
| Upper digestive system diseases | 344(272 to 422) | 42(32 to 56) | -87.8(-91.3 to -82.6) | 0.3(0.2 to 0.4) | 0.0(0.0 to 0.1) | -81.7 (-86.9 to -73.8) |
| Vascular intestinal disorders | 5(4 to 7) | 1(1 to 1) | -78.9(-85.5 to -66.4) | 0.0(0.0 to 0.0) | 0.0(0.0 to 0.0) | -68.1(-78.1 to -49.4) |
| Diarrheal diseases | 730(254 to 1025) | 39(20 to 76) | -94.7(-96.4 to -89.0) | 0.6(0.2 to 0.9) | 0.0(0.0 to 0.1) | -92.0(-94.6 to -83.5) |
| Invasive Non-typhoidal Salmonella (iNTS) | 78(27 to 170) | 20(6 to 49) | -74.3(-81.5 to -65.3) | 0.1(0.0 to 0.1) | 0.0(0.0 to 0.1) | -61.3(-72.1 to -47.8) |
| Other intestinal infectious diseases | 14(1 to 36) | 2(0 to 6) | -84.9(-99.8 to 417.1) | 0.0(0.0 to 0.0) | 0.0(0.0 to 0.0) | -77.2(-99.7 to 679.6) |
| Typhoid and paratyphoid | 586(201 to 1275) | 104(42 to 205) | -82.3(-88.9 to -71.2) | 0.5(0.2 to 1.1) | 0.1(0.1 to 0.3) | -73.3(-83.2 to -56.6) |
| Maternal and neonatal disorders | 1360(1108 to 1671) | 77(62 to 93) | - | 0.6(0.5 to 0.7) | 0.1(0.0 to 0.1) | - |
| Maternal disorders | - | - | - | - | - | - |
| Mental disorders | 0(0 to 0) | 1(1 to 1) | 479.5(96.1 to 1565.5) | 0.0(0.0 to 0.0) | 0.0(0.0 to 0.0) | 773.6(195.7 to 2410.7) |
| Eating disorders | 0(0 to 0) | 1(1to 1) | 479.5(96.1 to 1565.5) | 0.0(0.0 to 0.0) | 0.0(0.0 to 0.0) | 773.6(195.7 to 2410.7) |
| Musculoskeletal disorders | 184(150 to 217) | 67(54 to 82) | -63.7(-71.9 to -51.4) | 0.2(0.1 to 0.2) | 0.1(0.1 to 0.1) | -45.2(-57.7 to -26.7) |
| Other musculoskeletal disorders | 163(133 to 193) | 59(47 to 73) | -64.0(-72.3 to -51.4) | 0.1(0.1 to 0.2) | 0.1(0.1 to 0.1) | -45.7(-58.3 to -26.7) |
| Rheumatoid arthritis | 20(15 to 27) | 8(6 to 11) | -60.9(-73.3 to -41.1) | 0.0(0.0 to 0.0) | 0.0(0.0 to 0.0) | -41.0(-59.7 to -11.2) |
| Neoplasms | 11771(9305 to13881) | 4312(3510 to 5187) | -63.4(-71.4 to -51.7) | 10.0(7.9 to 11.8) | 5.5(4.5 to 6.6) | -44.8(-56.9 to -27.1) |
| Bladder cancer | 31(24 to 41) | 9 (7 to 12) | -71.4(-80.2 to -58.1) | 0.0(0.0 to 0.0) | 0.0(0.0 to 0.0) | -56.9(-70.1 to -36.8) |
| Brain and central nervous system cancer | 1631(1062 to 2446) | 762(480 to 1056) | -53.3(-71.8 to -26.1) | 1.4(0.9 to 2.1) | 1.0(0.6 to 1.3) | -29.6(-57.5 to 11.4) |
| Cervical cancer | - | - | - | - | - | - |
| Colon and rectum cancer | 286(225 to 358) | 115(90 to 144) | -59.8(-72.5 to -43.6) | 0.2(0.2 to 0.3) | 0.1(0.1 to 0.2) | -39.5(-58.6 to -15.0) |
| Hodgkin lymphoma | 217(100 to 298) | 27(20 to 40) | -87.4(-91.9 to -73.2) | 0.2(0.1 to 0.3) | 0.0(0.0 to 0.1) | -81.0(-87.7 to -59.6) |
| Kidney cancer | 52(41 to 64) | 44(34 to 55) | -16.3(-40.9 to -23.2) | 0.0(0.0 to 0.1) | 0.1(0.0 to 0.1) | 26.2(-10.9 to 85.8) |
| Leukemia | 5236(3453 to 6384) | 1718(1274 to 2121) | -67.2(-75.3 to -50.9) | 4.4(2.9 to 5.4) | 2.2(1.6 to 2.7) | -50.5(-62.8 to -26.0) |
| Lip and oral cavity cancer | 30(23 to 37) | 15(11 to 18) | -51.3(-67.2 to -29.6) | 0.0(0.0 to 0.0) | 0.0(0.0 to 0.0) | -26.6(-50.6 to 6.2) |
| Liver cancer | 842(684 to 1044) | 149(118 to 191) | -82.4(-86.9 to -74.8) | 0.7(0.6 to 0.9) | 0.2(0.1 to 0.2) | -73.4(-80.3 o -62.0) |
| Malignant skin melanoma | 31(20 to 40) | 13(9 to 16) | -59.6(-69.8 to -43.0) | 0.0(0.0 to 0.0) | 0.0(0.0 to 0.0) | -39.1(-54.4 to -14.1) |
| Nasopharynx cancer | 395(315 to 483) | 54(42 to 68) | -86.3(-90.1 to -81.1) | 0.3(0.3 to 0.4) | 0.1(0.1 to 0.1) | -79.3(-85.0 to -71.5) |
| Non-Hodgkin lymphoma | 651(516 to 768) | 313(247 to 390) | -51.9(-64.8 to -31.1) | 0.6(0.4 to 0.6) | 0.4(0.3 to 0.5) | -27.5 (-46.9 to 3.9) |
| Other malignant neoplasms | 1633(1260 to 1938) | 812(655 to 1019) | -50.3(-62.7 to -29.0) | 1.4(1.1 to 1.6) | 1.0(0.8 to 1.3) | -25.1(-43.8 to 7.0) |
| Other neoplasms | 35(19 to 51) | 22(16 to 36) | -37.0(-59.5 to 2.8) | 0.0(0.0 to 0.0) | 0.0(0.0 to 0.0) | -5.1(-38.9 to 55.0) |
| Ovarian cancer | - | - | - | - | - | - |
| Pancreatic cancer | 36(27 to 46) | 21(16 to 28) | -39.8(-60.2to -6.4) | 0.0(0.0 to 0.0) | 0.0(0.0 to 0.0) | -9.2(-40.0 to -41.1) |
| Stomach cancer | 215(171 to 262) | 61(48 to 76) | -71.7(-79.0 to -60.2) | 0.2(0.1 to 0.2) | 0.1(0.1 to 0.1) | -57.4(-68.4 to -40.1) |
| Testicular cancer | 79(63 to 96) | 36(28 to 44) | -54.9(-67.2 to -38.3) | 0.1(0.1 to 0.1) | 0.0(0.0 to 0.1) | -16.3(-40.9 to 23.2) |
| Thyroid cancer | 26(21 to 32) | 15(11 to 19) | -43.4 (-60.7 to -22.1) | 0.0(0.0 to 0.0) | 0.0(0.0 to 0.0) | -14.7(-40.8 to 17.4) |
| Tracheal, bronchus, and lung cancer | 335(260 to 421) | 123(93 to 159) | -63.3(-76.6 to -45.4) | 0.3(0.2 to 0.4) | 0.2(0.1 to 0.2) | -44.7(-64.7 to -17.7) |
| Neurological disorders | 2455(1922 to 2864) | 883(720 to 1092) | -64.0(-72.6 to -49.8) | 2.1(1.6 to 2.4) | 1.1(0.9 to 1.4) | -45.8(-58.8 to -24.3) |
| Idiopathic epilepsy | 1830(1405 to 2148) | 484(391 to 609) | -73.5(-80.0 to -63.3) | 1.5(1.2 to 1.8) | 0.6(0.5 to 0.8) | -60.1(-69.9 to -44.7) |
| Motor neuron disease | 123(96 to 151) | 50(40 to 62) | -59.3(-70.7 to -42.2) | 0.1(0.1 to 0.1) | 0.1(0.1 to 0.1) | -38.7(-55.9 to -12.9) |
| Multiple sclerosis | 3(1 to 5) | 0(0 to 1)+ | -85.0(-92.0 to -32.0) | 0.0(0.0 to 0.0) | 0.0(0.0 to 0.0) | -77.4(-88.0 to -2.5) |
| Other neurological disorders | 499(388 to 593) | 348(276 to 440) | -30.2(-49.3 to 2.7) | 0.4(0.3 to 0.5) | 0.4(0.4 to 0.6) | 5.3(-23.5 to 54.8) |
| Other nutritional deficiencies | 30(21 to 45) | 14(11 to 19) | -51.6(-67.7 to -28.1) | 0.0(0.0 to 0.0) | 0.0(0.0 to 0.0) | -27.1 (-51.2 to -8.3) |
| Protein-energy malnutrition | 313(244 to 378) | 27(21 to 33) | -91.3(-93.4 to -88.2) | 0.3(0.2 to 0.3) | 0.0(0.0 to 0.0) | -86.9(-90.1 to -82.2) |
| Breast cancer | 8(6 to 9) | 4(3 to 5) | -46.7(-59.4 to -26.9) | 0.0(0.0 to 0.0) | 0.0(0.0 to 0.0) | -19.6(-38.8 to -10.1) |
| Other non-communicable diseases | 4672 (3723 to 5385) | 1497(1232 to 1812) | -68.0(-74.7 to -54.5) | 4.0(3.1 to 4.1) | 1.9(1.6 to 2.0) | -51.7(-61.9 to -31.4) |
| Congenital birth defects | 3610(2736 to 4226) | 1176(962 to 1439) | -67.4(-74.6 to -50.3) | 3.1(2.3 to 3.6) | 1.5(1.2 to 1.8) | -50.9(-61.7 to -25.0) |
| Endocrine, metabolic, blood, and immune disorders | 308(193 to 390) | 140(94 to 190) | -54.5(-65.0 to -41.2) | 0.3(0.2 to 0.3) | 0.2(0.1 to 0.2) | -31.4(-47.2 to -11.3) |
| Gynecological diseases | - | - | - | - | - | - |
| Hemoglobinopathies and hemolytic anemias | 614(492 to 745) | 150(124 to 180) | -75.6(-81.6 to -67.2) | 0.5(0.4 to 0.6) | 0.2(0.2 to 0.2) | -63.2(-72.2 to -50.6) |
| Urinary diseases and male infertility | 140(105 to 167) | 31(25 to 41) | -77.8(-83.9 to -64.8) | 0.1(0.1 to 0.1) | 0.0(0.0 to 0.1) | -66.5(-75.7 to -46.9) |
| Respiratory infections and tuberculosis | 5709(4113 to 6706) | 584(478 to 723) | -89.8(-92.2 to -82.4) | 4.8(3.5 to 5.7) | 0.7(0.6 to 0.9) | -84.6(-88.2 to -73.5) |
| Lower respiratory infections | 3624(2230 to 4299) | 449(363 to 556) | -87.6(-90.8 to -73.8) | 3.1(1.9 to 3.6) | 0.6(0.5 to 0.7) | -81.3(-86.1 to -60.5) |
| Otitis media | 10(6 to 17) | 0(0 to 0) | -99.3(-99.7 to -98.1) | 0.0(0.0 to 0.0) | 0.0(0.0 to 0.0) | -99.0(-99.5 to -97.2) |
| Tuberculosis | 1888(1533 to 2206) | 125(101 to 155) | -93.4(-95.0 to 91.2) | 1.6(1.3 to 1.9) | 0.2(0.1 to 0.2) | -90.0(-92.4 to -86.7) |
| Upper respiratory infections | 186(28 to 310) | 10(6 to 23) | -94.6(-97.9 to -44.7) | 0.2(0.0 to 0.3) | 0.0(0.0 to 0.0) | -91.8(-96.8 to -16.6) |
| Skin and subcutaneous diseases | 94(35 to 120) | 13(10 to 20) | -86.2(-90.5 to -62.0) | 0.1(0.0 to 0.1) | 0.0(0.0 to 0.0) | -79.2(-85.7 to -42.8) |
| Bacterial skin diseases | 90(32 to 115) | 9(7 to 14) | -90.5(-93.6 to -70.5) | 0.1(0.0 to 0.1) | 0.0(0.0 to 0.0) | -85.6(-90.3 to -55.5) |
| Decubitus ulcer | 1(1 to 5) | 3(2 to 4) | 140.5(-32.8 to 486.2) | 0.0(0.0 to 0.0) | 0.0(0.0 to 0.0) | 262.5(1.4 to 783.7) |
| Other skin and subcutaneous diseases | 3(1 to 4) | 2(1 to 3) | -47.6(-66.6 to 21.5) | 0.0(0.0 to 0.0) | 0.0(0.0 to 0.0) | -21.0 (-49.6 to 83.2) |
| Substance use disorders | 1168(917 to 1428) | 240(186 to 298) | -79.5(-84.9 to -71.0) | 1.0(0.8 to 1.2) | 0.3(0.2 to 0.4) | -69.0(-77.3 to -56.3) |
| Alcohol use disorders | 168(126 to 226) | 106(60 to 137) | -36.8(-69.7 to -3.9) | 0.1(0.1 to 0.2) | 0.1(0.1 to 0.2) | -4.7(-54.3 to 44.8) |
| Drug use disorders | 999(775 to 1237) | 134(103 to 172) | -86.6(-90.6 to -80.6) | 0.8(0.7 to 1.0) | 0.1(0.1 to 0.2) | -79.9(-85.8 to -70.8) |

**Table S3.** Death numbers and death rates in 1990 and 2019 in China, ages 10–19 years, for female

| Cause | Number of deaths | | | Mortality rate (per 100 000 people) | | |
| --- | --- | --- | --- | --- | --- | --- |
|  | 1990 | 2019 | Percentage change (%) | 1990 | 2019 | Percentage change (%) |
| all cause | 59395(51526 to 69662) | 12550(10893 to 14250) | -78.9(-82.7 to 74.7) | 53.3(46.2 to 62.5) | 18.6(16.1 to 21.2) | -65.0(-71.4 to -58.2) |
| Communicable, maternal, neonatal, and nutritional diseases | 9405 (7915 to 11465) | 902 (781 to 1054) | -90.4(-92.3 to -88.1) | 8.4 (7.1 to 10.2) | 1.3 (1.2 to 1.6) | -84.1(-87.3 to -80.2) |
| Enteric infections | 2351(1413 to 3637) | 287(165 to 462) | -87.1(-91.4 to -80.5) | 1.0(0.6 to 1.6) | 0.2(0.1 to 0.3) | -78.6(-85.7 to -67.7) |
| HIV/AIDS and sexually transmitted infections | 36(26 to 46) | 115(94 to 130) | 224.5(145.1 to 357.2) | 0.0(0.0 to 0.0) | 0.2(0.1 to 0.2) | 436.7(305.4 to 656.2) |
| HIV/AIDS | 16(7 to 25) | 108(87 to 123) | 588.7(340.7 to 1346.4) | 0.0(0.0 to 0.0) | 0.2(0.1 to 0.2) | 1039.3(628.9 to 2292.5) |
| Sexually transmitted infections excluding HIV | 20(14 to 25) | 7(6 to 8) | -66.0(-74.5 to -49.0) | 0.0(0.0 to 0.0) | 0.0(0.0 to 0.0) | -43.7(-57.8 to -15.6) |
| Maternal and neonatal disorders | 1360(1108 to 1671) | 77(62 to 93) | -94.3(-95.8 to -92.5) | 1.2(1.0 to 1.5) | 0.1(0.1 to 0.1) | -90.6(-93.0 to -87.7) |
| Maternal disorders | 1360(1108 to 1671) | 77(62 to 93) | -94.3(-95.8 to -92.5) | 1.2(1.0 to 1.5) | 0.1(0.1 to 0.1) | -90.6(-93.0 to -87.7) |
| Neglected tropical diseases and malaria | 215(71 to 1569) | 14 (7 to 18) | -93.7(-99.2 to -79.5) | 0.2(0.1 to 1.4) | 0.0(0.0 to 0.0) | -89.6(-98.7 to -66.0) |
| Chagas disease | - | - | - | - | - | - |
| Cystic echinococcosis | 2(1 to 4) | 0(0 to 0) | -87.1(-94.7 to -61.5) | 0.0(0.0 to 0.0) | 0.0(0.0 to 0.0) | -78.7(-91.3 to -36.3) |
| Cysticercosis | 3(0 to 7) | 0(0 to 1) | - | 0.0(0.0 to 0.0) | 0.0(0.0 to 0.0) | - |
| Dengue | 1(0 to 1) | 0(0 to 0) | -86.9(-92.1 to -49.1) | 0.0(0.0 to 0.0) | 0.0(0.0 to 0.0) | -78.3(-86.9 to -15.8) |
| Ebola | - | - | - | - | - | - |
| Intestinal nematode infections | 9(5 to 13) | 0(0 to1) | - | 0.0(0.0 to 0.0) | 0.0(0.0 to 0.0) | - |
| Leishmaniasis | - | - | - | - | - | - |
| Malaria | 125(0 to 1482) | 0(0 to 0) | -100.0(-100.0 to -100.0 | 0.1(0.0 to 1.3) | 0.0(0.0 to 0.0) | -100.0(-100.0 to -100.0) |
| Other neglected tropical diseases | 13(4 to 18) | 2(1 to 3) | -82.0(-87.3 to -70.9) | 0.0(0.0 to 0.0) | 0.0(0.0 to 0.0) | -70.3(-79.0 to -51.9) |
| Rabies | 47(27 to 147) | 10(3 to 15) | -78.5(-92.4 to -61.2) | 0.0(0.0 to 0.1) | 0.0(0.0 to 0.0) | -64.4(-87.4 to -35.9) |
| Schistosomiasis | 16(8 to 27) | 0(0 to 1) | -97.9(-99.4 to -94.6) | 0.0(0.0 to 0.0) | 0.0(0.0 to 0.0) | -96.6(-99.1 to -91.1) |
| Yellow fever | - | - | - | - | - | - |
| Zika virus | - | - | - | - | - | - |
| Nutritional deficiencies | 231(188 to 283) | 18(15 to 21) | -92.2 (-94.0 to -89.8) | 0.2(0.2 to 0.3) | 0.0(0.0 to 0.0) | -87.0(-90.1 to -83.1) |
| Other infectious diseases | 2450 1868 to 3465) | 221(186 to 266) | -91.0(-93.4 to -87.8) | 2.2(1.7 to 3.1) | 0.3(0.3 to 0.4) | -85.1(-89.2 to -79.9) |
| Acute hepatitis | 259(196 to 319) | 7(5 to 10) | -97.3(-98.2 to -94.9) | 0.2(0.2 to 0.3) | 0.0(0.0 to 0.0) | -95.5(-97.0 to 91.5) |
| Diphtheria | 5(3 to 8) | 0(0 to 0) | -94.8(-96.9 to -91.1) | 0.0(0.0 to 0.0) | 0.0(0.0 to 0.0) | -91.4(-94.9 to -85.3) |
| Encephalitis | 235(152 to 297) | 65(52 to 97) | -72.3(-80.8 to -46.3) | 0.2(0.1 to 0.3) | 0.1(0.1 to 0.1) | -54.2 (-68.2 to -11.1) |
| Measles | 575(189 to 1349) | 11(4 to 26) | -98.0(-98.7 to -97.0) | 0.5(0.2 to 1.1) | 0.0(0.0 to 0.0) | -96.7(-97.9 to -95.1) |
| Meningitis | 911(771 to 1082) | 103(87 to 119) | -88.7(-91.1 to -85.7) | 0.8(0.7 to 1.0) | 0.2(0.1 to 0.2) | -81.3(-85.4 to -76.3) |
| Other unspecified infectious diseases | 97(43 to 133) | 19(14 to 25) | -80.0(-86.2 to -52.5) | 0.1(0.0 to 0.1) | 0.0(0.0 to 0.0) | -66.8(-77.2 to -21.4) |
| Tetanus | 97(56 to 147) | 7(6 to 8) | -93.1(-95.7 to -87.7) | 0.1(0.1 to 0.1) | 0.0(0.0 to 0.0) | -96.7(-97.9 to -95.1) |
| Varicella and herpes zoster | 60(43 to 79) | 4(3 to 5) | -93.9(-96.0 to -90.7) | 0.1(0.0 to 0.1) | 0.0(0.0 to 0.0) | -90.0(-93.4 to -84.6) |
| Whooping cough | 211(15 to 670) | 5(0 to 14) | -97.8(-99.8 to -73.3) | 0.2(0.0 to 0.6) | 0.0(0.0 to 0.0) | -96.4(-99.7 to -55.8) |
| Respiratory infections and tuberculosis | 4170(3373 to 4918) | 335(282 to 408) | -92.0(-93.8 to -89.0) | 3.7(3.0 to 4.4) | 0.5(0.4 to 0.6) | -86.7(-89.7 to -81.8) |
| Lower respiratory infections | 2239(1481 to 2709) | 243(203 to 301) | -89.1(-91.8 to -83.1) | 2.0(1.3 to 2.4) | 0.4(0.3 to 0.4) | -82.0(-86.5 to -72.0) |
| Otitis media | 6 (0 to 10) | 0(0 to 0) | -96.9(-98.2 to -91.6) | 0.0(0.0 to 0.0) | 0.0(0.0 to 0.0) | -94.8(-97.0 to -86.1) |
| Tuberculosis | 1839(1481 to 2193) | 89(72 to 114) | -95.2(-96.3 to -92.6) | 1.6(1.3 to 2.0) | 0.1(0.1 to 0.2) | -92.0(-93.9 to -87.8) |
| Upper respiratory infections | 86(9 to 151) | 7 (1 to 7) |  | 0.1(0.0 to 0.1) | 0.0(0.0 to 0.0) | - |
| Injuries | 27917 (24048 to 32674) | 5854 (5085 to 6691) | -79.0(-82.8 to -74.4) | 25.0 (21.6 to 29.4) | 8.7 (7.5 to 9.9) | -65.3(-71.6 to -57.6) |
| Self-harm and interpersonal violence | 11618(9840 to 13690) | 1253(1078 to 1457) | -89.2(-91.3 to -86.5) | 10.4(8.8 to 12.3) | 1.9(1.61 to 2.2) | -82.2(-85.7 to -77.6) |
| Conflict and terrorism | 1(1 to 2) | 0(0 to 0) | -99.9(-99.9 to- 99.9) | 0.0(0.0 to 0.0) | 0.0(0.0 to 0.0) | 99.9(-99.9 to -99.8) |
| Executions and police conflict | 13(10 to 17) | 6(6 to 7) | -50.4(-63.1 to -34.7) | 0.0(0.0 to 0.0) | 0.0(0.0 to 0.0) | -18.0(-39.0 to 8.1) |
| Interpersonal violence | 1247(1055 to 1476) | 214(180 to 251) | -82.8(-86.8 to -77.9) | 1.1(0.9 to 1.3) | 0.3(0.3 to 0.4) | -71.6(-78.1 to -63.5) |
| Self-harm | 10357(8729 to 12270) | 1033(884 to 1199) | -90.0(-92.0 to -87.6) | 9.3(7.8 to 11.0) | 1.5(1.3 to 1.8) | -83.5(-86.8 to -79.4) |
| Transport injuries | 6595(5623 to 7806) | 2192(1876 to 2523) | -66.8(-73.8 to -59.0) | 5.9 (5.0 to 7.0) | 3.3(2.8 to 3.7) | -45.0(-56.6 to -32.1) |
| Other unintentional injuries | 260(217 to 312) | 83(70 to 100) | -67.9(-75.6 to -57.6) | 0.2(0.2 to 0.3) | 0.1(0.1 to 0.1) | -47.0(-59.6 to -29.9) |
| Road injuries | 6198(5287 to 7323) | 2140(1832 to 2461) | -65.5(-72.9 to -57.4) | 5.6(4.7 to 6.6) | 3.2(2.7 to 3.7) | -42.9(-55.1 to -29.5) |
| Unintentional injuries | 9703(8303 to 11419) | 2409(2062 to 2764) | -75.2(-80.4 to -69.4) | 8.7(7.5 to 10.2) | 3.6(3.1 to 4.1) | -58.9(-67.5 to -49.4) |
| Adverse effects of medical treatment | 340(196 to 445) | 36(29 to 51) | -89.3(-92.8 to -80.2) | 0.3(0.2 to 0.4) | 0.1(0.0 to 0.1) | -82.4 (-88.1 to -67.3) |
| Animal contact | 150(66 to 194) | 15(11 to 20) | -89.9(-92.9 to -80.5) | 0.1(0.1 to 0.2) | 0.0(0.0 to 0.0) | -83.2(-88.2 to -67.8) |
| Drowning | 6137(5204 to 7246) | 1124(956 to 1297) | -81.7(-85.4 to -77.0) | 5.5(4.7 to 6.5) | 1.7 (1.4 to 1.9) | -69.7(-75.9 to -61.9) |
| Environmental heat and cold exposure | 70(28 to 100) | 11(6 to 15) | -83.9(-89.0 to -75.4) | 0.1(0.0 to 0.1) | 0.0(0.0 to 0.0) | -73.4(-81.8 to -59.4) |
| Exposure to forces of nature | 78(71 to 86) | 8(8 to 9) | -89.4(-89.4 to -89.4) | 0.1(0.1 to 0.1) | 0.0(0.0 to 0.0) | -82.5(-82.5 to -82.5) |
| Exposure to mechanical forces | 372(298 to 569) | 160(94 to 202) | -56.9(-81.1 to -38.3) | 0.3(0.3 to 0.5) | 0.2(0.1 to 0.3) | -28.7 (-68.7 to 2.1) |
| Falls | 826(655 to 1091) | 359(218 to 440) | -56.5(-78.8 to -40.9) | 0.7(0.6 to 1.0) | 0.5(0.3 to 0.7) | -28.1(-64.9 to -2.2) |
| Fire, heat, and hot substances | 371(136 to 523) | 72(45 to 119) | -80.6(-88.6 to -37.2) | 0.3(0.1 to 0.5) | 0.1(0.1 to 0.2) | -67.9(-81.1 to 3.8) |
| Foreign body | 189(163 to 224) | 68(57 to 79) | -64.2(-72.1 to -55.7) | 0.2(0.1 to 0.2) | 0.1(0.1 to 0.1) | -40.8(-53.9 to -26.7) |
| Other transport injuries | 397(312 to 495) | 52(63 to 42) | -87.0(-90.4 to -82.2) | 0.4(0.3 to 0.4) | 0.1(0.1 to 0.1) | -78.4(-84.1 to -70.6) |
| Poisonings | 910(763 to 1149) | 471(390 to 555) | -48.2(-62.6 to -33.7) | 0.8(0.7 to 1.0) | 0.7(0.6 to 0.8) | -14.3(-38.1 to 9.7) |
| Non-communicable diseases | 22073 (19040 to 25749) | 5793 (5019 to 6609) | -73.8(-78.7 to -68.0) | 19.8 (17.1-23.1) | 8.6 (7.4-9.8) | -56.6(-64.8 to -47.1) |
| Cardiovascular diseases | 3777(3167 to 4568) | 828(701 to 959) | -78.1(-83.2 to -72.3) | 3.4(2.8 to 4.1) | 1.2(1.0 to 1.4) | -63.7(-72.1 to -54.1) |
| Aortic aneurysm | 10(8 to 16) | 4(4 to 5) | -58.6(-72.8 to -43.1) | 0.0(0.0 to 0.0) | 0.0(0.0 to 0.0) | -31.4(-55.1 to -5.8) |
| Cardiomyopathy and myocarditis | 310(218 to 589) | 137(87 to 172) | -55.9(-79.7 to -26.6) | 0.3(0.2 to 0.5) | 0.2(0.1 to 0.3) | -27.1(-66.4 to 21.4) |
| Endocarditis | 72(30 to 103) | 13(9 to 21) | -82.2(-89.6 to -55.7) | 0.1(0.0 to 0.1) | 0.0(0.0 to 0.0) | -70.6(-82.9 to -26.8) |
| Hypertensive heart disease | 122(79 to 156) | 19(12 to 25) | -84.6(-89.7 to -74.8) | 0.1(0.1 to 0.1) | 0.0(0.0 to 0.0) | -74.5(-83.0 to -58.4) |
| Ischemic heart disease | 714(577 to 877) | 207(167 to 248) | -70.9(-79.2 to -60.6) | 0.6(0.5 to 0.8) | 0.3(0.2 to 0.4) | -51.9(-65.6 to -34.9) |
| Non-rheumatic valvular heart disease | 29(16 to 41) | 7(5 to 8) | -76.4(-85.3 to -53.3) | 0.0(0.0 to 0.0) | 0.0(0.0 to 0.0) | -61.0(-75.7 to -22.8) |
| Other cardiovascular and circulatory diseases | 282(207 to 360) | 84(64 to 117) | -70.4(-80.1 to -50.7) | 0.3(0.2 to 0.3) | 0.1(0.1 to 0.2) | -51.0(-67.0 to -18.5) |
| Rheumatic heart disease | 870(710 to 1073) | 54(43 to 68) | -93.8(-95.6 to -91.3) | 0.8(0.6 to 1.0) | 0.1(0.1 to 0.1) | -89.7(-92.8 to -85.6) |
| Stroke | 1368(1139 to 1688) | 304(255 to 359) | -77.8(-83.3 to -70.7) | 1.2(1.0 to 1.5) | 0.5(0.4 to 0.5) | -63.3(-72.3 to -51.5) |
| Chronic respiratory diseases | 954(556 to 1197) | 93(76 to 117) | -90.2(-92.8 to -82.4) | 0.9(0.5 to 1.1) | 0.1(0.1 to 0.2) | -83.8(-88.0 to -70.9) |
| Asthma | 255(155 to 410) | 18(13 to 25) | -93.0(-95.9 to -87.6) | 0.2(0.1 to 0.4) | 0.0(0.0 to 0.0) | -88.5(-93.3 to -79.5) |
| Chronic obstructive pulmonary disease | 606(272 to 817) | 43(33 to 61) | -92.8(-95.3 to -83.2) | 0.5(0.2 to 0.7) | 0.1(0.0 to 0.1) | -88.2(-92.2 to -72.3) |
| Interstitial lung disease and pulmonary sarcoidosis | 18(11 to 32) | 7 (4 to 11) | -62.4(-77.9 to -36.3) | 0.0(0.0 to 0.0) | 0.0(0.0 to 0.0) | -37.7(-63.5 to 5.3) |
| Other chronic respiratory diseases | 58(34 to 134) | 23(14 to 30) | -59.8(-84.9 to -20.0) | 0.1(0.0 to 0.1) | 0.0(0.0 to 0.1) | -33.5(-75.0 to 32.3) |
| Pneumoconiosis | 16(10 to 31) | 2(1 to 3) | -87.5(-93.2 to -78.1) | 0.0(0.0 to 0.0) | 0.0(0.0 to 0.0) | -79.4(-88.8 to -63.7) |
| Diabetes and kidney diseases | 1510(1289 to 1768) | 304(257 to 354) | -79.8(-84.0 to -75.1) | 1.4(1.2 to 1.6) | 0.5(0.4 to 0.5) | -66.7(-73.5 to -58.8) |
| Acute glomerulonephritis | 251(192 to 329) | 29(22 to 37) | -88.4(-92.6 to -83.2) | 0.2(0.2 to 0.3) | 0.0(0.0 to 0.1) | -80.8(-87.7 to -72.2) |
| Chronic kidney disease | 997(839 to 1167) | 213(179 to 249) | -78.6(-82.9 to -72.9) | 0.9(0.8 to 1.0) | 0.3(0.3 to 0.4) | -64.6(-71.8 to -55.2) |
| Diabetes mellitus | 262(220 to 311) | 62(52 to 74) | -76.3(-81.5 to -70.2) | 0.2(0.2 to 0.3) | 0.1(0.1 to 0.1) | -60.8(-69.3 to -50.7) |
| Digestive diseases | 1480(1250 to 1763) | 176(147 to 207) | -88.1(-90.7 to -84.8) | 1.3(1.1 to 1.6) | 0.3(0.2 to 0.3) | -80.3(-84.7 to -74.8) |
| Appendicitis | 137(90 to 198) | 11(7 to 15) | -92.1(-95.2 to -86.1) | 0.1(0.1 to 0.2) | 0.0(0.0 to 0.0) | -86.9(-92.1 to -77.0) |
| Cirrhosis and other chronic liver diseases | 573(421 to 694) | 71(57 to 88) | -87.7(-91.0 to -82.2) | 0.5(0.4 to 0.6) | 0.1(0.1 to 0.1) | -79.6(-85.2 to -70.6) |
| Gallbladder and biliary diseases | 58(27 to 76) | 5(4 to 8) | -90.8(-93.8 to -78.3) | 0.1(0.0 to 0.1) | 0.0(0.0 to 0.0) | -84.8(-89.8 to -64.1) |
| Inflammatory bowel disease | 50(23 to 72) | 7(5 to 9) | -86.3(-91.1 to -68.3) | 0.0(0.0 to 0.1) | 0.0(0.0 to 0.0) | -77.3 (-85.3 to -47.6) |
| Inguinal, femoral, and abdominal hernia | 7(2 to 11) | 1(1 to 2) | -83.4(-90.8 to -44.7) | 0.0(0.0 to 0.0) | 0.0(0.0 to 0.0) | -72.6(-84.8 to -8.5) |
| Other digestive diseases | 181(64 to 247) | 16(12 to 21) | -91.2 (-94.7 to -71.6) | 0.2(0.1 to 0.2) | 0.0(0.0 to 0.0) | -85.5(-91.2 to -53.0) |
| Pancreatitis | 95(59 to 133) | 18(12 to 23) | -80.7(-87.6 to -66.9) | 0.1(0.1 to 0.1) | 0.0(0.0 to 0.0) | -68.1(-79.4 to -45.3) |
| Paralytic ileus and intestinal obstruction | 143(98 to 182) | 23(19 to 31) | -83.9(-88.4 to -75.2) | 0.1(0.1 to 0.2) | 0.0(0.0 to 0.1) | -73.4(-80.8 to -59.0) |
| Upper digestive system diseases | 232(181 to 320) | 24(19 to 29) | -89.8(-93.0 to -85.6) | 0.2(0.2 to 0.3) | 0.0(0.0 to 0.0) | -83.2 (-88.4 to -76.2) |
| Vascular intestinal disorders | 5(3 to 9) | 1(1 to 1) | -84.9(-93.3 to -71.0) | 0.0(0.0 to 0.0) | 0.0(0.0 to 0.0) | -75.0(-88.8 to -52.0) |
| Diarrheal diseases | 475(152 to 1045) | 28(10 to 64) | -94.1(-95.9 to -89.3) | 0.4(0.1 to 0.9) | 0.0(0.0 to 0.1) | -90.3(-93.2 to -82.3) |
| Invasive Non-typhoidal Salmonella (iNTS) | 66(23 to 142) | 17(6 to 39) | -74.2(-81.3 to -66.1) | 0.1(0.0 to 0.1) | 0.0(0.0 to 0.1) | -57.3(-69.0 to -43.9) |
| Other intestinal infectious diseases | 12(1 to 30) | 1(0 to 4) | -88.5(-99.8 to 204.6) | 0.0(0.0 to 0.0) | 0.0(0.0 to 0.0) | -81.0(-99.7 to 403.9) |
| Typhoid and paratyphoid | 390(136 to 817) | 76(32 to 147) | -80.6(-87.1 to -68.9) | 0.3(0.1 to 0.7) | 0.1(0.0 to 0.2) | -67.9(-78.7 to -48.5) |
| Maternal and neonatal disorders | 1360(1108 to 1671) | 77(62 to 93) | -94.3(-95.8 to 92.5) | 1.2(1.0 to 1.5) | 0.1(0.0 to 0.1) | -90.6(-93.0 to -87.7) |
| Maternal disorders | 1360(1108 to 1671) | 77(62 to 93) | -94.3(-95.8 to -92.5) | 1.2(1.0 to 1.5) | 0.1(0.1 to 0.1) | -90.6(-93.0 to -87.7) |
| Mental disorders | 3(1 to 5) | 3(2 to 4) | 12.3(-37.7 to 102.3) | 0.0(0.0 to 0.0) | 0.0(0.0 to 0.0) | 85.8(3.1 to 234.7) |
| Eating disorders | 3(1 to 5) | 3(2 to 4) | 12.3(-37.7 to 102.3) | 0.0(0.0 to 0.0) | 0.0(0.0 to 0.0) | 85.8(3.1 to 234.7) |
| Musculoskeletal disorders | 394(307 to 684) | 230(169 to 314) | -41.7(-63.6 to -22.6) | 0.4(0.3 to 0.6) | 0.3(0.3 to 0.5) | -3.6(-39.9 to 28.0) |
| Other musculoskeletal disorders | 364(282 to 636) | 222(165 to 305) | -38.9(-62.4 to -18.8) | 0.3(0.2 to 0.6) | 0.3(0.2 to 0.5) | -1.0(-37.7 to -34.3) |
| Rheumatoid arthritis | 30(16 to 53) | 8(4 to 11) | -75.2(-86.0 to -59.3) | 0.0(0.0 to 0.0) | 0.0(0.0 to 0.0) | -58.9(-76.8 to -32.7) |
| Neoplasms | 8109(6866 to 9468) | 2716(2338 to 3117) | -66.5(-73.0 to -58.4) | 7.3(6.1 to 8.5) | 4.0(3.5 to 4.6) | -44.6(-55.4 to -31.3) |
| Bladder cancer | 21(17 to 27) | 5(4 to 6) | -76.5(-82.5 to -67.8) | 0.0(0.0 to 0.0) | 0.0(0.0 to 0.0) | -61.1(-71.1 to -46.7) |
| Brain and central nervous system cancer | 1081(736 to 1467) | 503(364 to 658) | -53.4(-76.8 to -18.6) | 1.0(0.7 to 1.3) | 0.7(0.5 to 1.0) | -23.0(-61.6 to 34.7) |
| Cervical cancer | 71(50 to 90) | 23(11 to 30) | -68.2(-82.2 to -51.6) | 0.1(0.0 to 0.1) | 0.0(0.0 to 0.0) | -47.3(-70.5 to -19.9) |
| Colon and rectum cancer | 189(158 to 225) | 63(52 to 75) | -66.6(-74.1 to -56.5) | 0.2(0.1 to 0.2) | 0.1(0.1 to 0.1) | -44.7(-57.2 to -28.0) |
| Hodgkin lymphoma | 123 (52 to 178) | 16(12 to 23) | -87.0(-92.0 to -72.5) | 0.1(0.0 to 0.2) | 0.0(0.0 to 0.0) | -78.5(-86.7 to -54.6) |
| Kidney cancer | 46(39 to 55) | 29(24 to 34) | -36.7(-52.1 to -18.8) | 0.0(0.0 to 0.0) | 0.0(0.0 to 0.1) | 4.7(-20.8 to 34.2) |
| Leukemia | 3539(2724 to 4317) | 1042(826 to 1222) | -70.6(-78.7 to -61.3) | 3.2(2.4 to 3.9) | 1.5(1.2 to 1.8) | -51.3(-64.8 to -36.0) |
| Lip and oral cavity cancer | 39(33 to 47) | 12(10 to 14) | -70.4(-77.2 to -61.7) | 0.0(0.0 to 0.0) | 0.0(0.0 to 0.0) | -51.0(-62.3 to -36.7) |
| Liver cancer | 397(321 to 495) | 67(54 to 81) | -83.2(-87.8 to -77.1) | 0.4(0.3 to 0.4) | 0.1(0.1 to 0.1) | -72.3(-79.8 o -62.1) |
| Malignant skin melanoma | 25(15 to 38) | 11(7 to 15) | -55.3(-75.9 to -35.6) | 0.0(0.0 to 0.0) | 0.0(0.0 to 0.0) | -26.0(-60.2 to 6.5) |
| Nasopharynx cancer | 177(142 to 216) | 21(17 to 25) | -88.4(-91.2 to -82.4) | 0.2(0.1 to 0.2) | 0.0(0.0 to 0.0) | -80.8(-85.4 to -70.9) |
| Non-Hodgkin lymphoma | 420(359 to 495) | 161(136 to 188) | -61.7(-69.4 to -51.7) | 0.4(0.3 to 0.4) | 0.2(0.2 to 0.3) | -36.6 (-49.4 to -20.1) |
| Other malignant neoplasms | 1347(1149 to 1580) | 538(454 to 641) | -60.0(-68.8 to -49.2) | 1.2(1.0 to 1.4) | 0.8(0.7 to 1.0) | -33.9(-48.3 to -16.0) |
| Other neoplasms | 28(21 to 52) | 16(13 to 19) | -44.7(-74.2 to -14.9) | 0.0(0.0 to 0.0) | 0.0(0.0 to 0.0) | -8.6(-57.4 to 40.8) |
| Ovarian cancer | 136(81 to 182) | 62(45 to 78) | -54.2(-74.0 to 18.9) | 0.1(0.1 to 0.2) | 0.1(0.1 to 0.1) | -24.2(-57.1 to 34.1) |
| Pancreatic cancer | 20(16 to 23) | 9(7 to 11) | -54.4(-64.9 to -40.6) | 0.0(0.0 to 0.0) | 0.0(0.0 to 0.0) | -24.6(-42.0 to -1.7) |
| Stomach cancer | 172(141 to 205) | 36(30 to 43) | -79.1(-84.0 to -72.9) | 0.2(0.1 to 0.2) | 0.1(0.0 to 0.1) | -65.4(-73.5 to -55.2) |
| Testicular cancer | - | - | - | - | - | - |
| Thyroid cancer | 34(23 to 43) | 8(7 to 10) | -75.7 (-81.9 to -60.0) | 0.0(0.0 to 0.0) | 0.0(0.0 to 0.0) | -59.8(-70.0 to -33.9) |
| Tracheal, bronchus, and lung cancer | 183(152 to 221) | 74(62 to 89) | -59.4(-69.3 to -47.0) | 0.2(0.1 to 0.2) | 0.1(0.1 to 0.1) | -32.9(-49.2 to -12.4) |
| Neurological disorders | 1144(926 to 1424) | 320(262 to 386) | -72.0(-79.0 to -62.3) | 1.0(0.8 to 1.3) | 0.5(0.4 to 0.6) | -53.7 (-65.3 to -37.6) |
| Idiopathic epilepsy | 1008(799 to 1270) | 253(204 to 313) | -74.9(-81.6 to -64.3) | 0.9 (0.7 to 1.1) | 0.4(0.3 to 0.5) | -58.5(-69.6 to -41.0) |
| Motor neuron disease | 22(19 to 26) | 8(7 to 9) | -64.3(-71.8 to -55.1) | 0.0(0.0 to 0.0) | 0.0(0.0 to 0.0) | -41.0(-53.3 to -25.7) |
| Multiple sclerosis | 9(4 to 13) | 3(2 to 4) | -62.8(-81.8 to -39.4) | 0.0(0.0 to 0.0) | 0.0(0.0 to 0.0) | -38.4(-69.8 to 0.2) |
| Other neurological disorders | 104(89 to 123) | 56(48 to 65) | -46.3(-57.2 to -33.5) | 0.1(0.1 to 0.1) | 0.1(0.1 to 0.1) | -11.2(-29.2 to 10.0) |
| Other nutritional deficiencies | 27(21 to 34) | 8(7 to 10) | -70.2(-78.4 to -59.0) | 0.0(0.0 to 0.0) | 0.0(0.0 to 0.0) | -50.7 (-64.2 to -32.2) |
| Protein-energy malnutrition | 204(165 to 251) | 10(8 to 12) | -95.1(-96.2 to -93.5) | 0.2(0.1 to 0.2) | 0.0(0.0 to 0.0) | -91.8(-93.8 to -89.2) |
| Breast cancer | 62(51 to 74) | 22(17 to 27) | -65.0(-74.2 to -52.7) | 0.1(0.0 to 0.1) | 0.0(0.0 to 0.0) | -42.2(-57.3 to -21.7) |
| Other non-communicable diseases | 3482 (2872 to 4240) | 1022(860 to 1213) | -70.6(-77.7 to -61.2) | 3.1(2.6 to 3.8) | 1.5(1.3 to 1.8) | -51.4(-63.1 to -35.8) |
| Congenital birth defects | 2477(1985 to 3074) | 767(637 to 924) | -69.0(-77.4 to -57.3) | 2.2(1.8 to 2.8) | 1.1(0.9 to 1.4) | -48.8(-62.6 to -29.4) |
| Endocrine, metabolic, blood, and immune disorders | 339(181 to 431) | 102(71 to 134) | -70.0(-77.4 to -42.3) | 0.3(0.2 to 0.4) | 0.2(0.1 to 0.2) | -50.4(-62.6 to -4.6) |
| Gynecological diseases | 3(2 to 11) | 3(1 to 4) | 16.5(-85.4 to 105.2) | 0.0(0.0 to 0.0) | 0.0(0.0 to 0.0) | 38.1(-75.8 to 239.5) |
| Hemoglobinopathies and hemolytic anemias | 512(341 to 660) | 125(106 to 149) | -75.6(-82.5 to -60.1) | 0.5(0.3 to 0.6) | 0.2(0.2 to 0.2) | -59.6(-71.1 to -34.0) |
| Urinary diseases and male infertility | 151(102 to 187) | 26(22 to 33) | -82.9(-87.4 to -71.5) | 0.1(0.1 to 0.2) | 0.0(0.0 to 0.0) | -71.7(-79.2 to -52.9) |
| Respiratory infections and tuberculosis | 4170(3373 to 4918) | 335(282 to 408) | -92.0(-93.8 to -89.0) | 3.7(3.0 to 4.4) | 0.5(0.4 to 0.6) | -86.7(-89.7 to -81.8) |
| Lower respiratory infections | 2239(1481 to 2709) | 243(203 to 301) | -89.1(-91.8 to -83.1) | 2.0(1.3 to 2.4) | 0.4(0.3 to 0.4) | -82.0(-86.5 to -72.0) |
| Otitis media | 6(0 to 10) | 0(0 to 0) | -96.9(-98.2 to -91.6) | 0.0(0.0 to 0.0) | 0.0(0.0 to 0.0) | -94.8(-97.0 to -86.1) |
| Tuberculosis | 1839(1481 to 2193) | 89(72 to 114) | -95.2(-96.3 to 92.6) | 1.6(1.3 to 2.0) | 0.1(0.1 to 0.2) | -92.0(-93.9 to -87.8) |
| Upper respiratory infections | 86(9 to 151) | 3(1 to 7) | -96.7(-98.9 to -38.9) | 0.1(0.0 to 0.1) | 0.0(0.0 to 0.0) | -94.6(-98.2 to 1.1) |
| Skin and subcutaneous diseases | 73(39 to 90) | 10(8 to 12) | -86.7(-90.0 to -73.3) | 0.1(0.0 to 0.1) | 0.0(0.0 to 0.0) | -78.1(-55.9 to -83.5) |
| Bacterial skin diseases | 70(37 to 88) | 7(5 to 8) | -90.6(-93.1 to -79.6) | 0.1(0.0 to 0.1) | 0.0(0.0 to 0.0) | -84.5(-88.6 to -66.2) |
| Decubitus ulcer | 1(0 to 3) | 1(0 to 2) | 87.3(-67.5 to 709.7) | 0.0(0.0 to 0.0) | 0.0(0.0 to 0.0) | 209.8(-46.2 to 1239.4) |
| Other skin and subcutaneous diseases | 2(2 to 3) | 2(2 to 3) | -12.1(-46.6 to 44.7) | 0.0(0.0 to 0.0) | 0.0(0.0 to 0.0) | 45.3 (-11.7 to 139.4) |
| Substance use disorders | 1146(933 to 1391) | 89(74 to 107) | -92.2(-94.2 to -89.7) | 1.0(0.8 to 1.2) | 0.1(0.1 to 0.2) | -87.2(-90.4 to -83.0) |
| Alcohol use disorders | 141(115 to 170) | 22(18 to 26) | -84.7(-88.4 to -80.0) | 0.1(0.1 to 0.2) | 0.0(0.0 to 0.0) | -74.7(-67.0 to -80.8) |
| Drug use disorders | 1005(810 to 1229) | 67(55 to 83) | -93.3(-95.1 to -91.0) | 0.9(0.7 to 1.1) | 0.1(0.1 to 0.1) | -88.9(-91.9 to -85.0) |


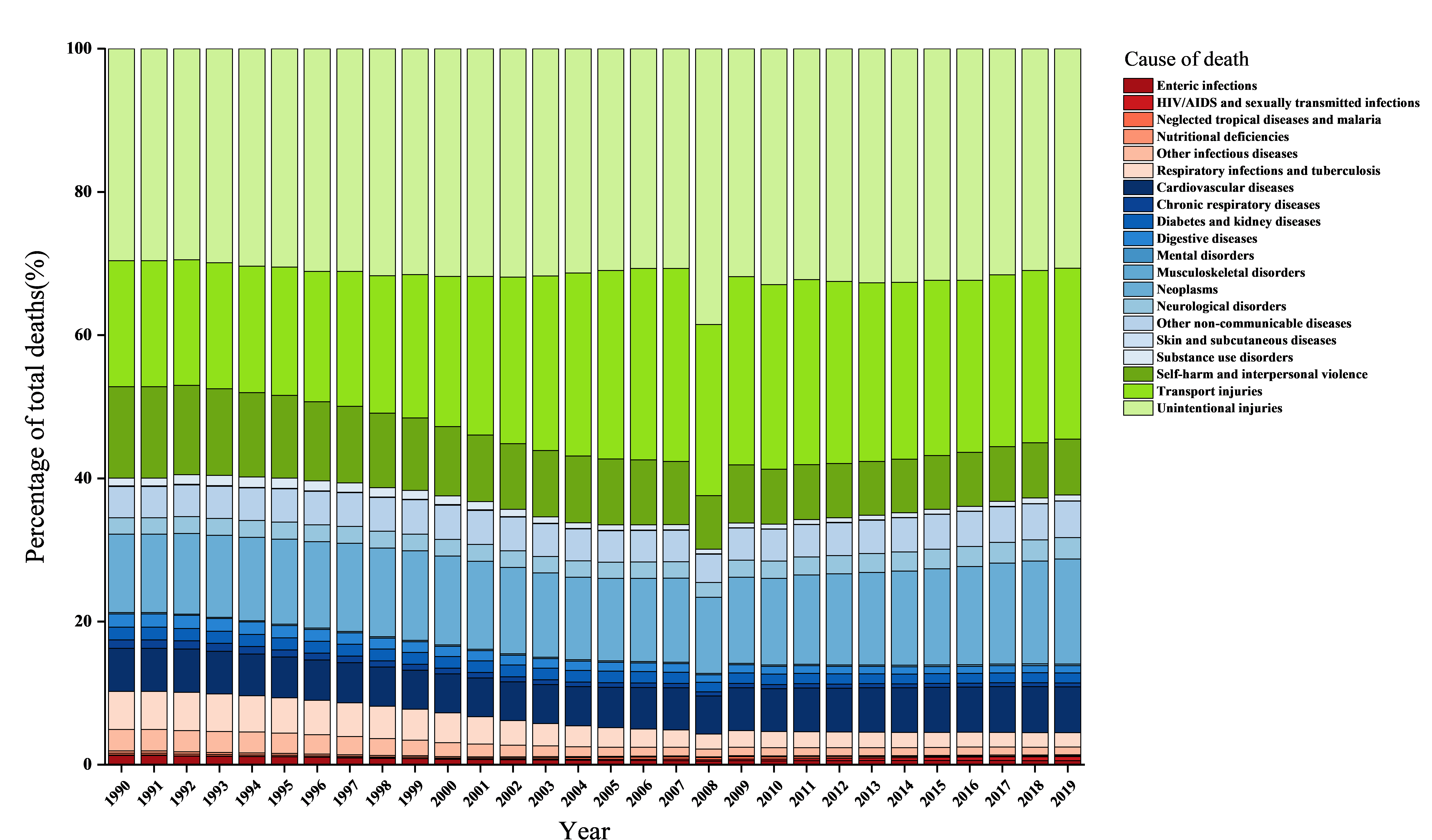


**Figure S1**. Percentage of total deaths by level-2 causes in China, aged 10 –19 years, for males from 1990 to 2019


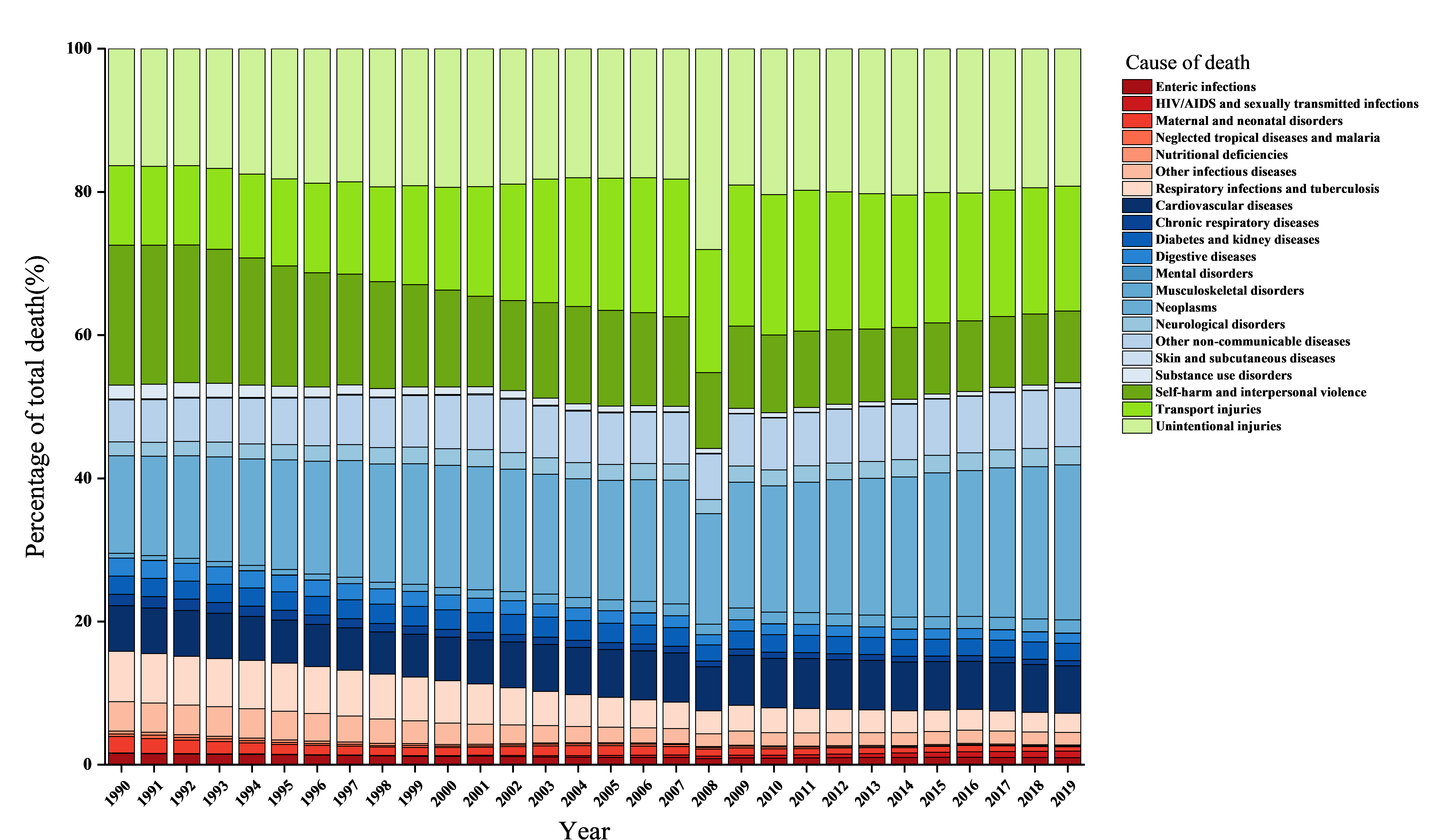


**Figure S2.** Percentage of total deaths by level-2 causes in China, aged 10–19 years, for females from 1990 to 2019


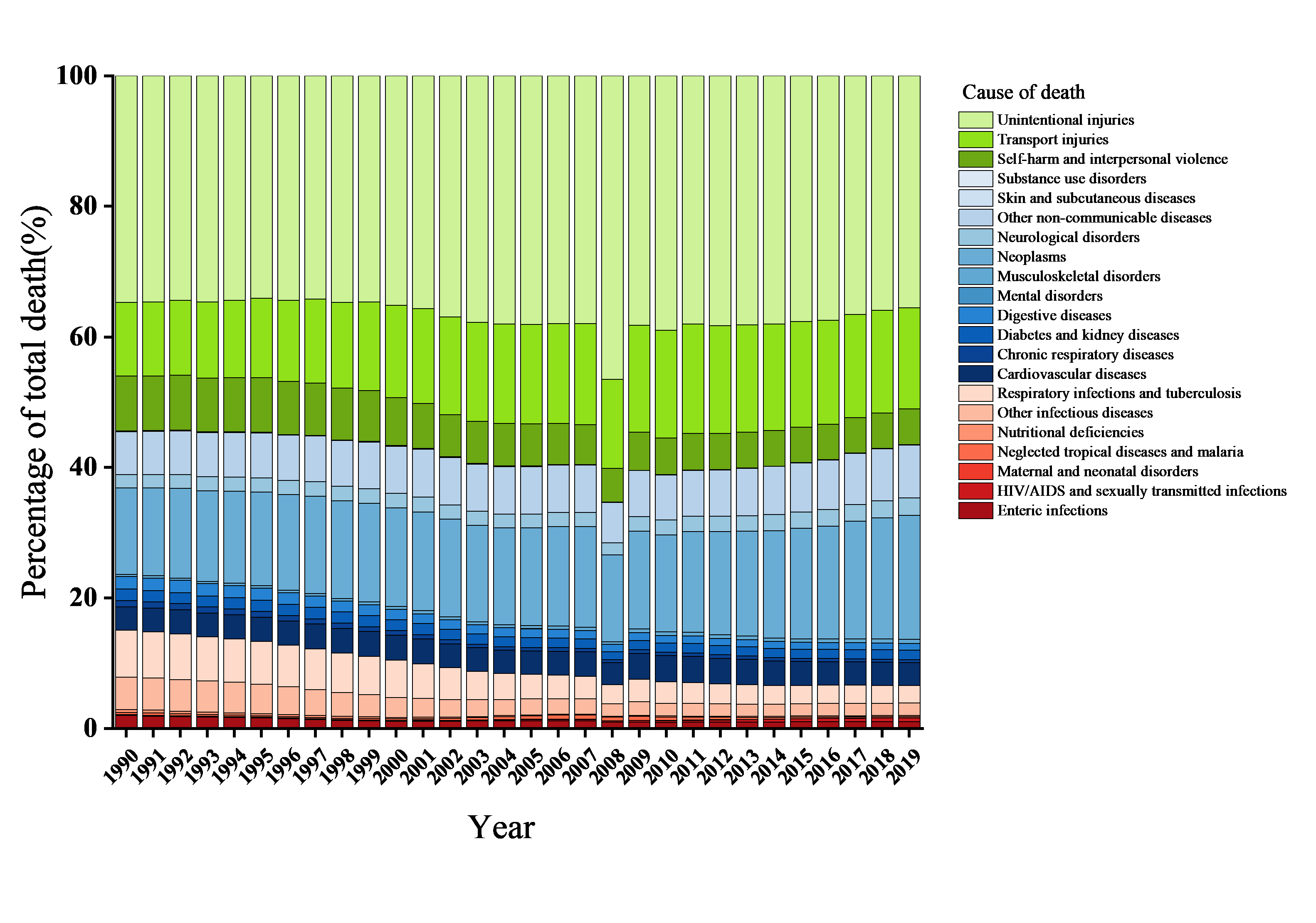


**Figure S3.** Percentage of total deaths by level-2 causes in China, aged 10-14 years from 1990 to 2019


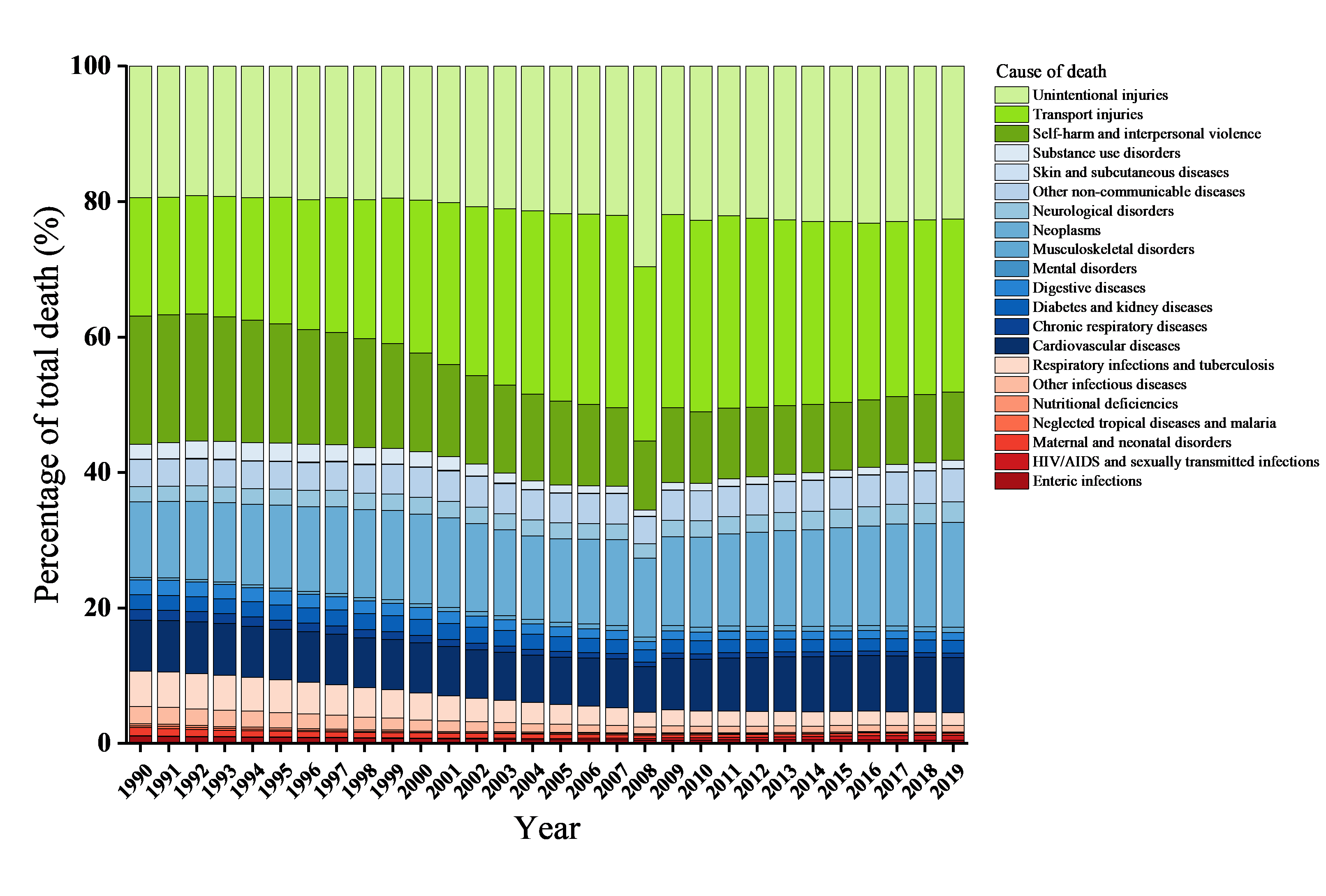


**Figure S4.** Percentage of total deaths by level-2 causes in China, aged 15-19 years from 1990 to 2019


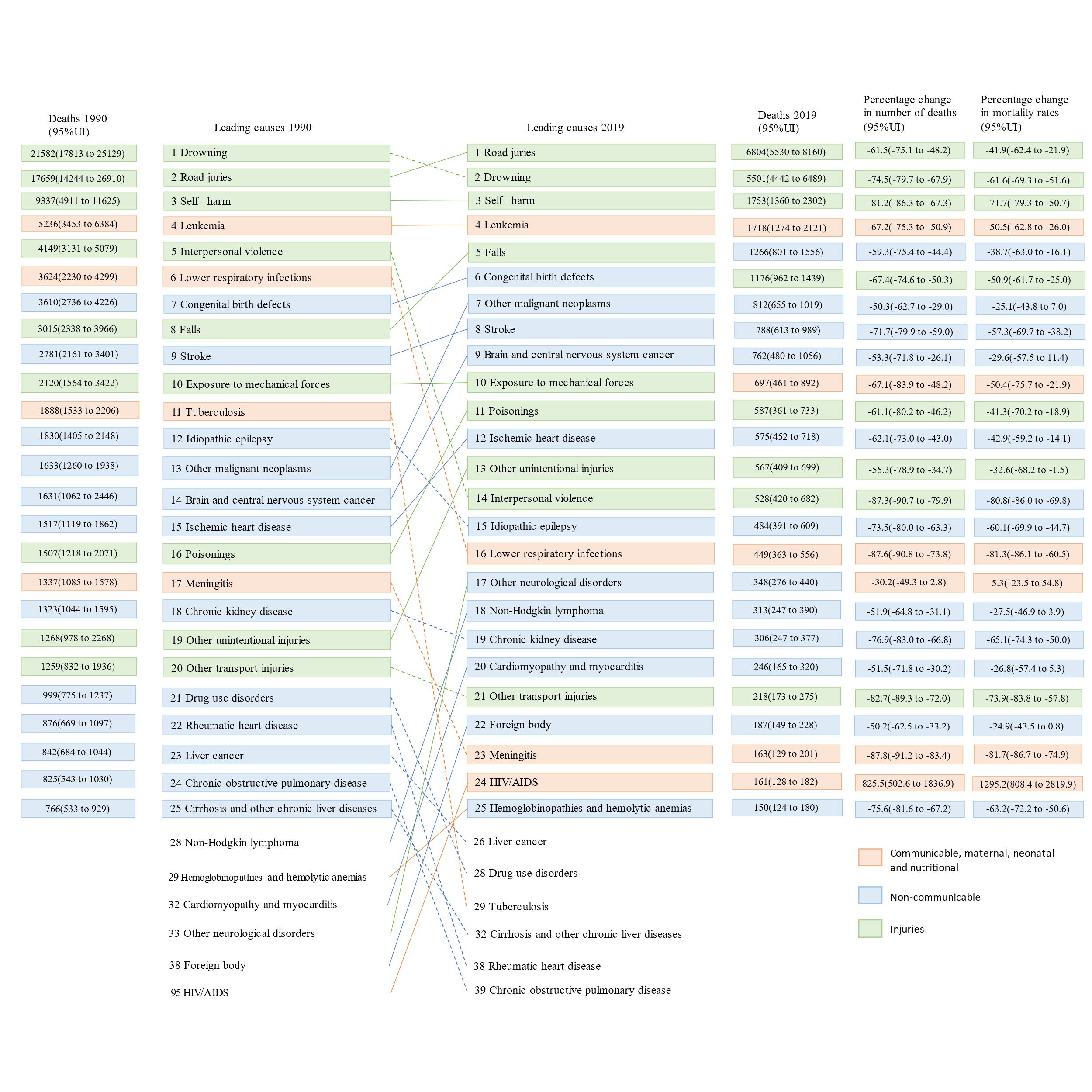


**Figure S5.** Top 25 causes of death in China, aged 10-19 years, for males,1990 and 2019


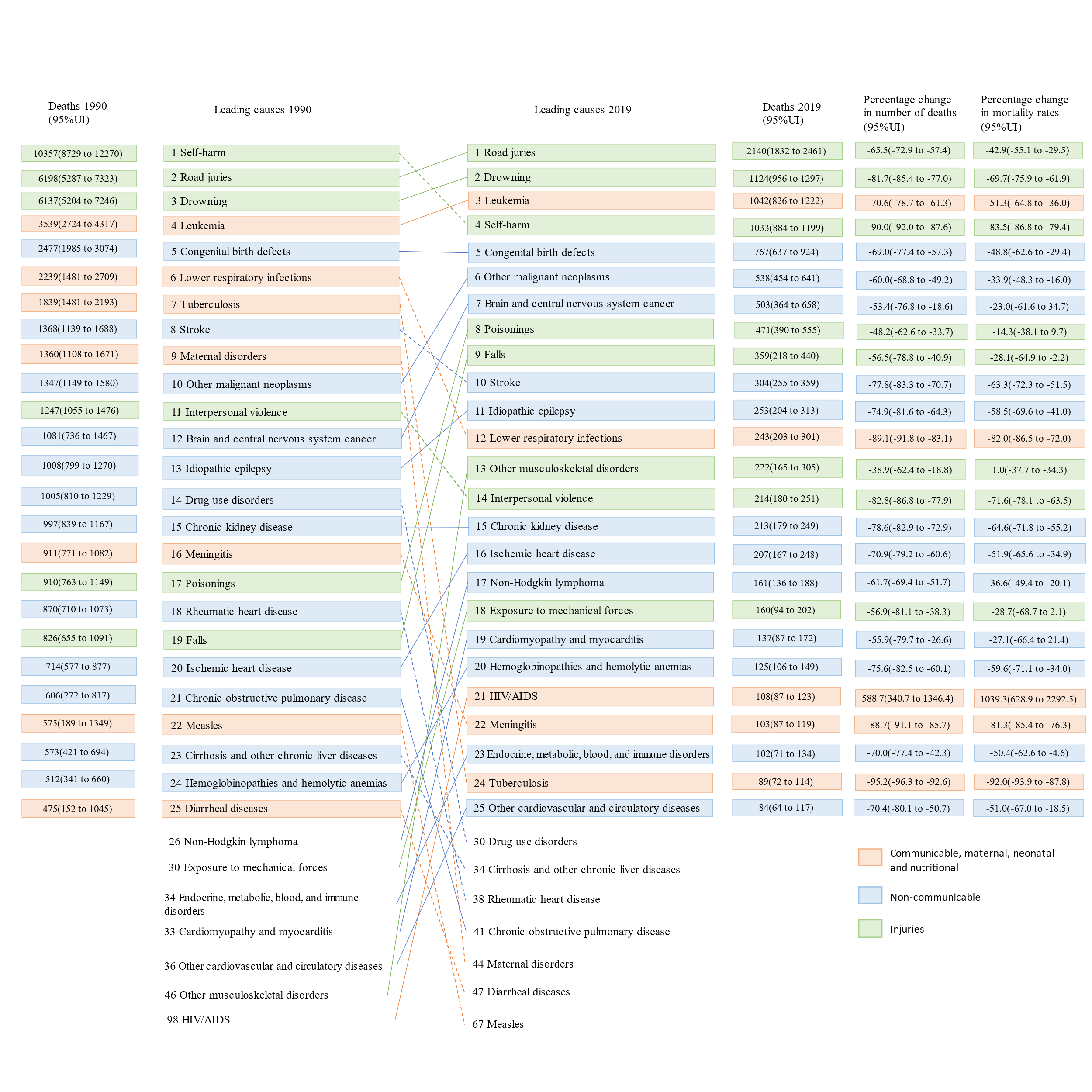


**Figure S6.** Top 25 causes of death in China, aged 10 – 19 years, for females,1990 and 2019

**Table S4.** The proportion of level-1 and level-2 causes of death in China, age 10-19 years, both sexes, 1990 and 2019

| Causes | Percentage of total death(95%UI) | | Difference |
| --- | --- | --- | --- |
|  | 1990 | 2019 |  |
| Communicable, maternal, neonatal, and nutritional diseases | 12.2%(10.5% to 13.8%) | 5.3%(4.8% to 5.9%) | -6.9 |
| Enteric infections | 1.4%(0.9% to 2.1%） | 0.7%(0.4% to 1.1%) | -0.7 |
| HIV/AIDS and sexually transmitted infections | 0.0%(0.0% to 0.1%) | 0.7%(0.5% to 0.8%) | 0.7 |
| Maternal and neonatal disorders | 0.8%(0.7% to 1.0%) | 0.2%(0.1% to 0.2%) | -0.6 |
| Neglected tropical diseases and malaria | 0.3%(0.1% to 1.8%) | 0.1%(0.1% to 0.1%) | -0.2 |
| Nutritional deficiencies | 0.3%(0.3% to 0.4%) | 0.1%(0.1% to 0.2%) | -0.2 |
| Other infectious diseases | 3.4%(2.8% to 4.4%) | 1.3%(1.2% to 1.5%) | -2.9 |
| Respiratory infections and tuberculosis | 5.9%(4.5% to 6.3%) | 2.2%(2.0% to 2.5%) | -3.7 |
| Non-communicable diseases | 32.4%(29.9% to 33.6%) | 37.1%(35.9% to 39.0%) | 4.7 |
| Cardiovascular diseases | 6.1%(5.5% to 6.8%) | 6.5%(6.0% to 7.2%) | 0.4 |
| Chronic respiratory diseases | 1.3%(1.0% to 1.5%) | 0.6%(0.5% to 0.7%) | -0.7 |
| Diabetes and kidney diseases | 2.0%(1.9% to 2.2%) | 1.7%(1.6% to 1.9%) | -0.3 |
| Digestive diseases | 2.1%(1.9% to 2.3%) | 1.1%(1.0% to 1.2%) | -1.0 |
| Mental disorders | 0.0%(0.0% to 0.0%) | 0.0%(0.0% to 0.0%) | 0.0 |
| Musculoskeletal disorders | 0.3%(0.3% to 0.5%) | 0.7%(0.6% to 0.9%) | 0.4 |
| Neoplasms | 11.9%(10.6% to 12.6%) | 16.8%(15.7% to 17.9%) | 4.9 |
| Neurological disorders | 2.2%(1.9% to 2.3%) | 2.9%(2.7% to 3.3%) | 0.7 |
| Other non-communicable diseases | 4.9%(4.4% to 5.3%) | 6.0%(5.6% to 6.7%) | 1.1 |
| Skin and subcutaneous diseases | 0.1%(0.1% to 0.1%) | 0.1%(0.1% to 0.2%) | 0.0 |
| Substance use disorders | 1.4%(1.2% to 1.5%) | 0.8%(0.7% to 0.9%) | -0.6 |
| Injuries | 55.4%(53.7% to 29.1%) | 57.6%(55.4% to 59.0%) | 2.2 |
| Transport injuries | 15.3%(23.6% to 26.9%) | 22.0%(20.0% to 23.5%) | 6.7 |
| Unintentional injuries | 24.9%(23.6% to 26.9%) | 27.2%(24.0% to 28.5%) | 2.3 |
| Self-harm and interpersonal violence | 15.2%(12.1% to 16.3%) | 8.5%(9.8% to 7.9%) | -6.7 |

Difference = P_2019_ － P_1990_, where P_2019_ refers to proportion of deaths for a particular cause relative to deaths from all causes in 2019 and P_1990_ refers to proportion of deaths for a particular cause relative to deaths from all causes in 1990

**Table S5.** The proportion of level-1 and level-2 causes of death in China, age 10-19 years, for males, 1990 and 2019

| Causes | Proportion of all causes of death(95%UI) | | Difference |
| --- | --- | --- | --- |
|  | 1990 | 2019 |  |
| Communicable, maternal, neonatal, and nutritional diseases | 10.2%(8.2% to 11.6%) | 4.5%(4.1% to 5.2%) | -5.7 |
| Enteric infections | 1.4%(0.9% to 2.1%） | 0.7%(0.4% to 1.1%) | -0.7 |
| HIV/AIDS and sexually transmitted infections | 0.0%(0.0% to 0.1%) | 0.7%(0.5% to 0.8%) | 0.7 |
| Neglected tropical diseases and malaria | 0.3%(0.1% to 1.8%) | 0.1%(0.1% to 0.1%) | -0.2 |
| Nutritional deficiencies | 0.3%(0.3% to 0.4%) | 0.1%(0.1% to 0.2%) | -0.2 |
| Other infectious diseases | 3.4%(2.8% to 4.4%) | 1.3%(1.2% to 1.5%) | -2.1 |
| Respiratory infections and tuberculosis | 5.9%(4.5% to 6.3%) | 2.2%(2.0% to 2.5%) | -3.7 |
| Non-communicable diseases | 29.8%(26.2% to 31.3%) | 33.2%(31.8% to 35.8%) | 3.4 |
| Cardiovascular diseases | 6.0%(5.0% to 6.7%) | 6.4%(5.9% to 7.3%) | 0.4 |
| Chronic respiratory diseases | 1.2%(0.8% to 1.4%) | 0.5%(0.5% to 0.6%) | -0.7 |
| Diabetes and kidney diseases | 1.8%(1.5% to 2.0%) | 1.4%(1.3% to 1.6%) | -0.4 |
| Digestive diseases | 1.9%(1.6% to 2.1%) | 1.0%(0.9% to 1.1%) | -0.9 |
| Mental disorders | 0.0%(0.0% to 0.0%) | 0.0%(0.0% to 0.0%) | 0.0 |
| Musculoskeletal disorders | 0.2%(0.1% to 0.2%) | 0.2%(0.2% to 0.3%) | 0.4 |
| Neoplasms | 10.8%(9.0% to 11.9%) | 14.7%(13.3% to 16.1%) | 3.9 |
| Neurological disorders | 2.3%(1.9% to 2.5%) | 3.0%(2.8% to 3.5%) | 0.7 |
| Other non-communicable diseases | 4.4%(3.6% to 4.7%) | 5.1%(4.7% to 6.0%) | 0.7 |
| Skin and subcutaneous diseases | 0.1%(0.0% to 0.1%) | 0.0%(0.0% to 0.1%) | -0.1 |
| Substance use disorders | 1.1%(0.1% to 1.2%) | 0.8%(0.7% to 0.9%) | -0.3 |
| Injuries | 60.0%(58.0% to 65.3%) | 62.3%(59.1% to 63.7%) | 2.3 |
| Transport injuries | 17.6%(15.7% to 26.3%) | 23.9%(21.3% to 25.9%) | 6.3 |
| Unintentional injuries | 29.6%(27.8% to 32.3%) | 30.6%(26.6% to 32.2%) | 1.0 |
| Self-harm and interpersonal violence | 12.8%(8.0% to 14.2%) | 7.8%(7.0% to 9.5%) | -5.0 |

**Table S6.** The proportion of level-1 and level-2 causes of death in China, age 10-19 years, for females, 1990 and 2019

| Causes | Proportion of all causes of death(95%UI) | | Difference |
| --- | --- | --- | --- |
|  | 1990 | 2019 |  |
| Communicable, maternal, neonatal, and nutritional diseases | 15.8%(14.3% to 18.1%) | 7.2%(6.6% to 8.0%) | -8.6 |
| Enteric infections | 1.6%(0.9% to 2.7%） | 1.0%(0.6% to 1.6%) | -0.6 |
| HIV/AIDS and sexually transmitted infections | 0.1%(0.0% to 0.1%) | 1.0%(0.7% to 0.8%) | 0.9 |
| Maternal and neonatal disorders | 0.8%(0.7% to 1.0%) | 0.2%(0.1% to 1.1%) | -0.6 |
| Neglected tropical diseases and malaria | 0.4%(0.1% to 2.3%) | 0.1%(0.1% to 0.1%) | -0.3 |
| Nutritional deficiencies | 0.3%(0.3% to 0.4%) | 0.1%(0.1% to 0.2%) | -0.2 |
| Other infectious diseases | 4.1%(3.3% to 5.5%) | 1.8%(1.6% to 2.0%) | -2.3 |
| Respiratory infections and tuberculosis | 7.0%(5.8% to 7.6%) | 2.7%(2.4% to 3.2%) | -4.3 |
| Non-communicable diseases | 37.2%(35.6% to 38.5%) | 46.2%(44.8% to 47.6%) | 9.0 |
| Cardiovascular diseases | 6.4%(5.8% to 7.0%) | 6.6%(6.1% to 7.1%) | 0.2 |
| Chronic respiratory diseases | 1.6%(0.9% to 1.9%) | 0.7%(0.6% to 0.9%) | -0.9 |
| Diabetes and kidney diseases | 2.5%(2.4% to 2.8%) | 2.4%(2.3% to 2.6%) | -0.1 |
| Digestive diseases | 2.5%(2.3% to 2.6%) | 1.4%(1.3% to 1.6%) | -1.1 |
| Mental disorders | 0.0%(0.0% to 0.0%) | 0.0%(0.0% to 0.0%) | 0.0 |
| Musculoskeletal disorders | 0.7%(0.6% to 1.1%) | 1.8%(1.4% to 2.5%) | 0.9 |
| Neoplasms | 13.7%(12.4% to 14.5%) | 21.6%(20.5% to 22.8%) | 7.9 |
| Neurological disorders | 1.9%(1.7% to 2.4%) | 2.6%(2.3% to 3.0%) | 0.7 |
| Other non-communicable diseases | 5.9%(5.2% to 6.6%) | 8.1%(7.5% to 8.9%) | 2.2 |
| Skin and subcutaneous diseases | 0.1%(0.1% to 0.1%) | 0.1%(0.1% to 0.1%) | 0.0 |
| Substance use disorders | 1.9%(1.7% to 2.2%) | 0.7%(0.6% to 0.8%) | -0.8 |
| Injuries | 47.0%(45.2% to 48.7%) | 46.7%(45.1% to 47.8%) | -0.3 |
| Transport injuries | 11.1%(10.4% to 11.9%) | 17.5%(16.4% to 18.6%) | 6.4 |
| Unintentional injuries | 16.3%(15.4% to 17.6%) | 19.2%(17.5% to 20.1%) | 2.9 |
| Self-harm and interpersonal violence | 19.6%(18.4% to 20.8%) | 10.0%(9.4% to 10.7%) | -9.6 |

**Table S7.** The proportion of level-1 and level-2 causes of death in China, age 10-14 years, 1990 and 2019

| Causes | Proportion of all causes of death(95%UI) | | Difference |
| --- | --- | --- | --- |
|  | 1990 | 2019 |  |
| Communicable, maternal, neonatal, and nutritional diseases | 15.6%(12.8% to 17.1%) | 6.6%(6.0% to 7.5%) | -9.0 |
| Enteric infections | 2.0%(1.3% to 3.0%） | 1.0%(0.6% to 1.7%) | -1.0 |
| HIV/AIDS and sexually transmitted infections | 0.0%(0.0% to 0.0%) | 0.6%(0.5% to 0.7%) | 0.6 |
| Maternal and neonatal disorders | 0.1%(0.1% to 0.1%) | 0.0%(0.0% to 0.0%) | -0.1 |
| Neglected tropical diseases and malaria | 0.4%(0.2% to 1.5%) | 0.2%(0.1% to 0.3%) | -0.2 |
| Nutritional deficiencies | 0.4%(0.4% to 0.5%) | 0.2%(0.1% to 0.2%) | -0.2 |
| Other infectious diseases | 5.0%(3.8% to 6.8%) | 1.9%(1.7% to 2.2%) | -3.1 |
| Respiratory infections and tuberculosis | 7.2%(5.4% to 7.8%) | 2.7%(2.5% to 3.1%) | -5.0 |
| Non-communicable diseases | 30.5%(28.5% to 31.9%) | 36.9%(35.2% to 39.3%) | 6.4 |
| Cardiovascular diseases | 3.6%(3.2% to 4.0%) | 3.5%(3.2% to 3.9%) | -0.1 |
| Chronic respiratory diseases | 1.0%(0.7% to 1.1%) | 0.5%(0.4% to 0.5%) | -0.5 |
| Diabetes and kidney diseases | 1.8%(1.6% to 1.9%) | 1.4%(1.3% to 1.5%) | -0.4 |
| Digestive diseases | 1.9%(1.7% to 2.1%) | 1.0%(0.9% to 1.2%) | -0.9 |
| Mental disorders | 0.0%(0.0% to 0.0%) | 0.0%(0.0% to 0.0%) | 0.0 |
| Musculoskeletal disorders | 0.3%(0.3% to 0.4%) | 0.6%(0.5% to 0.8%) | 0.3 |
| Neoplasms | 13.3%(11.4% to 14.3%) | 19.0%(17.7% to 21.0%) | 5.7 |
| Neurological disorders | 2.0%(1.8% to 2.2%) | 2.6%(2.4% to 3.1%) | 0.6 |
| Other non-communicable diseases | 6.6%(5.9% to 7.2%) | 8.1%(7.5% to 9.3%) | 1.5 |
| Skin and subcutaneous diseases | 0.1%(0.1% to 0.1%) | 0.1%(0.1% to 0.1%) | 0.0 |
| Substance use disorders | 0.0%(0.0% to 0.0%) | 0.0%(0.0% to 0.0%) | 0.0 |
| Injuries | 54.4%(52.4% to 58.0%) | 56.5%(53.8% to 58.3%) | 2.1 |
| Transport injuries | 11.4%(10.3% to 14.7%) | 15.6%(13.8% to 16.7%) | 4.2 |
| Unintentional injuries | 34.7%(32.9% to 37.1%) | 35.5%(33.1% to 37.2%) | 0.8 |
| Self-harm and interpersonal violence | 8.4%(6.8% to 9.1%) | 5.5%(4.9% to 6.2%) | -2.9 |

**Table S8.** The proportion of level-1 and level-2 causes of death in China, age 15-19 years, 1990 and 2019

| Causes | Proportion of all causes of death(95%UI) | | Difference |
| --- | --- | --- | --- |
|  | 1990 | 2019 |  |
| Communicable, maternal, neonatal, and nutritional diseases | 10.7%(9.1% to 12.4%) | 4.5%(4.2% to 5.1%) | -6.2 |
| Enteric infections | 1.1%(0.6% to 1.7%） | 0.5%(0.3% to 0.8%) | -0.6 |
| HIV/AIDS and sexually transmitted infections | 0.1%(0.0% to 0.1%) | 0.7%(0.5% to 0.9%) | 0.6 |
| Maternal and neonatal disorders | 1.2%(1.0% to 1.5%) | 0.3%(0.2% to 0.3%) | -0.9 |
| Neglected tropical diseases and malaria | 0.3%(0.1% to 0.4%) | 0.1%(0.0% to 0.1%) | -0.2 |
| Nutritional deficiencies | 0.3%(0.3% to 0.4%) | 0.1%(0.1% to 0.2%) | -0.2 |
| Other infectious diseases | 2.5%(2.1% to 3.0%) | 0.9%(0.9% to 1.1%) | -1.6 |
| Respiratory infections and tuberculosis | 4.9%(4.5% to 5.4%) | 1.9%(1.8% to 2.2%) | -3.0 |
| Non-communicable diseases | 33.5%(30.5% to 34.8%) | 37.2%(36.0% to 39.4%) | 3.7 |
| Cardiovascular diseases | 7.6%(6.7% to 8.3%) | 8.1%(7.5% to 9.1%) | 0.5 |
| Chronic respiratory diseases | 1.5%(1.1% to 1.7%) | 0.7%(0.6% to 0.8%) | -0.8 |
| Diabetes and kidney diseases | 2.2%(2.0% to 2.4%) | 1.9%(1.7% to 2.1%) | -0.4 |
| Digestive diseases | 2.2%(2.0% to 2.4%) | 1.2%(1.1% to 1.3%) | -1.0 |
| Mental disorders | 0.0%(0.0% to 0.0%) | 0.0%(0.0% to 0.0%) | 0.0 |
| Musculoskeletal disorders | 0.4%(0.3% to 0.6%) | 0.8%(0.6% to 1.0%) | 0.4 |
| Neoplasms | 11.2%(9.9% to 11.9%) | 15.5%(14.4% to 16.4%) | 4.3 |
| Neurological disorders | 2.2%(1.9% to 2.4%) | 3.0%(2.8% to 3.5%) | 0.8 |
| Other non-communicable diseases | 4.0%(3.6% to 4.3%) | 4.9%(4.5% to 5.4%) | 0.9 |
| Skin and subcutaneous diseases | 0.1%(0.1% to 0.1%) | 0.1%(0.0% to 0.1%) | 0.0 |
| Substance use disorders | 2.2%(1.9% to 2.4%) | 1.2%(1.1% to 1.3%) | 1.0 |
| Injuries | 55.9%(54.1% to 59.9%) | 58.2%(55.7% to 59.6%) | 2.3 |
| Transport injuries | 17.5%(15.7% to 24.5%) | 25.5%(23.2% to 27.6%) | 8.0 |
| Unintentional injuries | 19.4%(18.0% to 21.4%) | 22.6%(19.0% to 24.1%) | 3.2 |
| Self-harm and interpersonal violence | 19.0%(14.9% to 20.5%) | 10.1%(9.4% to 11.9%) | -8.9 |

**Table S9.** Predictions of death rate for non-communicable disease in China, aged 10 – 19 years, both sexes

| Causes |  | Mortality rates(95%UI) | | 2015-2030  Percentage change (%) | 2016-2030  Percentage change (%) |
| --- | --- | --- | --- | --- | --- |
|  | 2015 | 2016 | 2030 |  |  |
| Non-communicable diseases | 11.31(10.30 to 12.44) | 11.07(10.02 to 12.34) | 6.04(3.23 to 8.85) | -46.60* | -45.4* |
| Neoplasms | 4.98(4.50 to 5.49) | 4.91(4.44 to 5.50) | 3.46(1.95 to 4.97) | -30.96* | -29.5 |
| Cardiovascular diseases | 2.08(1.85 to 2.36) | 2.00(1.77 to 2.28) | 1.13(0.37 to 1.90) | -45.67* | -43.5* |
| Chronic respiratory diseases | 0.20(0.18 to 0.23) | 0.19(0.17 to 0.23) | 0.09(-0.21 to 0.40) | -55.00* | -52.6* |
| Diabetes and kidney diseases | 0.54(0.49 to 0.60) | 0.52(0.47 to 0.58) | 0.14(-0.07 to 0.35) | -74.07* | -73.1* |

*Note: a.*Rates of percentage change are reduced by more than a third.*

*b. Percentage change = (P_2030_ － P_2015)_/P_2015_*100%*

*Percentage change = (P_2030_ － P_2016)_/P_2016_*100%*

**Table S10.** Predictions of death rate for non-communicable disease in China, aged 10 – 19 years, for males

| Causes |  | Deaths(95%UI) | | 2015-2030  Percentage change (%) | 2016-2030  Percentage change (%) |
| --- | --- | --- | --- | --- | --- |
|  | 2015 | 2016 | 2030 |  |  |
| Non-communicable diseases | 13.15(11.54 to 15.03) | 12.89(11.12 to 14.89) | 9.73(5.33 to 14.12) | -26.01 | -24.5 |
| Neoplasms | 5.66(4.89 to 6.53) | 5.60(4.85 to 6.53) | 4.07(3.04 to 5.11) | -28.09 | -27.3 |
| Cardiovascular diseases | 2.65(2.29 to 3.12) | 2.56(2.14 to 3.04) | 1.60(-1.49 to 4.68) | -39.62* | -37.5* |
| Chronic respiratory diseases | 0.23(0.20 to 0.27) | 0.22(0.19 to 0.27) | 0.10(-0.31 to 0.52) | -56.52* | -54.5* |
| Diabetes and kidney diseases | 0.58(0.50 to 0.67) | 0.56(0.48 to 0.66) | 0.13(-0.02 to 0.29) | -77.59* | -76.8* |

**Table S11.** Predictions of death rate for non-communicable disease in China, aged 10 – 19 years, for females

| Causes |  | Deaths(95%UI) | | 2015-2030  Percentage change (%) | 2016-2030  Percentage change (%) |
| --- | --- | --- | --- | --- | --- |
|  | 2015 | 2016 | 2030 |  |  |
| Non-communicable diseases | 9.19(8.33 to 10.08) | 8.96(8.09 to 9.90) | 4.45(0.65 to 8.26) | -51.58* | -50.3* |
| Neoplasms | 4.18(3.76 to 4.61) | 4.11(3.65 to 4.54) | 3.95(0.85 to 7.05) | -5.50 | -3.9 |
| Cardiovascular diseases | 1.41(1.26 to 1.57) | 1.36(1.20 to 1.53) | 0.42(-0.03 to 0.87) | -70.21* | -69.1* |
| Chronic respiratory diseases | 0.16(0.14 to 0.21 | 0.16(0.13 to 0.20) | 0.07(-0.19 to 0.32) | -56.25* | -56.3* |
| Diabetes and kidney diseases | 0.49(0.44 to 0.55) | 0.48(0.42 to 0.53) | 0.13(-0.12 to 0.37) | -73.47* | -72.9* |


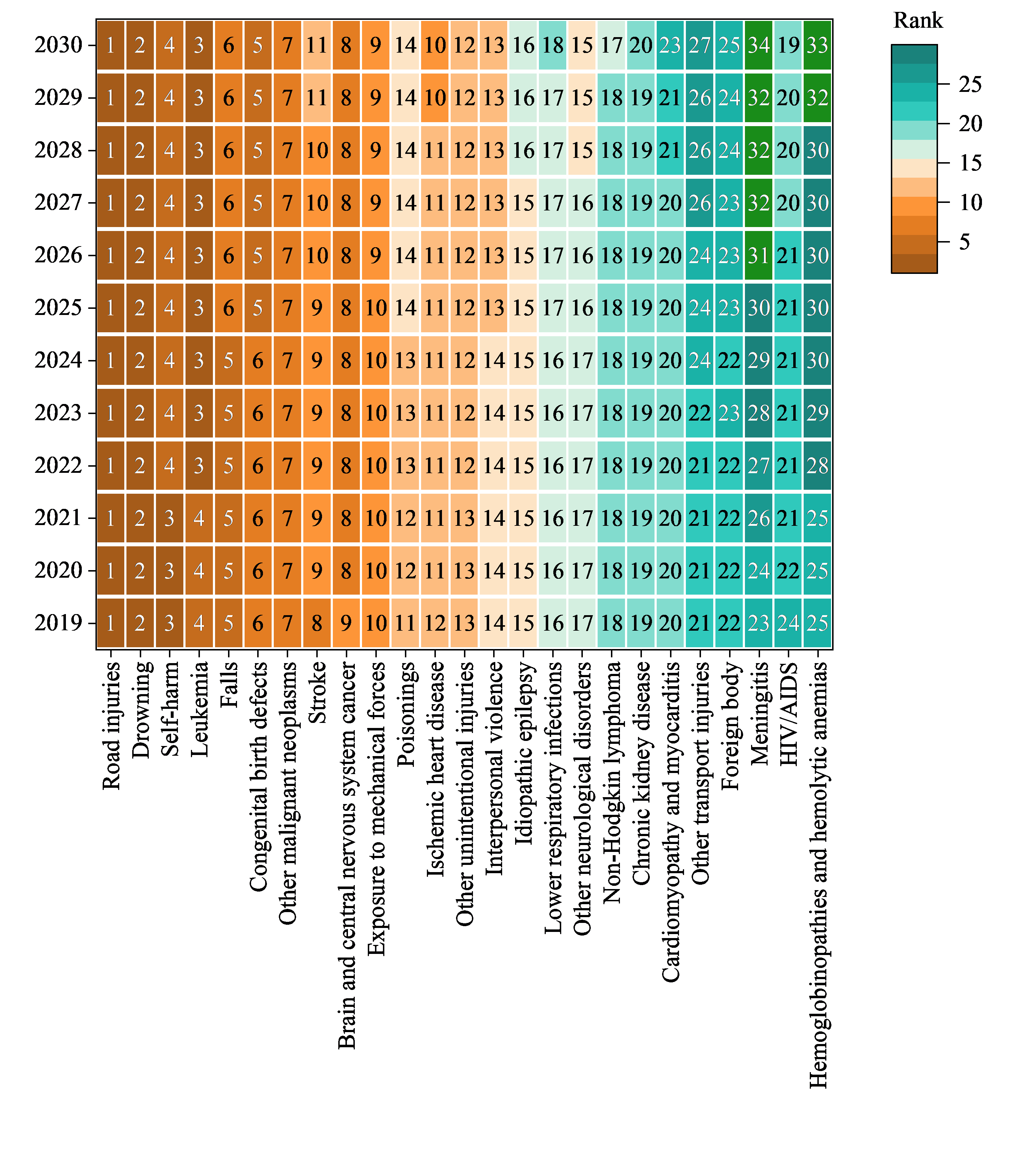


**Figure S7.** Rank of the top 25 causes of death in China, aged 10 – 19 years, for males from 2019 to 2030

**Figure S8.** Rank of the top 25 causes of death in China, aged 10 – 19 years, for females from 2019 to 2030
