# Supplementary material for: Trends in mortality and causes of death among Chinese adolescents aged 10–19 years from 1990 to 2019
Source: Front Public Health. 2023 Feb 7;11:1075858. doi: 10.3389/fpubh.2023.1075858 (PMC9941149; doi:10.3389/fpubh.2023.1075858)
Supplement: Supplementary file 1 [file Data_Sheet_1.ZIP › supplement-xiu/Supplement 2.docx]

# Autoregressive Integrated Moving Average model (ARIMA)

The ARIMA model is a mature Box-Jenkins method used to carry out time series analysis. Optimal parameters of ARIMA could be determined by Akaike's information Criterion(AIC) or Schwarz's Bayesian Criterion(BIC). Ljung-Box test is used to examine autocorrelation and Mean Absolute Percent Error (MAPE) is a common metric to test the prediction validity of the model.

Modeling of ARIMA includes three contents: autoregression, moving average, difference and accumulation. The model is generally denoted as ARIMA (p, d, q), in which p, d, and q refers to the autoregression, difference, and moving average, respectively. ARIMA model is given by

*φ*(*B*)▽*^d^x_t_* = *δ* + *θ*(*B*)*w_t_*,

where *x_t_* is the sequence value at time *t* and *w_t_* is the usual Gaussian white noise process. ▽, *B* and *δ* are on behalf of difference operator, backward shift operator, and constant term respectively. The autoregressive and moving average components are represented by polynomials *φ*(*B*) of order *p* and *θ*(*B*) of order *q* respectively, and the difference component is represented by ▽*^d^* of order *d*.

The specific expressions of each polynomial are

*φ(B)* *=* 1 − *φ*_1_*B* − *φ*_2_*B*^2^ − ··· − *φ_p_B^p^*,

*θ(B)* *= 1* + *θ*_1_*B* + *θ*_2_*B*^2^ + ··· + *θ_q_B^q^*,

▽*^d^* = (1 − *B*)*^d^*.

In general, smoothing, building, diagnosing and forecasting are four key steps in ARIMA modeling. Therefore, in the first step, the stationary characteristic of a sequence should be examined due to ARIMA model requiring stationary sequences. In the second step, optimal parameters of ARIMA are selected by observing autocorrelation function (ACF) and partial autocorrelation function (PACF), and the best ARIMA model is constructed accordingly. Next, Ljung-Box test for modeling residuals is applied to diagnose if there is autocorrelation in the model sequence. To verify the model’s predictive validity, forecasts are made for the past years and MAPE is calculated. Finally, the model is applied to make predictions for unknow data in the future.

Cite: Shumway RH, Stoffer DS. Time Series Analysis and Its Applications: With R Examples, Third Edition: Springer (2010).
