# Supplementary material for: Trends in mortality and causes of death among Chinese adolescents aged 10–19 years from 1990 to 2019
Source: Front Public Health. 2023 Feb 7;11:1075858. doi: 10.3389/fpubh.2023.1075858 (PMC9941149; doi:10.3389/fpubh.2023.1075858)
Supplement: Supplementary file 1 [file Data_Sheet_1.ZIP › supplement-xiu/Supplement 3.docx]

**Table S1.** The orders and coefficients of ARIMA models for the mortality rates of NCDs both sexes

| VAL | ARIMA (p, d, q) | AR (1) | | AR (2) | | MA (1) | | MA (2) | | Drift | | MAPE | LB |
| --- | --- | --- | --- | --- | --- | --- | --- | --- | --- | --- | --- | --- | --- |
|  |  | estimate | std.error | estimate | std.error | estimate | std.error | estimate | std.error | estimate | std.error |  |  |
| **Non-communicable diseases** | ARIMA (0,1,2) with drift | - | - | - | - | 1.11 | 0.18 | 0.31 | 0.18 | -0.44 | 0.08 | 3.31 | 0.75 |
| Neoplasms | ARIMA (2,1,0) with drift | 1.05 | 0.16 | -0.42 | 0.16 | - | - | - | - | -0.13 | 0.04 | 1.80 | 0.12 |
| Cardiovascular diseases | ARIMA (0,2,2) | - | - | - | - | -0.09 | 0.16 | -0.60 | 0.15 | - | - | 1.85 | 0.28 |
| Chronic respiratory diseases | ARIMA (0,2,0) | - | - | - | - | - | - | - | - | - | - | 9.28 | 0.47 |
| Diabetes and kidney diseases | ARIMA (1,1,1) with drift | 0.39 | 0.19 | 0.73 | 0.14 | - | - | - | - | -0.03 | 0.01 | 6.61 | 0.15 |

**Table S2.** The orders and coefficients of ARIMA models for the mortality rates of NCDs in male

| VAL | ARIMA (p, d, q) | AR (1) | | AR (2) | | MA (1) | | MA (2) | | Drift | | MAPE | LB |
| --- | --- | --- | --- | --- | --- | --- | --- | --- | --- | --- | --- | --- | --- |
|  |  | estimate | std.error | estimate | std.error | estimate | std.error | estimate | std.error | estimate | std.error |  |  |
| **Non-communicable diseases** | ARIMA (0,1,2) with drift | - | - | - | - | -0.05 | 0.18 | -0.54 | 0.17 | - | - | 5.79 | 0.06 |
| Neoplasms | ARIMA (0,1,2) with drift | - | - | - | - | 1.26 | 0.16 | 0.80 | 0.18 | -0.14 | 0.03 | 3.60 | 0.34 |
| Cardiovascular diseases | ARIMA (0,2,1) | - | - | - | - | 0.47 | 0.16 | - | - | - | - | 2.28 | 0.02 |
| Chronic respiratory diseases | ARIMA (0,2,1) | - | - | - | - | 0.40 | 0.21 | - | - | - | - | 8.38 | 0.54 |
| Diabetes and kidney diseases | ARIMA (0,1,1) with drift | 0.82 | 0.14 | - | - | - | - | - | - | -0.04 | 0.01 | 9.38 | 0.28 |

**Table S3.** The orders and coefficients of ARIMA models for the mortality rates of NCDs in female

| VAL | ARIMA (p, d, q) | AR (1) | | AR (2) | | MA (1) | | MA (2) | | Drift | | MAPE | LB |
| --- | --- | --- | --- | --- | --- | --- | --- | --- | --- | --- | --- | --- | --- |
|  |  | estimate | std.error | estimate | std.error | estimate | std.error | estimate | std.error | estimate | std.error |  |  |
| Non-communicable diseases | ARIMA (0,1,1) with drift | - | - | - | - | 0.81 | 0.18 | - | - | -0.39 | 0.11 | 3.40 | 0.47 |
| Neoplasms | ARIMA (1,1,0) | 0.76 | 0.11 | - | - | - | - | - | - | - | - | 4.12 | 0.76 |
| Cardiovascular diseases | ARIMA (0,1,1) with drift | - | - | - | - | 0.62 | 0.26 | - | - | -0.08 | 0.01 | 3.45 | 0.35 |
| Chronic respiratory diseases | ARIMA (0,2,1) | - | - | -- | - | -0.48 | 0.17 | - | - | - | - | 11.37 | 0.65 |
| Diabetes and kidney diseases | ARIMA (0,1,1) with drift | 0.98 | 0.39 | - | - | - | - | - | - | -0.03 | 0.01 | 5.95 | 0.27 |

*ARIMA: Autoregressive Integrated Moving Average model
