# Supplementary material for: Trends in mortality and causes of death among Chinese adolescents aged 10–19 years from 1990 to 2019
Source: Front Public Health. 2023 Feb 7;11:1075858. doi: 10.3389/fpubh.2023.1075858 (PMC9941149; doi:10.3389/fpubh.2023.1075858)
Supplement: Supplementary file 1 [file Data_Sheet_1.ZIP › supplement-xiu/Supplement 4.docx]

**Table S1.** The orders and coefficients of ARIMA models for 118 causes of death, both sexes

| **VAL** | **ARIMA (p, d, q)** | **AR (1)** | | **AR (2)** | | **AR(3)** | | **MA (1)** | | **MA (2)** | | **Drift** | | **Intercept** | | **MAPE** | **LB** |
| --- | --- | --- | --- | --- | --- | --- | --- | --- | --- | --- | --- | --- | --- | --- | --- | --- | --- |
|  |  | **estimate** | **std.error** | **estimate** | **std.error** | **Estimate** | **Std.error** | **estimate** | **std.error** | **estimate** | **std.error** | **estimate** | **std.error** | **estimate** | **std.error** |  |  |
| Road injuries | ARIMA(1,2,1) | -0.15 | 0.20 | - | - |  |  | 1.00 | 0.11 | - | - | - | - | - | - | 0.28 | 0.22 |
| Drowning | ARIMA(0,2,0) | - | - | - | - |  |  | - | - | - | - | - | - | - | - | 0.21 | 0.44 |
| Self-harm | ARIMA(0,1,2) with drift | - | - | - | - |  |  | 1.15 | 0.18 | 0.46 | 0.15 | -0.07 | 0.01 | - | - | 0.70 | 0.68 |
| Leukemia | ARIMA(0,2,1) | - | - | - | - |  |  | 0.42 | 0.23 | - | - | - | - | - | - | 0.35 | 0.34 |
| Congenital birth defects | ARIMA(1,1,1) with drift | 0.70 | 0.14 | - | - |  |  | 0.52 | 0.18 | - | - | -0.04 | 0.02 | - | - | 0.51 | 0.06 |
| Falls | ARIMA(1,1,1) with drift | 0.76 | 0.12 | - | - |  |  | 0.50 | 0.15 | - | - | -0.03 | 0.01 | - | - | 0.22 | 0.36 |
| Other malignant neoplasms | ARIMA(0,2,1) | - | - | - | - |  |  | 0.74 | 0.17 | - | - | - | - | - | - | 0.37 | 0.74 |
| Brain and central nervous system cancer | ARIMA(0,2,1) | - | - | - | - |  |  | 0.48 | 0.16 | - | - | - | - | - | - | 0.33 | 0.32 |
| Stroke | ARIMA(0,2,0) | - | - | - | - |  |  | - | - | - | - | - | - | - | - | 0.43 | 0.20 |
| Poisonings | ARIMA(0,2,0) | - | - | - | - |  |  | - | - | - | - | - | - | - | - | 0.36 | 0.37 |
| Exposure to mechanical  forces | ARIMA(0.2,0) | - | - | - | - |  |  | - | - | - | - | - | - | - | - | 0.46 | 0.70 |
| Ischemic heart disease | ARIMA(2,1,0) with drift | 1.21 | 0.16 | -0.41 | 0.16 |  |  | - | - | - | - | -0.04 | 0.02 | - | - | 0.27 | 0.07 |
| Interpersonal violence | ARIMA(0,2,0) | - | - | - | - |  |  | - | - | - | - | - | - | - | - | 2.03 | 0.55 |
| Idiopathic epilepsy | ARIMA(0,2,1) | - | - | - | - |  |  | 0.76 | 0.20 | - | - | - | - | - | - | 0.42 | 0.52 |
| Lower respiratory infections | ARIMA(1,1,1) with drift | 0.64 | 0.16 | - | - |  |  | 0.56 | 0.18 | - | - | -0.07 | 0.02 | - | - | 0.46 | 0.39 |
| Other unintentional injuries | ARIMA(0,2,1) | - | - | - | - |  |  | 0.59 | 0.16 | - | - | - | - | - | - | 1.38 | 0.84 |
| Chronic kidney disease | ARIMA(0,2,1) | - | - | - | - |  |  | 0.54 | 0.15 | - | - | - | - | - | - | 1.03 | 0.30 |
| Non-Hodgkin lymphoma | ARIMA(1,1,1) | 0.82 | 0.10 | - | - |  |  | 0.71 | 0.12 | - | - | - | - | - | - | 0.89 | 0.28 |
| Other neurological disorders | ARIMA(1,1,1) | 0.59 | 0.16 | - | - |  |  | 0.79 | 0.19 | - | - | - | - | - | - | 0.38 | 0.68 |
| Cardiomyopathy and myocarditis | ARIMA(0,2,1) | - | - | - | - |  |  | 0.36 | 0.19 | - | - | - | - | - | - | 0.36 | 0.21 |
| Other musculoskeletal disorders | ARIMA(0,2,0) | - | - | - | - |  |  | - | - | - | - | - | - | - | - | 1.70 | 0.53 |
| Hemoglobinopathies and hemolytic anemias | ARIMA(0,2,0) | - | - | - | - |  |  | - | - | - | - | - | - | - | - | 1.26 | 0.36 |
| Other transport injuries | ARIMA(0,2,1) | - | - | - | - |  |  | -0.66 | 0.14 | - | - | - | - | - | - | 2.83 | 0.68 |
| HIV/AID | ARIMA(2,1,0) | 0.36 | 0.16 | 0.48 | 0.16 |  |  | - | - | - | - | - | - | - | - | 0.86 | 0.32 |
| Meningitis | ARIMA(0,1,2) with drift | - | - | - | - |  |  | 0.99 | 0.18 | 0.58 | 0.26 | -0.07 | 0.01 | - | - | 0.61 | 0.51 |
| Foreign body | ARIMA(1,1,1) | 0.70 | 0.14 | - | - |  |  | 0.52 | 0.18 | - | - | - | - | - | - | 2.52 | 0.48 |
| Endocrine, metabolic, blood, and immune disorders | ARIMA(1,1,1) with drift | 0.60 | 0.18 | - | - |  |  | 0.50 | 0.22 | - | - | -0.03 | 0.01 | - | - | 0.38 | 0.21 |
| Liver cancer | ARIMA(1,1,0) | 0.91 | 0.06 | - | - |  |  | - | - | -- | - | -- | - | - | - | 0.84 | 0.26 |
| Tuberculosis | ARIMA(0,2,0) | - | - | - | - |  |  | - | - | - | - | - | - | - | - | 0.86 | 0.75 |
| Drug use disorders | ARIMA(1,1,0) | 0.92 | 0.06 | - | - |  |  | - | - | - | - | - | - | - | - | 4.44 | 0.42 |
| Other cardiovascular and circulatory diseases | ARIMA(1,1,1) with drift | 0.53 | 0.17 | - | - |  |  | 0.70 | 0.16 | - | - | -0.04 | 0.01 | - | - | 1.74 | 0.41 |
| Tracheal, bronchus, and lung cancer | ARIMA(1,1,1) | 0.89 | 0.08 | - | - |  |  | 0.39 | 0.18 | - | - | - | - | - | - | 1.27 | 0.71 |
| Fire, heat, and hot substances | ARIMA(1,1,0) with drift | 0.43 | 0.17 | - | - |  |  | - | -- | - | - | -0.06 | 0.01 | - | - | 0.42 | 0.22 |
| Cirrhosis and other chronic liver diseases | ARIMA(0,2,0) | - | - | - | - |  |  | - | - | - | - | - | - | - | - | 1.99 | 0.12 |
| Typhoid and paratyphoid | ARIMA(1,1,0) with drift | 0.64 | 0.16 | - | - | - | - | - | - | - | - | -0.06 | 0.01 | - | - | 0.22 | 0.87 |
| Colon and rectum cancer | ARIMA(1,1,1) with drift | 0.64 | 0.17 | - | - |  |  | 0.65 | 0.23 | - | - | -0.03 | 0.01 | - | - | 1.01 | 0.72 |
| Encephalitis | ARIMA(0,2,0) | - | - | - | - |  |  | - | - | - | - | - | - | - | - | 2.57 | 0.55 |
| Rheumatic heart disease | ARIMA(1,1,1) with drift | 0.59 | 0.18 | - | - |  |  | 0.61 | 0.23 | - | - | -0.09 | 0.01 | - | - | 0.74 | 0.17 |
| Diabetes mellitus | ARIMA(0,2,1) | - | - | - | - |  |  | 0.41 | 0.21 | - | - | - | - | - | - | 1.57 | 0.47 |
| Alcohol use disorders | ARIMA(3,1,0) with drift | 1.06 | 0.14 | 0.33 | 0.23 | -0.60 | 0.14 | - | - | - | - | 0.03 | 0.01 | - | - | 0.34 | 0.19 |
| Chronic obstructive pulmonary disease | ARIMA(0,2,1) with drift | 1.29 | 0.16 | -0.49 | 0.16 | - | - | - | - | - | - | -0.08 | 0.01 | - | - | 2.35 | 0.29 |
| Stomach cancer | ARIMA(2,1,0) with drift | 1.56 | 0.12 | -0.70 | 0.12 | - | - | - | - | - | - | -0.05 | 0.02 | - | - | 0.38 | 0.90 |
| Adverse effects of medical treatment | ARIMA(2,1,0) with drift | 1.09 | 0.17 | -0.30 | 0.17 | - | - | - | - | - | - | -0.07 | 0.02 | - | - | 0.40 | 0.66 |
| Maternal disorders | ARIMA(1,1,0) with drift | 0.52 | 0.16 | - | - | - | - | - | - | - | - | -0.10 | 0.02 | - | - | 1.36 | 0.18 |
| Nasopharynx cancer | ARIMA(1,1,2) with drift | 0.70 | 0.15 | - | - | - | - | 0.98 | 0.17 | 0.68 | 0.24 | -0.06 | 0.02 | - | - | 1.09 | 0.37 |
| Kidney cancer | ARIMA(2,0,0) with non-zero mean | 1.75 | 0.08 | -0.85 | 0.08 | - | - | - | - | - | - | - | - | 4.52 | 0.05 | 2.28 | 0.12 |
| Acute glomerulonephritis | ARIMA(1,1,0) with drift | 0.74 | 0.12 | - | - | - | - | - | - | - | - | -0.08 | 0.01 | - | - | 0.39 | 0.59 |
| Paralytic ileus and intestinal obstruction | ARIMA(1,1,0) with drift | 0.77 | 0.11 | - | - | - | - | - | - | - | - | -0.05 | 0.02 | - | - | 1.51 | 0.54 |
| Diarrheal diseases | ARIMA(1,1,0) with drift | 0.71 | 0.13 | - | - | - | - | - | - | - | - | -0.10 | 0.02 | - | - | 1.48 | 0.21 |
| Other chronic respiratory diseases | ARIMA(0,2,0) | - | - | - | - | - | - | - | - | - | - | - | - | - | - | 0.94 | 0.62 |
| Upper digestive system diseases | ARIMA(1,1,0) with drift | 0.70 | 0.12 | - | - | - | - | - | - | - | - | -0.07 | 0.01 | - | - | 0.86 | 0.26 |
| Ovarian cancer | ARIMA(1,1,0) | 0.86 | 0.08 | - | - | - | - | - | - | - | - | - | - | - | - | 1.32 | 0.51 |
| Hypertensive heart disease | ARIMA(1,1,0) with drift | 0.83 | 0.10 | - | - | - | - | - | - | - | - | -0.05 | 0.01 | - | - | 2.11 | 0.30 |
| Motor neuron disease | ARIMA(1,1,0) | 0.88 | 0.07 | - | - | - | - | - | - | - | - | - | - | - | - | 1.98 | 0.64 |
| Urinary diseases and male infertility | ARIMA(0,2,0) | - | - | - | - | - | - | - | - | - | - | - | - | - | - | 2.08 | 0.32 |
| Other unspecified infectious diseases | ARIMA(1,1,2) with drift | 0.63 | 017 | - | - | - | - | -0.61 | 0.15 | 0.74 | 0.18 | -0.06 | 0.02 | - | - | 4.49 | 0.59 |
| Other digestive diseases | ARIMA(1,1,0) | 0.94 | 0.05 | - | - | - | - | - | - | - | - | - | - | - | - | 1.36 | 0.29 |
| Animal contact | ARIMA(1,1,0) with drift | 0.82 | 0.10 | - | - | - | - | - | - | - | - | -0.08 | 0.03 | - | - | 1.15 | 0.64 |
| Hodgkin lymphoma | ARIMA(1,1,1) with drift | 0.79 | 0.12 | - | - | - | - | 0.45 | 0.17 | - | - | -0.06 | 0.02 | - | - | 1.70 | 0.23 |
| Asthma | ARIMA(0,2,0) | - | - | - | - | - | - | - | - | - | - | - | - | - | - | 1.20 | 0.69 |
| Environmental heat and cold exposure | ARIMA(1,1,0) with drift | 0.79 | 0.11 | - | - | - | - | - | - | - | - | -0.07 | 0.02 | - | - | 2.10 | 0.28 |
| Pancreatitis | ARIMA(1,1,0) with drift | 0.73 | 0.12 | - | - | - | - | - | - | - | - | -0.05 | 0.01 | - | - | 1.42 | 0.40 |
| Other neoplasms | ARIMA(0,2,0) | - | - | - | - | - | - | - | - | - | - | - | - | - | - | 2.29 | 0.88 |
| Protein-energy malnutrition | ARIMA(1,1,0) with drift | 0.59 | 0.16 | - | - | - | - | - | - | - | - | -0.09 | 0.02 | - | - | 2.00 | 0.80 |
| Invasive Non-typhoidal Salmonella (iNTS) | ARIMA(1,1,0) with drift | 0.77 | 0.11 | - | - | - | - | - | - | - | - | -0.05 | 0.01 | - | - | 0.41 | 0.62 |
| Rabies | ARIMA(2,1,0) | 1.45 | 0.15 | -0.56 | 0.15 | - | - | - | - | - | - | - | - | - | - | 5.55 | 0.21 |
| Testicular cancer | ARIMA(1,1,0) | 0.87 | 0.08 | - | - | - | - | - | - | - | - | - | - | - | - | 1.28 | 0.18 |
| Endocarditis | ARIMA(1,1,1) | 0.87 | 0.08 | - | - | - | - | 0.84 | 0.13 | - | - | - | - | - | - | 2.62 | 0.51 |
| Pancreatic cancer | ARIMA(1,1,1) | 0.77 | 0.12 | - | - | - | - | 0.55 | 0.22 | - | - | - | - | - | - | 1.78 | 0.73 |
| Lip and oral cavity cancer | ARIMA(1,1,1) with drift | 0.71 | 0.14 | - | - | - | - | 0.57 | 0.21 | - | - | -0.02 | 0.02 | - | - | 1.55 | 0.47 |
| Breast cancer | ARIMA(1,1,0) with drift | 0.86 | 0.09 | - | - | - | - | - | - | - | - | -0.03 | 0.02 | - | - | 2.12 | 0.15 |
| Exposure to forces of nature | ARIMA(1,1,0) | -0.69 | 0.13 | - | - | - | - | - | - | - | - | - | - | - | - | 1.03 | 0.38 |
| Malignant skin melanoma | ARIMA(1,1,0) | 0.95 | 0.04 | - | - | - | - | - | - | - | - | - | - | - | - | 1.76 | 0.46 |
| Appendicitis | ARIMA(1,1,0) with drift | 0.84 | 0.09 | - | - | - | - | - | - | - | - | -0.08 | 0.02 | - | - | 2.42 | 0.14 |
| Measles | ARIMA(0,1,2) | - | - | - | - | - | - | 1.28 | 0.16 | 0.60 | 0.16 | - | - | - | - | 34.26 | 0.32 |
| Thyroid cancer | ARIMA(1,1,1) with drift | 0.60 | 0.17 | - | - | - | - | 0.41 | 0.21 | - | - | -0.03 | 0.01 | - | - | 0.66 | 0.87 |
| Cervical cancer | ARIMA(1,1,1) with drift | 0.77 | 0.12 | - | - | - | - | 0.56 | 0.20 | - | - | -0.04 | 0.02 | - | - | 0.35 | 0.22 |
| Other nutritional deficiencies | ARIMA(1,1,0) | 0.86 | 0.09 | - | - | - | - | - | - | - | - | - | - | - | - | 0.18 | 0.91 |
| Executions and police conflict | ARIMA(1,2,0) | 0.37 | 0.19 | - | - | - | - | - | - | - | - | - | - | - | - | 3.24 | 0.88 |
| Acute hepatitis | ARIMA(1,1,0) with drift | 0.69 | 0.13 | - | - | - | - | - | - | - | - | 0.11 | 0.02 | - | - | 2.79 | 0.27 |
| Inflammatory bowel disease | ARIMA(0,2,0) | - | - | - | - | - | - | - | - | - | - | - | - | - | - | 4.63 | 0.24 |
| Aortic aneurysm | ARIMA(1,1,1) | 0.88 | 0.08 | - | - | - | - | 0.55 | 0.17 | - | - | - | - | - | - | 3.52 | 0.39 |
| Non-rheumatic valvular heart disease | ARIMA(1,1,1) with drift | 0.81 | 0.10 | - | - | - | - | 0.56 | 0.17 | - | - | -0.03 | 0.02 | - | - | 2.92 | 0.15 |
| Tetanus | ARIMA(0,2,0) | - | - | - | - | - | - | - | - | - | - | - | - | - | - | 3.24 | 0.41 |
| Rheumatoid arthritis | ARIMA(1,1,0) | 0.81 | 0.10 | - | - | - | - | - | - | - | - | - | - | - | - | 0.40 | 0.88 |
| Bacterial skin diseases | ARIMA(1,1,1) with drift | 0.73 | 0.14 | - | - | - | - | 0.70 | 0.19 | - | - | -0.07 | 0.03 | - | - | 2.56 | 0.21 |
| Gallbladder and biliary diseases | ARIMA(1,1,0) with drift | 0.83 | 0.10 | - | - | - | - | - | - | - | - | -0.06 | 0.02 | - | - | 4.22 | 0.30 |
| Interstitial lung disease and pulmonary sarcoidosis | ARIMA(1,2,0) | -0.30 | 0.18 | - | - | - | - | - | - | - | - | - | - | - | - | 0.85 | 0.38 |
| Sexually transmitted infections excluding HIV | ARIMA(1,1,0) with drift | 0.68 | 0.13 | - | - | - | - | - | - | - | - | -0.03 | 0.01 | - | - | 1.61 | 0.21 |
| Bladder cancer | ARIMA(1,2,0) | 0.25 | 0.19 | - | - | - | - | - | - | - | - | - | - | - | - | 5.72 | 0.44 |
| Upper respiratory infections | ARIMA(1,1,1) | 0.94 | 0.05 | - | - | - | - | 0.52 | 0.18 | - | - | - | - | - | - | 1.22 | 0.24 |
| Whooping cough | ARIMA(1,1,0) with drift | 0.71 | 0.14 | - | - | - | - | - | - | - | - | -0.11 | 0.04 | - | - | 2.17 | 0.49 |
| Varicella and herpes zoster | ARIMA(1,1,0) with drift | 0.81 | 0.10 | - | - | - | - | - | - | - | - | -0.09 | 0.02 | - | - | 1.95 | 0.50 |
| Other neglected tropical diseases | ARIMA(1,1,1) | 0.82 | 0.10 | - | - | - | - | 0.93 | 0.13 | - | - | - | - | - | - | 2.16 | 0.82 |
| Pneumoconiosis | ARIMA(1,1,1) with rift | 0.84 | 0.09 | - | - | - | - | 0.66 | 0.15 | - | - | -0.07 | 0.02 | - | - | 5.96 | 0.06 |
| Eating disorders | ARIMA(1,1,0) | 0.31 | 0.17 | - | - | - | - | - | - | - | - | - | - | - | - | 3.96 | 0.42 |
| Inguinal, femoral, and abdominal hernia | ARIMA(0,2,0) | - | - | - | - | - | - | - | - | - | - | - | - | - | - | 9.13 | 0.11 |
| Decubitus ulcer | ARIMA(0,2,0) | - | - | - | - | - | - | - | - | - | - | - | - | - | - | 12.90 | 0.17 |
| Multiple sclerosis | ARIMA(3,1,1) | 0.68 | 0.12 | 0.78 | 0.11 | -0.75 | 0.11 | 0.63 | 0.18 | - | - | - | - | - | - | 6.12 | 0.30 |
| Other skin and subcutaneous diseases | ARIMA(0,2,1) | - | - | - | - | - | - | 0.46 | 0.19 | - | - | - | - | - | - | 6.58 | 0.75 |
| Other intestinal infectious diseases | ARIMA(1,1,1) with drift | 0.51 | 0.20 | - | - | - | - | 0.56 | 0.24 | - | - | -0.07 | 0.02 | - | - | 4.65 | 0.71 |
| Gynecological diseases | Arima(1,1,0) | 0.86 | 0.09 | - | - | - | - | - | - | - | - | - | - | - | - | 10.00 | 0.53 |
| Vascular intestinal disorders | ARIMA(0,2,0) | - | - | - | - | - | - | - | - | - | - | - | - | - | - | 37.48 | 0.33 |
| Intestinal nematode infections | ARIMA(2,1,0) with drift | 1.14 | 0.16 | -0.43 | 0.16 | - | - | - | - | - | - | -0.09 | 0.02 | - | - | 11.22 | 0.07 |
| Schistosomiasis | ARIMA(1,1,0) with drift | 0.75 | 0.12 | - | - | - | - | - | - | - | - | 0.12 | 0.02 | - | - | 68.93 | 0.71 |
| Diphtheria | ARIMA(2,1,0) with drift | 1.12 | 0.13 | -0.64 | 0.13 | - | - | - | - | - | - | -0.09 | 0.01 | - | - | 26.69 | 0.63 |
| Cystic echinococcosis | ARIMA(0,1,0) | - | - | - | - | - | - | - | - | - | - | - | - | - | - | 12.84 | 0.99 |
| Cysticercosis | ARIMA(1,1,1) with drift | 0.61 | 0.17 | - | - | - | - | 0.45 | 0.21 | - | - | -0.08 | 0.02 | - | - | 4.33 | 0.25 |
| Dengue | ARIMA(1,1,0) with drift | 0.60 | 0.15 | - | - | - | - | - | - | - | - | -0.07 | 0.03 | - | - | 5.65 | 0.14 |
| Otitis media | - | - | - | - | - | - | - | - | - | - | - | - | - | - | - | - | - |
| Conflict and terrorism | - | - | - | - | - | - | - | - | - | - | - | - | - | - | - | - | - |
| Malaria | - | - | - | - | - | - | - | - | - | - | - | - | - | - | - | - | - |
| African trypanosomiasis | - | - | - | - | - | - | - | - | - | - | - | - | - | - | - | - | - |
| Chagas disease | - | - | - | - | - | - | - | - | - | - | - | - | - | - | - | - | - |
| Ebola | - | - | - | - | - | - | - | - | - | - | - | - | - | - | - | - | - |
| Leishmaniasis | - | - | - | - | - | - | - | - | - | - | - | - | - | - | - | - | - |
| Yellow fever | - | - | - | - | - | - | - | - | - | - | - | - | - | - | - | - | - |
| Zika virus | - | - | - | - | - | - | - | - | - | - | - | - | - | - | - | - | - |

**Table S2.** The orders and coefficients of ARIMA models for 118 causes of death, male

| **VAL** | **ARIMA (p, d, q)** | **AR (1)** | | **AR (2)** | | **AR(3)** | | **MA (1)** | | **MA (2)** | | **Drift** | | **Intercept** | | **MAPE** | **LB** |
| --- | --- | --- | --- | --- | --- | --- | --- | --- | --- | --- | --- | --- | --- | --- | --- | --- | --- |
|  |  | **estimate** | **std.error** | **estimate** | **std.error** | **Estimate** | **Std.error** | **estimate** | **std.error** | **estimate** | **std.error** | **estimate** | **std.error** | **estimate** | **std.error** |  |  |
| Road injuries | ARIMA(1,2,0) | 0.51 | 0.16 | - | - | - | - | - | - | - | - | - | - | - | - | 0.60 | 0.10 |
| Drowning | ARIMA(0,2,0) | - | - | - | - |  |  | - | - | - | - | - | - | - | - | 0.24 | 0.70 |
| Self-harm | ARIMA(1,1,0) with drift | 0.76 | 0.11 | - | - | - | - | - | - | - | - | -0.05 | 0.02 | - | - | 1.32 | 0.18 |
| Leukemia | ARIMA(0,2,1) | - | - | - | - | - | - | 0.89 | 0.12 | - | - | - | - | - | - | 0.64 | 0.64 |
| Congenital birth defects | ARIMA(1,1,1) with drift | 0.75 | 0.13 | - | - | - | - | 0.37 | 0.18 | - | - | -0.04 | 0.01 | - | - | 0.16 | 0.06 |
| Falls | ARIMA(1,1,2) with drift | 0.62 | 0.16 | - | - | - | - | 1.04 | 0.20 | 0.57 | 0.18 | -0.03 | 0.01 | - | - | 0.19 | 0.86 |
| Other malignant neoplasms | ARIMA(0,2,1) | - | - | - | - | - | - | 0.44 | 0.16 | - | - | - | - | - | - | 1.12 | 0.62 |
| Brain and central nervous system cancer | ARIMA(1,1,0) | 0.90 | 0.07 | - | - | - | - | - | - |  | - | - | - | - | - | 0.56 | 0.33 |
| Stroke | ARIMA(1,1,0) with drift | 0.77 | 0.11 | - | - | - | - | - | - | - | - | -0.04 | 0.01 | - | - | 0.12 | 0.08 |
| Poisonings | ARIMA(0,2,0) | - | - | - | - |  |  | - | - | - | - | - | - | - | - | 0.61 | 0.38 |
| Exposure to mechanical  forces | ARIMA(0.2,0) | - | - | - | - |  |  | - | - | - | - | - | - | - | - | 0.81 | 0.53 |
| Ischemic heart disease | ARIMA(1,1,1) | 0.79 | 0.12 | - | - | - | - | 0.45 | 0.22 | - | - | - | - | - | - | 0.26 | 0.16 |
| Interpersonal violence | ARIMA(0,2,0) | - | - | - | - |  |  | - | - | - | - | - | - | - | - | 2.45 | 0.35 |
| Idiopathic epilepsy | ARIMA(1,1,1) with drift | 0.66 | 0.16 | - | - | - | - | 0.59 | 0.22 | - | - | -0.05 | 0.01 | - | - | 0.51 | 0.23 |
| Lower respiratory infections | ARIMA(1,1,1) with drift | 0.64 | 0.16 | - | - | - | - | 0.54 | 0.19 | - | - | -0.07 | 0.01 | - | - | 0.55 | 0.13 |
| Other unintentional injuries | ARIMA(1,1,1) | 0.89 | 0.08 | - | - | - | - | 0.63 | 0.15 | - | - | - | - | - | - | 0.72 | 0.47 |
| Chronic kidney disease | ARIMA(1,1,1) with drift | 0.82 | 0.10 | - | - | - | - | 0.38 | 0.17 | - | - | -0.05 | 0.02 | - | - | 1.27 | 0.12 |
| Non-Hodgkin lymphoma | ARIMA(1,1,1) with drift | 0.53 | 0.15 | - | - | - | - | 0.94 | 0.17 | - | - | -0.03 | 0.01 | - | - | 0.46 | 0.81 |
| Other neurological disorders | ARIMA(1,1,1) | 0.71 | 0.13 | - | - | - | - | 0.54 | 0.15 | - | - | - | - | - | - | 0.17 | 0.84 |
| Cardiomyopathy and myocarditis | ARIMA(0,2,1) | - | - | - | - | - | - | 0.70 | 0.17 | - | - | - | - | - | - | 0.27 | 0.71 |
| Other musculoskeletal disorders | ARIMA(1,1,0) with drift | 0.70 | 0.12 | - | - | - | - | - | - | - | - | -0.03 | 0.01 | - | - | 0.26 | 0.27 |
| Hemoglobinopathies and hemolytic anemias | ARIMA(0,2,1) | - | - | - | - | - | - | 0.44 | 0.17 | - | - | - | - | - | - | 1.52 | 0.90 |
| Other transport injuries | ARIMA(0,2,1) | - | - | - | - | - | - | -0.64 | 0.14 | - | - | - | - | - | - | 3.28 | 0.72 |
| HIV/AIDS | ARIMA(0,2,2) | - | - | - | - | - | - | -1.06 | 0.14 | 0.82 | 0.16 | - | - | - | - | 0.40 | 0.68 |
| Meningitis | ARIMA(1,1,1) with drift | 0.44 | 0.22 | - | - | - | - | 0.51 | 0.24 | - | - | -0.07 | 0.01 | - | - | 0.17 | 0.71 |
| Foreign body | ARIMA(1,1,1) | - | - | - | - | - | - | 1.31 | 0.22 | 0.81 | 0.23 | -0.02 | 0.01 | - | - | 2.16 | 0.73 |
| Endocrine, metabolic, blood, and immune disorders | ARIMA(1,1,1) with drift | 0.75 | 0.11 | - | - | - | - | - | - | - | - | -0.03 | 0.01 | - | - | 0.29 | 0.17 |
| Liver cancer | ARIMA(1,1,0) | 0.91 | 0.06 | - | - | - | - | - | - | - | - | - | - | - | - | 1.27 | 0.67 |
| Tuberculosis | ARIMA(1,1,0) with drift | 0.71 | 0.12 | - | - | - | - | - | - | - | - | -0.09 | 0.02 | - | - | 0.68 | 0.67 |
| Drug use disorders | ARIMA(1,1,0) | 0.91 | 0.06 | - | - | - | - | - | - | - | - | - | - | - | - | 5.20 | 0.79 |
| Other cardiovascular and circulatory diseases | ARIMA(0,2,1) | - | - | - | - | - | - | 0.54 | 0.15 | - | - | - | - | - | - | 0.97 | 0.14 |
| Tracheal, bronchus, and lung cancer | ARIMA(1,1,0) with drift | 0.87 | 0.08 | - | - | - | - | - | - | - | - | -0.03 | 0.02 | - | - | 2.31 | 0.05 |
| Fire, heat, and hot substances | ARIMA(1,1,0) with drift | 0.44 | 0.16 | - | - | - | - | - | - | - | - | -0.06 | 0.01 | - | - | 0.38 | 0.69 |
| Cirrhosis and other chronic liver diseases | ARIMA(0,2,0) | - | - | - | - |  |  | - | - | - | - | - | - | - | - | 2.46 | 0.27 |
| Typhoid and paratyphoid | ARIMA(1,1,0) with drift | 0.71 | 0.15 | - | - | - | - | - | - | - | - | -0.06 | 0.01 | - | - | 0.53 | 0.59 |
| Colon and rectum cancer | ARIMA(1,1,0) | 0.87 | 0.08 | - | - | - | - | - | - | - | - | - | - | - | - | 1.61 | 0.50 |
| Encephalitis | ARIMA(1,1,0) | 0.80 | 0.06 | - | - | - | - | - | - | - | - | - | - | - | - | 1.48 | 0.28 |
| Rheumatic heart disease | ARIMA(4,1,0) with drift | 1.81 | 0.16 | -1.72 | 0.29 | 1.21 | 0.29 | - | - | - | - | -0.08 | 0.01 | - | - | 0.32 | 0.28 |
| Diabetes mellitus | ARIMA(1,1,1) | 0.88 | 0.08 | - | - | - | - | 0.55 | 0.17 | - | - | - | - | - | - | 1.00 | 0.94 |
| Alcohol use disorders | ARIMA(1,3,2) | 0.02 | 0.32 | - | - | - | - | -0.53 | 0.25 | 0.60 | 0.39 | - | - | - | - | 2.03 | 0.05 |
| Chronic obstructive pulmonary disease | ARIMA(2,1,0) with drift | 1.21 | 0.16 | -0.44 | 0.17 | - | - | - | - | - | - | -0.08 | 0.01 | - | - | 1.82 | 0.17 |
| Stomach cancer | ARIMA(2,1,1) with drift | 1.79 | 0.10 | -0.90 | 0.08 | - | - | -0.75 | 0.30 | - | - | -0.04 | 0.01 | - | - | 0.59 | 0.22 |
| Adverse effects of medical treatment | ARIMA(2,1,0) with drift | 1.08 | 0.17 | -0.32 | 0.17 | - | - | - | - | - | - | -0.06 | 0.01 | - | - | 0.53 | 0.73 |
| Maternal disorders | - | - | - | - | - | - | - | - | - | - | - | - | - | - | - | - | - |
| Nasopharynx cancer | ARIMA(1,1,0) | 0.96 | 0.03 | - | - | - | - | - | - | - | - | - | - | - | - | 2.90 | 0.35 |
| Kidney cancer | ARIMA(1,1,2) | 1.65 | 0.12 | -0.76 | 0.12 | - | - | 0.55 | 0.27 | - | - | - | - | 50.17 | 2.82 | 0.96 | 0.63 |
| Acute glomerulonephritis | ARIMA(1,1,0) with drift | 0.79 | 0.11 | - | - | - | - | - | - | - | - | -0.08 | 0.02 | - | - | 0.48 | 0.36 |
| Paralytic ileus and intestinal obstruction | ARIMA(1,1,0) with drift | 0.79 | 0.11 | - | - | - | - | - | - | - | - | -0.05 | 0.02 | - | - | 2.01 | 0.45 |
| Diarrheal diseases | ARIMA(1,1,1) with drift | 0.29 | 0.28 | - | - | - | - | 0.79 | 0.30 | - | - | -0.10 | 0.01 | - | - | 2.69 | 0.11 |
| Other chronic respiratory diseases | ARIMA(0,2,0) | - | - | - | - | - | - | - | - | - | - | - | - | - | - | 1.36 | 0.91 |
| Upper digestive system diseases | ARIMA(1,1,0) with drift | 0.73 | 0.12 | - | - | - | - | - | - | - | - | -0.07 | 0.02 | - | - | 1.15 | 0.34 |
| Ovarian cancer | - | - | - | - | - | - | - | - | - | - | - | - | - | - | - | - | - |
| Hypertensive heart disease | ARIMA(0,2,0) | - | - | - | - | - | - | - | - | - | - | - | - | - | - | 2.57 | 0.97 |
| Motor neuron disease | ARIMA(1,1,0) | 0.87 | 0.08 | - | - | - | - | - | - | - | - | - | - | - | - | 2.32 | 0.75 |
| Urinary diseases and male infertility | ARIMA(2,1,0) with drift | 1.21 | 0.16 | 0.40 | 0.16 | - | - | - | - | - | - | -0.05 | 0.02 | - | - | 0.42 | 0.05 |
| Other unspecified infectious diseases | ARIMA(2,1,0) with drift | 0.27 | 0.17 | 0.35 | 0.17 | - | - | - | - | - | - | -0.07 | 0.02 | - | - | 5.42 | 0.23 |
| Other digestive diseases | ARIMA(1,1,0) | 0.95 | 0.04 | - | - | - | - | - | - | - | - | - | - | - | - | 1.78 | 0.53 |
| Animal contact | ARIMA(1,1,0) with drift | 0.85 | 0.09 | - | - | - | - | - | - | - | - | -0.08 | 0.03 | - | - | 1.77 | 0.65 |
| Hodgkin lymphoma | ARIMA(1,1,1) with drift | 0.75 | 0.13 | - | - | - | - | 0.41 | 0.17 | - | - | -0.07 | 0.02 | - | - | 2.04 | 0.21 |
| Asthma | ARIMA(0,2,0) | - | - | - | - | - | - | - | - | - | - | - | - | - | - | 1.51 | 0.52 |
| Environmental heat and cold exposure | ARIMA(1,1,0) with drift | 0.78 | 0.11 | - | - | - | - | - | - | - | - | -0.08 | 0.02 | - | - | 2.37 | 0.15 |
| Pancreatitis | ARIMA(1,1,0) with drift | 0.72 | 0.12 | - | - | - | - | - | - | - | - | -0.05 | 0.01 | - | - | 2.21 | 0.30 |
| Other neoplasms | ARIMA(0,2,1) | - | - | - | - | - | - | 0.40 | 0.21 | - | - | - | - | - | - | 1.71 | 0.68 |
| Protein-energy malnutrition | ARIMA(0,1,1) with drift | - | - | - | - | - | - | 0.85 | 0.19 | - | - | -0.08 | 0.01 | - | - | 2.84 | 0.10 |
| Invasive Non-typhoidal Salmonella (iNTS) | ARIMA(1,1,0) with drift | 0.74 | 0.12 | - | - | - | - | - | - | - | - | -0.05 | 0.01 | - | - | 0.66 | 0.66 |
| Rabies | ARIMA(2,0,2) with non-zero mean | 1.89 | 0.04 | -0.97 | 0.04 | - | - | -0.09 | 0.22 | 0.07 | 0.25 | - | - | 4.37 | 0.11 | 4.58 | 0.10 |
| Testicular cancer | ARIMA(1,1,0) | 0.87 | 0.08 | - | - | - | - | - | - | - | - | - | - | - | - | 1.37 | 0.21 |
| Endocarditis | ARIMA(1,1,2) | 0.86 | 0.08 | - | - | - | - | 1.00 | 0.20 | 0.34 | 0.19 | - | - | - | - | 0.55 | 0.90 |
| Pancreatic cancer | ARIMA(1,1,1) | 0.70 | 0.14 | - | - | - | - | 0.59 | 0.21 | - | - | - | - | - | - | 1.96 | 0.50 |
| Lip and oral cavity cancer | ARIMA(2,1,0) with drift | 1.19 | 0.17 | -0.41 | 0.18 | - | - | - | - | - | - | -0.02 | 0.01 | - | - | 0.99 | 0.38 |
| Breast cancer | ARIMA(1,1,2) | 0.89 | 0.10 | - | - | - | - | 1.26 | 0.16 | 0.87 | 0.25 | - | - | - | - | 1.92 | 0.29 |
| Exposure to forces of nature*(白噪声序列) | - | - | - | - | - | - | - | - | - | - | - | - | - | - | - | - | - |
| Malignant skin melanoma | ARIMA(1,1,0) | 0.93 | 0.06 | - | - | - | - | - | - | - | - | - | - | - | - | 2.99 | 0.21 |
| Appendicitis | ARIMA(1,1,0) with drift | 0.81 | 0.10 | - | - | - | - | - | - | - | - | -0.08 | 0.03 | - | - | 3.16 | 0.37 |
| Measles | ARIMA(2,1,0) | 1.12 | 0.15 | -0.52 | 0.15 | - | - | - | - | - | - | - | - | - | - | 15.93 | 0.20 |
| Thyroid cancer | ARIMA(2,1,0) with drift | 1.04 | 0.16 | -0.43 | 0.16 | - | - | - | - | - | - | -0.02 | 0.01 | - | - | 2.12 | 0.59 |
| Cervical cancer | - | - | - | - | - | - | - | - | - | - | - | - | - | - | - | - | - |
| Other nutritional deficiencies | ARIMA(1,1,0) | 0.90 | 0.08 | - | - | - | - | - | - | - | - | - | - | - | - | 1.96 | 0.70 |
| Executions and police conflict | ARIMA(2,1,0) | 1.32 | 0.19 | -0.38 | 0.19 | - | - | - | - | - | - | - | - | - | - | 3.71 | 0.65 |
| Acute hepatitis | ARIMA(1,1,0) with drift | 0.69 | 0.13 | - | - | - | - | - | - | - | - | -0.11 | 0.02 | - | - | 4.32 | 0.39 |
| Inflammatory bowel disease | ARIMA(0,2,0) | - | - | - | - | - | - | - | - | - | - | - | - | - | - | 5.46 | 0.38 |
| Aortic aneurysm | ARIMA(1,1,1) | 0.85 | 0.09 | - | - | - | - | 0.55 | 0.18 | - | - | - | - | - | - | 4.24 | 0.42 |
| Non-rheumatic valvular heart disease | ARIMA(2,1,0) with drift | 1.37 | 0.17 | -0.54 | 0.17 | - | - | - | - | - | - | -0.04 | 0.02 | - | - | 3.02 | 0.77 |
| Tetanus | ARIMA(0,2,0) | - | - | - | - | - | - | - | - | - | - | - | - | - | - | 5.49 | 0.81 |
| Rheumatoid arthritis | ARIMA(3,1,1) | 0.93 | 0.17 | 0.68 | 0.27 | -0.77 | 0.16 | -0.67 | 0.18 | - | - | - | - | - | - | 5.00 | 0.05 |
| Bacterial skin diseases | ARIMA(1,1,1) | 0.91 | 0.07 | - | - | - | - | 0.49 | 0.23 | - | - |  | - | - | - | 1.59 | 0.72 |
| Gallbladder and biliary diseases | ARIMA(1,1,0) with drift | 0.76 | 0.11 | - | - | - | - | - | - | - | - | -0.07 | 0.02 | - | - | 6.27 | 0.35 |
| Interstitial lung disease and pulmonary sarcoidosis | ARIMA(1,1,0) with drift | 0.61 | 0.15 | - | - | - | - | - | - | - | - | -0.04 | 0.01 | - | - | 1.60 | 0.29 |
| Sexually transmitted infections excluding HIV | ARIMA(1,1,0) | 0.88 | 0.08 | - | - | - | - | - | - | - | - | - | - | - | - | 1.43 | 0.48 |
| Bladder cancer | ARIMA(1,1,0) | 0.92 | 0.06 | - | - | - | - | - | - | - | - | - | - | - | - | 6.92 | 0.66 |
| Upper respiratory infections | ARIMA(1,1,1) | 0.95 | 0.04 | - | - | - | - | 0.38 | 0.19 | - | - | - | - | - | - | 2.10 | 0.53 |
| Whooping cough | ARIMA(2,1,0) with drift | 1.04 | 0.21 | -0.46 | 0.20 | - | - | - | - | - | - | -0.12 | 0.03 | - | - | 9.90 | 0.64 |
| Varicella and herpes zoster | ARIMA(1,1,0) with drift | 0.76 | 0.12 | - | - | - | - | - | - | - | - | -0.10 | 0.02 | - | - | 2.94 | 0.67 |
| Other neglected tropical diseases | ARIMA(2,1,0) | 1.28 | 0.16 | -0.42 | 0.16 | - | - | - | - | - | - | - | - | - | - | 0.80 | 0.91 |
| Pneumoconiosis | ARIMA(2,1,0) with drift | 1.40 | 0.21 | -0.65 | 0.20 | - | - | - | - | - | - | -0.09 | 0.02 | - | - | 5.20 | 0.63 |
| Eating disorders*（no suitable ARIMA model） | - | - | - | - | - | - | - | - | - | - | - | - | - | - | - | - | - |
| Inguinal, femoral, and abdominal hernia*（no suitable ARIMA model） | - | - | - | - | - | - | - | - | - | - | - | - | - | - | - | - | - |
| Decubitus ulcer | ARIMA(0,2,0) | - | - | - | - | - | - | - | - | - | - | - | - | - | - | 19.97 | 0.88 |
| Multiple sclerosis*( no suitable ARIMA model） | - | - | - | - | - | - | - | - | - | - | - | - | - | - | - | - | - |
| Other skin and subcutaneous diseases | ARIMA(0,2,1) | - | - | - | - | - | - | 0.32 | 0.18 | - | - | - | - | - | - | 19.28 | 0.95 |
| Other intestinal infectious diseases | ARIMA(0,2,0) | - | - | - | - | - | - | - | - | - | - | - | - | - | - | 2.00 | 0.47 |
| Gynecological diseases | - | - | - | - | - | - | - | - | - | - | - | - | - | - | - | - | - |
| Vascular intestinal disorders | ARIMA(0,2,0) | - | - | - | - | - | - | - | - | - | - | - | - | - | - | 0.24 | 0.45 |
| Intestinal nematode infections | ARIMA(2,1,0) with drift | 0.94 | 0.17 | -0.30 | 0.17 | - | - | - | - | - | - | -0.09 | 0.02 | - | - | 0.02 | 0.07 |
| Schistosomiasis | ARIMA(1,1,0) with drift | 0.88 | 0.09 | - | - | - | - | - | - | - | - | - | - | - | - | 0.14 | 0.96 |
| Diphtheria | ARIMA(0,1,1) with drift | - | - | - | - | - | - | 0.69 | 0.19 | - | - | -0.07 | 0.03 | - | - | 0.17 | 0.85 |
| Cystic echinococcosis | ARIMA(0,1,0) | - | - | - | - | - | - | - | - | - | - | - | - | - | - | 0.62 | 0.80 |
| Cysticercosis | - | - | - | - | - | - | - | - | - | - | - | - | - | - | - | - | - |
| Dengue | - | - | - | - | - | - | - | - | - | - | - | - | - | - | - | - | - |
| Otitis media | - | - | - | - | - | - | - | - | - | - | - | - | - | - | - | - | - |
| Conflict and terrorism | - | - | - | - | - | - | - | - | - | - | - | - | - | - | - | - | - |
| Malaria | - | - | - | - | - | - | - | - | - | - | - | - | - | - | - | - | - |
| African trypanosomiasis | - | - | - | - | - | - | - | - | - | - | - | - | - | - | - | - | - |
| Chagas disease | - | - | - | - | - | - | - | - | - | - | - | - | - | - | - | - | - |
| Ebola | - | - | - | - | - | - | - | - | - | - | - | - | - | - | - | - | - |
| Leishmaniasis | - | - | - | - | - | - | - | - | - | - | - | - | - | - | - | - | - |
| Yellow fever | - | - | - | - | - | - | - | - | - | - | - | - | - | - | - | - | - |
| Zika virus | - | - | - | - | - | - | - | - | - | - | - | - | - | - | - | - | - |

**Table S3.** The orders and coefficients of ARIMA models for 118 causes of death, female

| **VAL** | **ARIMA (p, d, q)** | **AR (1)** | | **AR (2)** | | **AR(3)** | | **MA (1)** | | **MA (2)** | | **Drift** | | **Intercept** | | **MAPE** | **LB** |
| --- | --- | --- | --- | --- | --- | --- | --- | --- | --- | --- | --- | --- | --- | --- | --- | --- | --- |
|  |  | **estimate** | **std.error** | **estimate** | **std.error** | **Estimate** | **Std.error** | **estimate** | **std.error** | **estimate** | **std.error** | **estimate** | **std.error** | **estimate** | **std.error** |  |  |
| Road injuries | ARIMA(0,2,0) | - | - | - | - | - | - | - | - | - | - | - | - | - | - | 0.78 | 0.72 |
| Drowning | ARIMA(1,1,0) with drift | 0.71 | 0.12 | - | - | - | - | - | - | - | - | -0.06 | 0.02 | - | - | 0.44 | 0.41 |
| Self-harm | ARIMA(0,1,1) with drift | - | - | - | - | - | - | 0.86 | 0.16 | - | - | -0.08 | 0.01 | - | - | 0.73 | 0.34 |
| Leukemia | ARIMA(0,2,0) | - | - | - | - | - | - | - | - | - | - | - | - | - | - | 0.55 | 0.85 |
| Congenital birth defects | ARIMA(0,2,0) | - | - | - | - | - | - | - | - | - | - | - | - | - | - | 0.47 | 0.30 |
| Falls | ARIMA(0,2,0) | - | - | - | - | - | - | - | - | - | - | - | - | - | - | 0.34 | 0.50 |
| Other malignant neoplasms | ARIMA(0,2,0) | - | - | - | - | - | - | - | - | - | - | - | - | - | - | 0.90 | 0.74 |
| Brain and central nervous system cancer | ARIMA(1,2,0) | 0.41 | 0.17 | - | - | - | - | - | - | - | - | - | - | - | - | 0.30 | 0.58 |
| Stroke | ARIMA(0,2,0) | - | - | - | - | - | - | - | - | - | - | - | - | - | - | 0.74 | 0.76 |
| Poisonings | ARIMA(0,2,0) | - | - | - | - | - | - | - | - | - | - | - | - | - | - | 0.41 | 0.81 |
| Exposure to mechanical  forces | ARIMA(0.2,0) | - | - | - | - | - | - | - | - | - | - | - | - | - | - | 1.53 | 0.33 |
| Ischemic heart disease | ARIMA(2,1,0) with drift | 1.16 | 0.17 | -0.36 | 0.17 | - | - | - | - | - | - | -0.04 | 0.02 | - | - | 1.12 | 0.93 |
| Interpersonal violence | ARIMA(0,2,0) | - | - | - | - | - | - | - | - | - | - | - | - | - | - | 1.57 | 0.66 |
| Idiopathic epilepsy | ARIMA(0,2,0) | - | - | - | - | - | - | - | - | - | - | - | - | - | - | 0.88 | 0.98 |
| Lower respiratory infections | ARIMA(1,1,0) with drift | 0.73 | 0.12 | - | - | - | - | - | - | - | - | - | - | - | - | 0.60 | 0.39 |
| Other unintentional injuries | ARIMA(0,2,0) | - | - | - | - | - | - | - | - | - | - | - | - | - | - | 0.97 | 0.25 |
| Chronic kidney disease | ARIMA(0,2,1) | - | - | - | - | - | - | 0.46 | 0.21 | - | - | - | - | - | - | 0.86 | 0.31 |
| Non-Hodgkin lymphoma | ARIMA(1,1,1) | 0.74 | 0.13 | - | - | - | - | 0.51 | 0.22 | - | - | - | - | - | - | 2.00 | 0.61 |
| Other neurological disorders | ARIMA(0,2,0) | - | - | - | - | - | - | - | - | - | - | - | - | - | - | 1.30 | 0.83 |
| Cardiomyopathy and myocarditis | ARIMA(0,2,0) | - | - | - | - | - | - | - | - | - | - | - | - | - | - | 0.59 | 0.77 |
| Other musculoskeletal disorders | ARIMA(0,2,0) | - | - | - | - | - | - | - | - | - | - | - | - | - | - | 1.95 | 0.71 |
| Hemoglobinopathies and hemolytic anemias | ARIMA(0,2,0) | - | - | - | - | - | - | - | - | - | - | - | - | - | - | 0.84 | 0.54 |
| Other transport injuries | ARIMA(0,2,1) | - | - | - | - | - | - | -0.67 | 0.16 | - | - | - | - | - | - | 2.88 | 0.59 |
| HIV/AIDS | ARIMA(1,1,0) with drift | 0.68 | 0.13 | - | - | - | - | - | - | - | - | 0.07 | 0.03 | - | - | 2.00 | 0.46 |
| Meningitis | ARIMA(1,1,0) with drift | 0.67 | 0.13 | - | - | - | - | - | - | - | - | -0.07 | 0.02 | - | - | 0.92 | 0.87 |
| Foreign body | ARIMA(1,1,0) | 0.76 | 0.11 | - | - | - | - | - | - | - | - | - | - | - | - | 2.82 | 0.51 |
| Endocrine, metabolic, blood, and immune disorders | ARIMA(1,1,0) with drift | 0.72 | 0.12 | - | - | - | - | - | - | - | - | -0.04 | 0.02 | - | - | 0.69 | 0.43 |
| Liver cancer | ARIMA(1,1,0) | 0.88 | 0.07 | - | - | - | - | - | - | - | - | - | - | - | - | 0.29 | 0.28 |
| Tuberculosis | ARIMA(0,2,0) | - | - | - | - | - | - | - | - | - | - | - | - | - | - | 0.55 | 0.63 |
| Drug use disorders | ARIMA(2,1,2) | -0.01 | 0.12 | 0.75 | 0.12 | - | - | 1.35 | 0.16 | 0.75 | 0.19 | - | - | - | - | 2.79 | 0.47 |
| Other cardiovascular and circulatory diseases | ARIMA(1,1,0) | 0.81 | 0.10 | - | - | - | - | - | - | - | - | - | - | - | - | 1.62 | 0.37 |
| Tracheal, bronchus, and lung cancer | ARIMA(1,1,1) | 0.84 | 0.09 | - | - | - | - | 0.47 | 0.22 | - | - | - | - | - | - | 1.07 | 0.99 |
| Fire, heat, and hot substances | ARIMA(1,1,0) with drift | 0.50 | 0.16 | - | - | - | - | - | - | - | - | -0.06 | 0.01 | - | - | 0.80 | 0.45 |
| Cirrhosis and other chronic liver diseases | ARIMA(0,2,0) | - | - | - | - | - | - | - | - | - | - | - | - | - | - | 1.94 | 0.22 |
| Typhoid and paratyphoid | ARIMA(0,1,1) with drift | - | - | - | - | - | - | 0.49 | 0.20 | - | - | -0.06 | 0.01 | - | - | 0.32 | 0.96 |
| Colon and rectum cancer | ARIMA(1,1,0) | 0.92 | 0.06 | - | - | - | - | - | - | - | - | - | - | - | - | 1.23 | 0.69 |
| Encephalitis | ARIMA(0,2,0) | - | - | - | - | - | - | - | - | - | - | - | - | - | - | 2.71 | 0.47 |
| Rheumatic heart disease | ARIMA(0,2,0) | - | - | - | - | - | - | - | - | - | - | - | - | - | - | 1.20 | 0.20 |
| Diabetes mellitus | ARIMA(0,2,0) | - | - | - | - | - | - | - | - | - | - | - | - | - | - | 2.63 | 0.73 |
| Alcohol use disorders | ARIMA(1,1,2) with drift | 0.68 | 0.15 | - | - | - | - | 0.61 | 0.16 | 0.63 | 0.15 | -0.06 | 0.02 | - | - | 0.80 | 0.47 |
| Chronic obstructive pulmonary disease | ARIMA(1,1,0) with drift | 0.79 | 0.11 | - | - | - | - | - | - | - | - | -0.09 | 0.02 | - | - | 1.91 | 0.46 |
| Stomach cancer | ARIMA(2,1,0) with drift | 1.45 | 0.14 | -0.61 | 0.13 | - | - | - | - | - | - | -0.05 | 0.02 | - | - | 0.21 | 0.21 |
| Adverse effects of medical treatment | ARIMA(1,1,0) with drift | 0.80 | 0.10 | - | - | - | - | - | - | - | - | -0.07 | 0.03 | - | - | 0.88 | 0.61 |
| Maternal disorders | ARIMA(1,1,0) with drift | 0.52 | 0.16 | - | - | - | - | - | - | - | - | -0.10 | 0.02 | - | - | 1.37 | 0.19 |
| Nasopharynx cancer | ARIMA(2,1,0) | 1.36 | 0.16 | -0.43 | 0.16 | - | - | - | - | - | - | - | - | - | - | 0.77 | 0.49 |
| Kidney cancer | ARIMA(1,1,0) | 0.71 | 0.12 | - | - | - | - | - | - | - | - | - | - | - | - | 2.37 | 0.71 |
| Acute glomerulonephritis | ARIMA(0,1,1) with drift | - | - | - | - | - | - | 0.87 | 0.14 | - | - | -0.08 | 0.01 | - | - | 0.91 | 0.25 |
| Paralytic ileus and intestinal obstruction | ARIMA(1,1,0) with drift | 0.66 | 0.13 | - | - | - | - | - | - | - | - | -0.06 | 0.02 | - | - | 1.16 | 0.61 |
| Diarrheal diseases | ARIMA(0,1,1) with drift | - | - | - | - | - | - | 0.84 | 0.15 | - | - | -0.10 | 0.01 | - | - | 1  .03 | 0.27 |
| Other chronic respiratory diseases | ARIMA(1,2,0) | -0.31 | 0.18 | - | - | - | - | - | - | - | - | - | - | - | - | 1.43 | 0.90 |
| Upper digestive system diseases | ARIMA(1,1,0) with drift | 0.60 | 0.14 | - | - | - | - | - | - | - | - | -0.07 | 0.01 | - | - | 0.85 | 0.43 |
| Ovarian cancer | ARIMA(1,1,0) | 0.86 | 0.08 | - | - | - | - | - | - | - | - | - | - | - | - | 1.28 | 0.44 |
| Hypertensive heart disease | ARIMA(1,1,1) with drift | 0.55 | 0.19 | - | - | - | - | 0.60 | 0.23 | - | - | -0.06 | 0.01 | - | - | 1.47 | 0.42 |
| Motor neuron disease | ARIMA(1,1,0) | 0.81 | 0.09 | - | - | - | - | - | - | - | - | - | - | - | - | 0.44 | 0.17 |
| Urinary diseases and male infertility | ARIMA(1,1,0) | 0.90 | 0.07 | - | - | - | - | - | - | - | - | - | - | - | - | 0.85 | 0.55 |
| Other unspecified infectious diseases | ARIMA(2,1,0) with drift | 0.32 | 0.16 | 0.39 | 0.16 | - | - | - | - | - | - | -0.05 | 0.02 | - | - | 3.17 | 0.51 |
| Other digestive diseases | ARIMA(1,1,0) | 0.90 | 0.06 | - | - | - | - | - | - | - | - | - | - | - | - | 1.26 | 0.75 |
| Animal contact | ARIMA(1,1,0) with drift | 0.66 | 0.13 | - | - | - | - | - | - | - | - | -0.08 | 0.02 | - | - | 1.51 | 0.63 |
| Hodgkin lymphoma | ARIMA(1,1,0) with drift | 0.80 | 0.11 | - | - | - | - | - | - | - | - | -0.06 | 0.02 | - | - | 2.85 | 0.54 |
| Asthma | ARIMA(0,2,0) | - | - | - | - | - | - | - | - | - | - | - | - | - | - | 1.77 | 0.68 |
| Environmental heat and cold exposure | ARIMA(1,1,0) with drift | 0.64 | 0.14 | - | - | - | - | - | - | - | - | -0.06 | 0.02 | - | - | 3.40 | 0.58 |
| Pancreatitis | ARIMA(1,1,0) with drift | 0.68 | 0.13 | - | - | - | - | - | - | - | - | -0.06 | 0.01 | - | - | 1.29 | 0.45 |
| Other neoplasms | ARIMA(0,2,0) | - | - | - | - | - | - | - | - | - | - | - | - | - | - | 3.35 | 0.94 |
| Protein-energy malnutrition | ARIMA(1,1,0) with drift | 0.61 | 0.15 | - | - | - | - | - | - | - | - | -0.10 | 0.03 | - | - | 2.54 | 0.38 |
| Invasive Non-typhoidal Salmonella (iNTS) | ARIMA(1,1,0) with drift | 0.64 | 0.14 | - | - | - | - | - | - | - | - | -0.05 | 0.02 | - | - | 0.41 | 0.40 |
| Rabies | ARIMA(0,2,0) | - | - | - | - | - | - | - | - | - | - | - | - | - | - | 9.35 | 0.93 |
| Testicular cancer | - | - | - | - | - | - | - | - | - | - | - | - | - | - | - | - | - |
| Endocarditis | ARIMA(1,1,1) with drift | 0.68 | 0.16 | - | - | - | - | 0.61 | 0.30 | - | - | -0.05 | 0.03 | - | - | 3.08 | 0.39 |
| Pancreatic cancer | ARIMA(1,1,1) | 0.85 | 0.09 | - | - | - | - | 0.54 | 0.17 | - | - | - | - | - | - | 3.11 | 0.42 |
| Lip and oral cavity cancer | ARIMA(1,1,0) with drift | 0.94 | 0.05 | - | - | - | - | - | - | - | - | - | - | - | - | 3.28 | 0.44 |
| Breast cancer | ARIMA(2,1,0) | 1.29 | 0.18 | 0.38 | 0.17 | - | - | - | - | - | - | - | - | - | - | 0.99 | 0.77 |
| Exposure to forces of nature*(白噪声序列) | - | - | - | - | - | - | - | - | - | - | - | - | - | - | - | - | - |
| Malignant skin melanoma | ARIMA(0,2,0) | - | - | - | - | - | - | - | - | - | - | - | - | - | - | 2.07 | 0.91 |
| Appendicitis | ARIMA(1,1,0) with drift | 0.80 | 0.11 | - | - | - | - | - | - | - | - | -0.08 | 0.02 | - | - | 2.89 | 0.64 |
| Measles | ARIMA(2,1,0) with drift | 1.09 | 0.14 | -0.57 | 0.14 | - | - | - | - | - | - | -0.13 | 0.07 | - | - | 35.86 | 0.12 |
| Thyroid cancer | ARIMA(1,1,0) with drift | 0.76 | 0.11 | - | - | - | - | - | - | - | - | -0.05 | 0.02 | - | - | 1.49 | 0.52 |
| Cervical cancer | ARIMA(2,1,0) with drift | 1.33 | 0.15 | -0.53 | 0.15 | - | - | - | - | - | - | -0.04 | 0.01 | - | - | 1.01 | 0.59 |
| Other nutritional deficiencies | ARIMA(0,2,1) | - | - | - | - | - | - | -0.41 | 0.19 | - | - | - | - | - | - | 2.31 | 0.78 |
| Executions and police conflict | ARIMA(0,2,0) | - | - | - | - | - | - | - | - | - | - | - | - | - | - | 1.15 | 0.41 |
| Acute hepatitis | ARIMA(1,1,0) with drift | 0.66 | 0.13 | - | - | - | - | - | - | - | - | -0.12 | 0.02 | - | - | 2.47 | 0.11 |
| Inflammatory bowel disease | ARIMA(0,2,0) | - | - | - | - | - | - | - | - | - | - | - | - | - | - | 7.03 | 0.53 |
| Aortic aneurysm | ARIMA(0,2,0) | - | - | - | - | - | - | - | - | - | - | - | - | - | - | 8.01 | 0.71 |
| Non-rheumatic valvular heart disease | ARIMA(2,1,0) | 1.52 | 0.16 | -0.58 | 0.16 | - | - | - | - | - | - | - | - | - | - | 1.02 | 0.11 |
| Tetanus | ARIMA(0,2,0) | - | - | - | - | - | - | - | - | - | - | - | - | - | - | 2.90 | 0.38 |
| Rheumatoid arthritis | ARIMA(1,1,0) | 0.83 | 0.09 | - | - | - | - | - | - | - | - | - | - | - | - | 1.42 | 0.87 |
| Bacterial skin diseases | ARIMA(1,1,2) | 0.92 | 0.06 | - | - | - | - | 0.48 | 0.18 | -0.32 | 0.16 | - | - | - | - | 1.95 | 0.15 |
| Gallbladder and biliary diseases | ARIMA(1,1,1) with drift | 0.51 | 0.20 | - | - | - | - | 0.58 | 0.23 | - | - | -0.08 | 0.02 | - | - | 3.20 | 0.47 |
| Interstitial lung disease and pulmonary sarcoidosis | ARIMA(0,2,1) | - | - | - | - | - | - | -0.42 | 0.19 | - | - | - | - | - | - | 1.82 | 0.95 |
| Sexually transmitted infections excluding HIV | ARIMA(1,1,0) with drift | 0.50 | 0.17 | - | - | - | - | - | - | - | - | -0.03 | 0.01 | - | - | 1.89 | 0.49 |
| Bladder cancer | ARIMA(2,1,2) with drift | 1.31 | 0.44 | -0.58 | 0.36 | - | - | 0.37 | 0.29 | 0.57 | 0.43 | -0.05 | 0.01 | - | - | 3.36 | 0.27 |
| Upper respiratory infections | ARIMA(1,1,0) | 0.93 | 0.05 | - | - | - | - | - | - | - | - | - | - | - | - | 7.21 | 0.42 |
| Whooping cough | ARIMA(2,1,0) with drift | 1.01 | 0.22 | -0.39 | 0.21 | - | - | - | - | - | - | -0.12 | 0.03 | - | - | 10.63 | 0.49 |
| Varicella and herpes zoster | ARIMA(1,1,0) | 0.94 | 0.05 | - | - | - | - | - | - | - | - | - | - | - | - | 1.92 | 0.53 |
| Other neglected tropical diseases | ARIMA(1,1,0) with drift | 0.67 | 0.13 | - | - | - | - | - | - | - | - | -0.07 | 0.03 | - | - | 3.59 | 0.56 |
| Pneumoconiosis | ARIMA(1,1,2) with drift | 0.67 | 0.15 | - | - | - | - | 0.60 | 0.19 | 0.39 | 0.17 | -0.07 | 0.02 | - | - | 9.26 | 0.60 |
| Eating disorders | ARIMA(2,0,0) with non-zero mean | 1.11 | 0.17 | -0.31 | 0.18 | - | - | - | - | - | - | - | - | 1.22 | 0.06 | 5.96 | 0.67 |
| Inguinal, femoral, and abdominal hernia | ARIMA(1,1,0) with drift | 0.69 | 0.13 | - | - | - | - | - | - | - | - | -0.07 | 0.02 | - | - | 0.07 | 0.65 |
| Decubitus ulcer | ARIMA(0,2,0) | - | - | - | - | - | - | - | - | - | - | - | - | - | - | 0.17 | 0.68 |
| Multiple sclerosis | ARIMA(3,1,0) with drift | 1.05 | 0.18 | 0.20 | 0.31 | -0.54 | 0.18 | - | - | - | - | -0.06 | 0.03 | - | - | 7.44 | 0.42 |
| Other skin and subcutaneous diseases | ARIMA(0,2,0) | - | - | - | - | - | - | - | - | - | - | - | - | - | - | 21.92 | 0.45 |
| Other intestinal infectious diseases | ARIMA(0,2,0) | - | - | - | - | - | - | - | - | - | - | - | - | - | - | 0.14 | 0.68 |
| Gynecological diseases | ARIMA(1,1,0) | 0.76 | 0.12 | - | - | - | - | - | - | - | - | - | - | - | - | 10.45 | 0.63 |
| Vascular intestinal disorders | ARIMA(1,1,0) | 0.88 | 0.10 | - | - | - | - | - | - | - | - | - | - | - | - | 0.24 | 0.87 |
| Intestinal nematode infections*(no suitable ARIMA model) | - | - | - | - | - | - | - | - | - | - | - | - | - | - | - | - | - |
| Schistosomiasis*(no suitable ARIMA model) | - | - | - | - | - | - | - | - | - | - | - | - | - | - | - | - | - |
| Diphtheria*(no suitable ARIMA model) | - | - | - | - | - | - | - | - | - | - | - | - | - | - | - | - | - |
| Cystic echinococcosis*(no suitable ARIMA model) | - | - | - | - | - | - | - | - | - | - | - | - | - | - | - | - | - |
| Cysticercosis | - | - | - | - | - | - | - | - | - | - | - | - | - | - | - | - | - |
| Dengue | - | - | - | - | - | - | - | - | - | - | - | - | - | - | - | - | - |
| Otitis media | - | - | - | - | - | - | - | - | - | - | - | - | - | - | - | - | - |
| Conflict and terrorism | - | - | - | - | - | - | - | - | - | - | - | - | - | - | - | - | - |
| Malaria | - | - | - | - | - | - | - | - | - | - | - | - | - | - | - | - | - |
| African trypanosomiasis | - | - | - | - | - | - | - | - | - | - | - | - | - | - | - | - | - |
| Chagas disease | - | - | - | - | - | - | - | - | - | - | - | - | - | - | - | - | - |
| Ebola | - | - | - | - | - | - | - | - | - | - | - | - | - | - | - | - | - |
| Leishmaniasis | - | - | - | - | - | - | - | - | - | - | - | - | - | - | - | - | - |
| Yellow fever | - | - | - | - | - | - | - | - | - | - | - | - | - | - | - | - | - |
| Zika virus | - | - | - | - | - | - | - | - | - | - | - | - | - | - | - | - | - |

*ARIMA: Autoregressive Integrated Moving Average model
