# Supplementary material for: Trends in mortality and causes of death among Chinese adolescents aged 10–19 years from 1990 to 2019
Source: Front Public Health. 2023 Feb 7;11:1075858. doi: 10.3389/fpubh.2023.1075858 (PMC9941149; doi:10.3389/fpubh.2023.1075858)
Supplement: Supplementary file 1 [file Data_Sheet_1.ZIP › supplement-xiu/Supplementary Tables and Figures Catalogue.docx]

**Supplementary Tables and Figures Catalogue**

**Supplement 1**

**Table S1.** Death numbers and death rates in 1990 and 2019 in China, ages 10–19 years, both sexes

**Table S2.** Death numbers and death rates in 1990 and 2019 in China, ages 10–19 years, for males

**Table S3.** Death numbers and death rates in 1990 and 2019 in China, ages 10–19 years, for females

**Figure S1.** Percentage of total deaths by level-2 causes in China, aged 10 –19 years, for males from 1990 to 2019

**Figure S2.** Percentage of total deaths by level-2 causes in China, aged 10–19 years, for females from 1990 to 2019

**Figure S3.** Percentage of total deaths by level-2 causes in China, aged 10-14 years from 1990 to 2019

**Figure S4.** Percentage of total deaths by level-2 causes in China, aged 15-19 years from 1990 to 2019

**Figure S5.** Top 25 causes of death in China, aged 10-19 years, for males,1990 and 2019

**Figure S6.** Top 25 causes of death in China, aged 10-19 years, for females,1990 and 2019

**Table S4.** The proportion of level-1 and level-2 causes of death in the China, age 10-19 years, both sexes, 1990 and 2019

**Table S5.** The proportion of level-1 and level-2 causes of death in China, age 10-19 years, for males, 1990 and 2019

**Table S6.** The proportion of level-1 and level-2 causes of death in China, age 10-19 years, for females, 1990 and 2019

**Table S7.** The proportion of level-1 and level-2 causes of death in China, age 10-14 years, 1990 and 2019

**Table S8.** The proportion of level-1 and level-2 causes of death in the China, age 15-19 years, 1990 and 2019

**Table S9.** Predictions of death rate for non-communicable disease in China, aged 10 – 19 years, both sexes

**Table S10.** Predictions of death rate for non-communicable disease in China, aged 10 – 19 years, for males

**Table S11.** Predictions of death rate for non-communicable disease in China, aged 10 – 19 years, for females

**Figure S7.** Rank of the top 25 causes of death in China, aged 10 – 19 years, for males from 2019 to 2030

**Figure S8.** Rank of the top 25 causes of death in China, aged 10 – 19 years, for females from 2019 to 2030

**Supplement 2-Autoregressive Integrated Moving Average model (ARIMA)**

**Supplement 3**

**Table S1.** The orders and coefficients of ARIMA models for the mortality rates of NCDs both sexes.

**Table S2.** The orders and coefficients of ARIMA models for the mortality rates of NCDs in male

**Table S3.** The orders and coefficients of ARIMA models for the mortality rates of NCDs in female

**Supplement 4**

**Table S1.** The orders and coefficients of ARIMA models for 118 causes of death, both sexes

**Table S2.** The orders and coefficients of ARIMA models for 118 causes of death, male

**Table S3.** The orders and coefficients of ARIMA models for 118 causes of death, female
